# Association between maternal adiposity measures and adverse maternal outcomes of pregnancy: Systematic review and meta‐analysis

**DOI:** 10.1111/obr.13449

**Published:** 2022-04-25

**Authors:** Nicola Heslehurst, Lem Ngongalah, Theophile Bigirumurame, Giang Nguyen, Adefisayo Odeniyi, Angela Flynn, Vikki Smith, Lisa Crowe, Becky Skidmore, Laura Gaudet, Alexandre Simon, Louise Hayes

**Affiliations:** ^1^ Population Health Sciences Institute Newcastle University Newcastle upon Tyne UK; ^2^ Department of Nutritional Sciences King's College London London UK; ^3^ Department of Nursing, Midwifery and Health Northumbria University Newcastle upon Tyne UK; ^4^ Independent Information Specialist Ottawa Ontario Canada; ^5^ Department of Obstetrics and Gynaecology Queen's University Kingston Ontario Canada; ^6^ University of Ottawa Ottawa Ontario Canada

**Keywords:** adiposity, maternal, obesity, pregnancy

## Abstract

Maternal obesity increases pregnancy‐related risks. Women with a body mass index (BMI) ≥ 30 kg/m^2^ are considered to be at risk and should receive additional care, although approximately half will have uncomplicated pregnancies. This systematic review aimed to identify early pregnancy measures of adiposity associated with adverse maternal health outcomes. Searches included six databases, reference lists, citations, and contacting authors. Screening and quality assessment were carried out by two authors independently. Random effects meta‐analysis and narrative synthesis were conducted. Seventy studies were included with a pooled sample of 89,588 women. Meta‐analysis showed significantly increased odds of gestational diabetes mellitus (GDM) with higher waist circumference (WC) categories (1.40, 95% confidence interval [CI] 1.04, 1.88) and per unit increase in WC (1.31, 95% CI 1.03, 1.67). Women with GDM had higher WC than controls (mean difference [MD] 6.18 cm, 95% CI 3.92, 8.44). WC was significantly associated with hypertensive disorders, delivery‐related outcomes, metabolic syndrome, and composite pregnancy outcomes. Waist to hip ratio was significantly associated with GDM, hypertensive disorders, and delivery‐related outcomes. Fat mass, neck circumference, skinfolds, and visceral fat were significantly associated with adverse outcomes, although limited data were available. Our findings identify the need to explore how useful adiposity measures are at predicting risk in pregnancy, compared with BMI, to direct care to women with the greatest need.

**Abbreviations**
BMI: body mass index
CI: confidence interval
FFM: fat‐free mass
FM: fat mass
GDM: gestational diabetes mellitus
MD: mean difference
MOOSE: Meta‐analysis Of Observational Studies in Epidemiology
OR: odds ratio
SFT: skinfold thickness
WC: waist circumference
WHR: waist to hip ratio

## 1 INTRODUCTION

The prevalence of maternal obesity, usually defined as a pre‐pregnancy body mass index (BMI) ≥ 30 kg/m^2^, has increased in recent decades. In the United Kingdom, recent data published in 2021 suggest that 22% of women start their pregnancy with a BMI in the obese range,^1^ an increase from 7.6% in 1989 and 15.6% in 2007.^2^ Obesity is associated with an increased risk of multiple adverse pregnancy outcomes that impact on maternal health. These include maternal mortality, gestational diabetes mellitus (GDM), and preeclampsia, as well as long‐term health consequences including the development type 2 diabetes.^3^, ^4^ Guidelines recommend that women with an obese BMI receive additional antenatal care to reduce their risk of an adverse pregnancy outcome.^5^, ^6^, ^7^, ^8^, ^9^ In the context of increasing maternal obesity prevalence, this presents a significant challenge for clinical practice, globally. For example, a national survey of maternity units in England, UK, found that 40% had not implemented guidance to screen all women with a BMI ≥ 30 kg/m^2^ for GDM, primarily due to lack of capacity to do so given the high prevalence of maternal obesity.^10^

Available evidence suggests that the risk of adverse pregnancy outcome associated with obesity has increased over recent years. A large US study using National Center for Health Statistics birth certificate data found the risk of adverse outcomes associated with obesity had increased between 2013 and 2018. In women from all ethnicities studied, odds ratios (ORs) ranged from 1.27 (95% confidence interval [CI] 1.25, 1.29) in non‐Hispanic Black to 1.94 (1.92, 1.96) in non‐Hispanic white women.^11^ These data suggest that current strategies for reducing the clinical risk for women with an obese BMI are not working. The reasons for the failure to reduce risk might be attributable, in part, to guidance using BMI to identify which women require additional routine clinical care during pregnancy, such as GDM screening, and to target behavior change interventions. There has been an abundance of pregnancy weight management interventions that aim to reduce risk of adverse maternal health outcomes, such as GDM. While these interventions appear to be effective in changing maternal behaviors, particularly diet behaviors,^12^ and limiting gestational weight gain and postnatal weight retention,^13^ the evidence base for effectiveness of these interventions is conflicting relating to reducing the risk of maternal health outcomes such as GDM and preeclampsia.^13^ Currently, all women with an obese BMI are considered as being at equal risk of having an adverse pregnancy outcome. However, many women with a BMI ≥ 30 kg/m^2^ will not experience an adverse pregnancy outcome, while a substantial proportion of women with a BMI < 30 kg/m^2^ will.^14^ A multicenter study reported data for uncomplicated pregnancy (defined as normotensive, live birth at >37 weeks, not small for gestational age, and an absence of any other significant pregnancy complications) among 5628 women from the United Kingdom, Ireland, New Zealand, and Australia.^15^ The authors found that 47% of women with an obese BMI had an uncomplicated pregnancy, whereas 42% of women with an overweight BMI (25–29.9 kg/m^2^) did develop pregnancy complications.^15^

The intervention and observational evidence base to date suggests that BMI is not a useful tool to use to predict which women are at high risk of an obesity‐related adverse outcome of pregnancy and therefore require additional care. Body fat distribution was first identified as being important for health in the 1940s,^16^ although there is still debate relating to which measures work best to predict risk. A meta‐analysis identified that using BMI to diagnose obesity demonstrated low sensitivity to identify adiposity, failing to identify half of the people with excess body fat (pooled sensitivity 0.50, 95% CI 0.43, 0.57).^17^ Waist circumference (WC) has been used as an alternative, or alongside, BMI for a number of years as it has been found to be highly correlated with visceral fat.^18^ A large international cardio‐metabolic study reported that the frequent discordance between BMI and WC was driven by the substantial variability in visceral fat for a given BMI.^19^ Although body fat distribution is well established as being important in terms of degree of risk of experiencing a negative health outcome in the general population, it is less clear if body fat distribution is important in terms of predicting risk of an adverse pregnancy outcome. There is some evidence to suggest that central adiposity is important in terms of risk of GDM^20^ and pregnancy hypertension,^21^ but further work to confirm this and to establish which measures of body fat distribution are best at predicting the risk of an adverse pregnancy outcome is needed. This systematic review and meta‐analysis aimed to identify measures of adiposity that are associated with adverse pregnancy outcomes relating to maternal health, in order to assess which may have potential to predict risk better than the current use of BMI.

## 2 METHODS

The systematic review was registered on PROSPERO (CRD42017064464) and the Meta‐analysis Of Observational Studies in Epidemiology (MOOSE) guidelines were followed.^22^

### 2.1 Searches and Screening

A rigorous search strategy was implemented to limit the effect of publication bias, as database searches alone for systematic reviews of observational studies are insufficiently rigorous.^23^ An experienced information specialist (IS) developed the search strategy following an iterative process in consultation with the review team. The MEDLINE strategy was peer reviewed by another experienced IS using the PRESS checklist.^24^ We searched MEDLINE, EMBASE, PsycINFO, CINAHL (EBSCO), JBI Database of Systematic Reviews and Implementation Reports, and Cochrane Library. Using a mixture of controlled search vocabulary (e.g., MeSH) and free text, search terms were derived using the following concepts: “Pregnancy,” “Adiposity,” “Prediction/Risk,” and “Outcomes.” “Outcomes” included generic vocabulary to capture all pregnancy outcomes, as well as specific outcomes of interest (Table S1). Following identification of studies that met the inclusion criteria, all reference lists were hand searched and citation searches were carried out using the Google Scholar cited by feature. Finally, authors of included studies were contacted when additional information was required to assess eligibility for inclusion, or for additional data when required for meta‐analyses (Table S2). Database searches were completed between February 25 and April 2021. Citation and reference list searches and contacting authors were carried until December 2021.

Inclusion criteria were peer‐reviewed studies reporting the association between maternal pre‐ or early‐pregnancy measures of adiposity measured before 20 weeks' gestation and any pregnancy outcomes relating to maternal health, in singleton pregnancies. For the purpose of this review, we classed maternal health outcomes as those that were primarily diagnosed as being a risk to maternal health and well‐being (e.g., GDM and preeclampsia), while recognizing that these outcomes also incur risks to the fetus. Mode of delivery outcomes were also classed as being maternal outcomes in this review. Any outcomes that we classified as being primarily a risk to the fetus or new‐born's health, such as gestational age at birth or birthweight‐related outcomes, will be reported elsewhere. Studies restricted to specific sub‐populations (e.g., adolescents and those with pre‐existing conditions such as polycystic ovarian syndrome or type 2 diabetes) were excluded, with the exception of those who had BMI inclusion criteria as we wanted to explore associations across a range of BMIs. There were no restrictions applied to the country of study or date of publication. Results of screening are reported using the PRISMA statement.^25^

Data extractions were carried out by one researcher using a standardized data extraction protocol (Supplement Information 1), and all data extraction tables were validated by a second researcher (NH, LN, AO, AF, LH, AS, LC, VS). Quality assessments were carried out independently by two researchers using the Newcastle‐Ottawa Scales for cohort and case control studies to assess information bias, selection bias, and confounding.^26^ Any conflicts in data extraction or quality assessment decisions were either resolved by discussion between the two researchers or by a third researcher. Where multiple publications reported data for the same study population, these were further assessed to ensure duplicate data were removed before anlaysis (Supplement Information 2).

### 2.2 Analysis

Each combination of early pregnancy adiposity measure (e.g., WC) and pregnancy outcomes (e.g., GDM) was assessed for ability to pool data in a meta‐analysis. Meta‐analysis was carried out when there were at least three studies reporting data suitable for pooling. Studies that reported binary or continuous exposure variables were synthesized into separated pooled effect meta‐analyses. Similarly, studies that reported mean differences of the adiposity exposure variable within the pregnancy outcome levels were synthesized into a single meta‐analysis. When a categorical adiposity variable had more than two levels (e.g., WC < 80 cm compared with 80–88 and >88 cm), the method proposed by Greenland and Longnecker^27^ was applied to pool estimates for responses at different levels of the adiposity variable. For each category, the respective OR was assigned to each midpoint (the average of the lower and upper bound). The summary ORs were calculated using the random effects model by restricted maximum likelihood.^28^, ^29^ The *I* ^2^ statistic was used to assess the heterogeneity among studies,^30^ with a threshold of >75% representing significant heterogeneity.^31^ Egger's test was used to test publication bias^32^ when the meta‐analysis included at least 10 studies.^33^ Sensitivity analyses were performed by excluding one study at a time from meta‐analysis with at least 10 studies. The statistical analyses were conducted using *dosresmeta* ^34^ and *metaphor* ^35^ packages for R Version 4.0.4.

When meta‐analysis was not possible due to heterogeneity in reporting data, or too few studies, a narrative synthesis was performed following recommendations by Popay et al.^36^ (Supplement Information 3).

## 3 RESULTS

Searches identified 24,027 studies following removal of duplicates; 945 full texts were screened for eligibility, of which 70^20^, ^21^, ^37^, ^38^, ^39^, ^40^, ^41^, ^42^, ^43^, ^44^, ^45^, ^46^, ^47^, ^48^, ^49^, ^50^, ^51^, ^52^, ^53^, ^54^, ^55^, ^56^, ^57^, ^58^, ^59^, ^60^, ^61^, ^62^, ^63^, ^64^, ^65^, ^66^, ^67^, ^68^, ^69^, ^70^, ^71^, ^72^, ^73^, ^74^, ^75^, ^76^, ^77^, ^78^, ^79^, ^80^, ^81^, ^82^, ^83^, ^84^, ^85^, ^86^, ^87^, ^88^, ^89^, ^90^, ^91^, ^92^, ^93^, ^94^, ^95^, ^96^, ^97^, ^98^, ^99^, ^100^, ^101^, ^102^, ^103^, ^104^ met the inclusion criteria (Figure S1). Of these studies, 59 were cohort and 11 were case control studies. Studies were published between 1995 and 2021, with the majority (*n* = 63, 90.0%) published between 2011 and 2021. Sample sizes ranged from 30 to 22,223 women, with a pooled sample size across all studies of 89,588 (Table S3). Study settings were Asia (*n* = 25; China *n* = 9, India *n* = 5, Iran *n* = 4, Turkey *n* = 3, Pakistan *n* = 2, Japan *n* = 1, South Korea *n* = 1), North America (*n* = 17; Canada *n* = 9, the United States *n* = 8), Europe (*n* = 14; the United Kingdom *n* = 6, Italy *n* = 3, multi‐country *n* = 2, Finland *n* = 1, Poland *n* = 1, Russia *n* = 1, Spain *n* = 1), Australia (*n* = 6), Africa (*n* = 3; Ethiopia, Ghana, Nigeria), South America (*n* = 3; Brazil), and one multi‐continent (including data from Australia, New Zealand, the United Kingdom, and Ireland) (Table S3). Ten of the included studies only included women within certain BMI categories; five included only women with obesity (BMI ≥ 30.0 kg/m^2^),^41^, ^52^, ^73^, ^76^, ^97^ two included women with an overweight or obese BMI (≥25.0 kg/m^2^),^61^, ^67^ and three included women with BMI < 30.0 kg/m^2^.^40^, ^48^, ^54^ Early pregnancy WC was the most frequently reported adiposity measure (*n* = 35 studies), followed by waist to hip ratio (WHR) (*n* = 19), measures of fat mass (FM) and fat‐free mass (FFM) (*n* = 15), visceral fat (*n* = 13), subcutaneous fat (*n* = 11), hip circumference (*n* = 7), neck circumference (*n* = 7), arm circumference and skinfold thickness (SFT) (*n* = 5 each), waist to height ratio (*n* = 4), total adipose fat (*n* = 3), leg/thigh circumference and visceral to subcutaneous fat ratio (*n* = 2), and *n* = 1 each for visceral adiposity index, FM index, FFM index, wrist circumference, neck to thigh ratio, waist to thigh ratio, ratio of visceral fat thickness to subcutaneous fat thickness, FM to FFM ratio, combined WC and BMI, combined WHR and BMI, the presence of maternal hepatic fat, and/or the upper quartile of either visceral adipose tissue or total adipose tissue (Table S4). The majority of outcome data related to GDM (*n* = 45 studies), followed by hypertensive disorders (*n* = 20; including preeclampsia and pregnancy‐induced hypertension), measures of insulin and glucose (in the absence of reporting GDM diagnosis, *n* = 7), maternal lipids (*n* = 6), caesarean delivery (*n* = 5), composite outcomes (*n* = 4), induction or assisted deliveries (*n* = 3), metabolic syndrome (*n* = 2), non‐spontaneous labor (*n* = 1), and gestational weight gain (*n* = 1) (Table S4).

The quality of studies ranged from a score of five to eight for both cohort and case control study designs (Table S5). No studies were rated as low quality, and the majority of studies were rated as high quality (76.3% for cohort and 72.7% for case control). Cohort studies consistently scored highly (all >70%) on the representativeness of the exposed cohort (Q1), selection of the non‐exposed cohort (Q2), ascertainment of exposure (Q3), assessment of outcome (Q5), adequate length of follow up (Q6), and adequacy of follow up (Q7) (Table S5A). However, less than half of the cohort studies controlled for gestational weight gain or any other factors in their analysis (Q4, 42.4%). For case control studies, 100% scored highly for questions relating to case definition (Q1), selection and definition of controls (Q3 and Q4), ascertainment of exposure (Q6), and using the same method of ascertainment for cases and controls (Q7) (Table S5B). The lowest scoring question related to representativeness of the cases (Q2, 27.3%), followed by controlling for weight gain or additional factors (Q5, 45.5%) and non‐response rate (Q8, 63.6%).

### 3.1 GDM

There were 45 studies reporting a diagnosis of GDM^20^, ^37^, ^38^, ^39^, ^40^, ^41^, ^43^, ^44^, ^46^, ^47^, ^48^, ^49^, ^51^, ^52^, ^55^, ^56^, ^57^, ^58^, ^59^, ^60^, ^61^, ^62^, ^64^, ^65^, ^66^, ^67^, ^69^, ^71^, ^72^, ^73^, ^76^, ^77^, ^78^, ^80^, ^83^, ^86^, ^88^, ^89^, ^92^, ^95^, ^97^, ^99^, ^101^, ^102^, ^103^ and associations with circumference measures (WC, arm circumference, neck circumference, hip circumference, leg circumference, and wrist circumference), ratios (WHR, waist to height ratio, neck to thigh ratio, and waist to thigh ratio), fat/mass type (visceral fat, subcutaneous fat thickness, FM, FFM, and total adipose tissue), SFT (tricep, bicep, subscapular, suprailiac, abdominal, and sum of SFTs), and combined measure of hepatic fat + visceral adipose tissue quartiles, and hepatic fat and total adipose tissue quartiles (Tables S3 and S4). Meta‐analysis was possible for GDM and WC, WHR, subcutaneous fat thickness, FM, and neck circumference.

#### 3.1.1 WC and GDM

**FIGURE 1:**
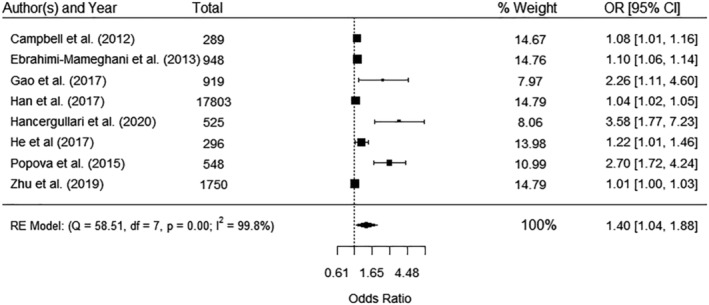
Meta‐analysis of the association between waist circumference categories and gestational diabetes mellitus. Categories of high waist circumference reported by the included studies were >80 cm (Popova et al.,^83^ Gao et al.,^55^ Ebrahimi‐Mameghani et al.,^51^ Zhu et al.,^103^ and Campbell et al.^46^), >84.5 cm (Hancerliogullari et al.^58^), >78.5 cm (Han et al.^57^), and not defined (He et al.^60^). CI, confidence interval; OR, odds ratio; RE, random effect

Fourteen studies reported associations between measures of WC and a diagnosis of GDM^46^, ^51^, ^55^, ^57^, ^58^, ^59^, ^60^, ^72^, ^77^, ^83^, ^86^, ^88^, ^97^, ^103^ and 16 reported case control data for early pregnancy WC between women diagnosed with GDM during pregnancy (cases) and women not diagnosed with GDM (controls).^37^, ^46^, ^47^, ^48^, ^52^, ^57^, ^58^, ^59^, ^60^, ^67^, ^73^, ^78^, ^80^, ^83^, ^88^, ^103^ Nine studies reported categorical measures of WC^37^, ^46^, ^51^, ^55^, ^57^, ^58^, ^60^, ^83^, ^86^, ^103^ and eight could be pooled in the meta‐analysis (Figure 1). There was a significantly increased odds of developing GDM in categories of higher WC (defined as >80, >78.5, and >84.5 cm) compared with lower categories (OR 1.40, 95% CI 1.04, 1.88) with significant heterogeneity (*I* ^2^ 99.8%) (Figure 1). The study^86^ that was not pooled in the meta‐analysis reported the sensitivity, specificity, positive predictive value (PPV), and negative predictive value (NPV) for WC to predict GDM (Table S6A).

**FIGURE 2:**
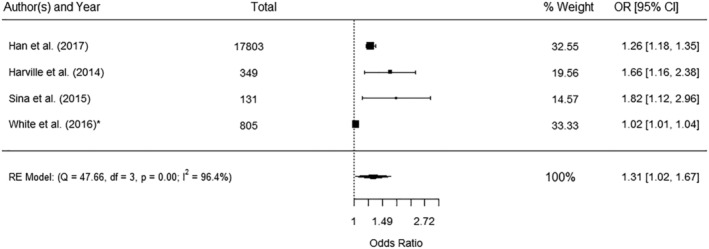
Meta‐analysis of the association between waist circumference as a continuous measure and gestational diabetes mellitus. *Data restricted to women with a body mass index ≥ 30 kg/m^2^. Units of measurement for increase in waist circumference reported by the included studies: 1 standard deviation (Sina et al.,^88^ Han et al.,^57^ and Harville et al.^59^) and 1 cm (White et al.^97^). CI, confidence interval; OR, odds ratio; RE, random effect

Six studies reported WC as a continuous measure and the association with GDM^38^, ^57^, ^59^, ^72^, ^77^, ^88^, ^97^ and four could be pooled in a meta‐analysis (Figure 2). The pooled data showed a significant increase in GDM with every unit of increase in WC (OR 1.31, 95% CI 1.02, 1.67) and significant heterogeneity (*I* ^2^ 96.4%) (Figure 2). The two studies that could not be included in the meta‐analysis reported a significant association between WC and GDM^72^ and an area under the receiving operator curve (AUROC) of 0.74.^77^

**FIGURE 3:**
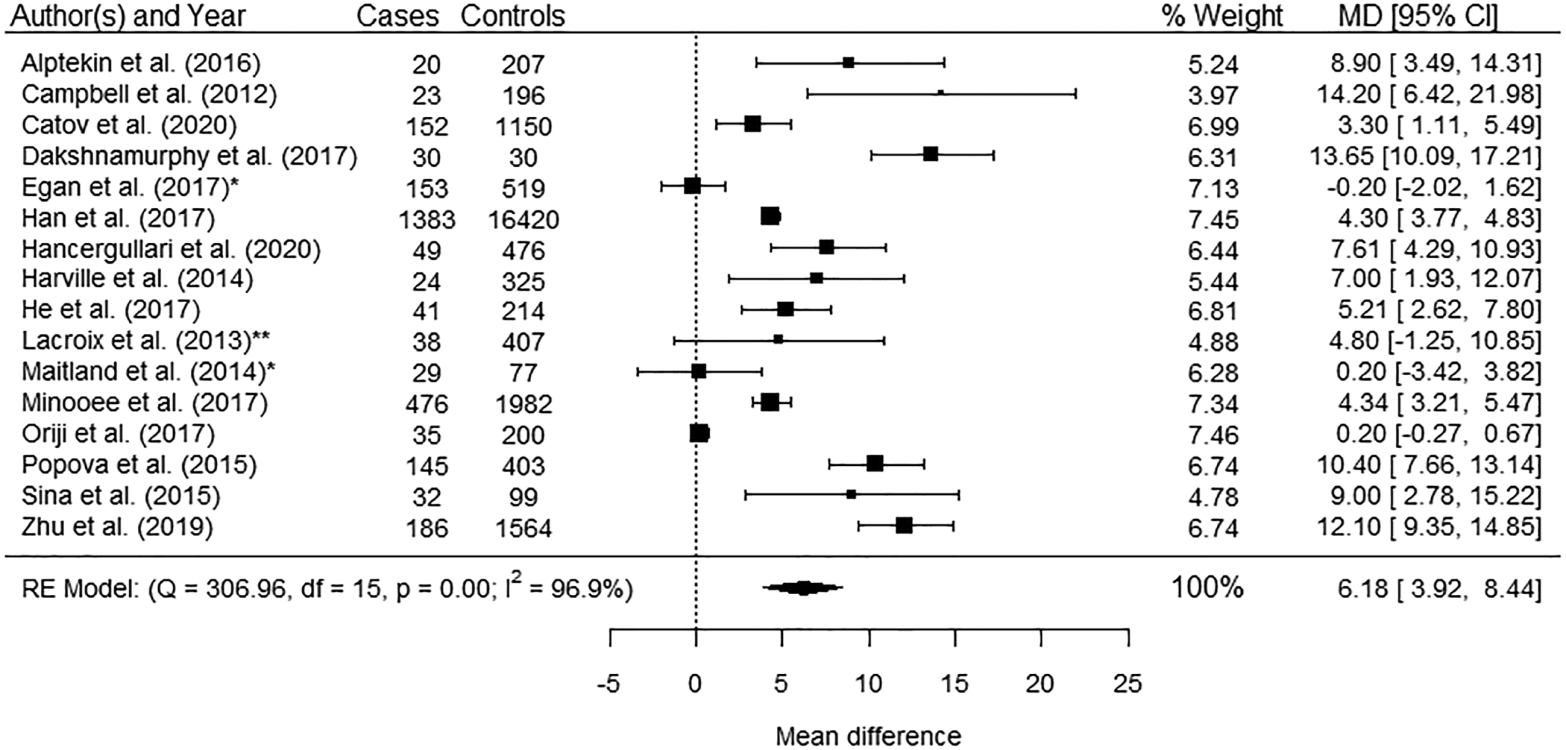
Meta‐analysis of the association between waist circumference (mean differences) and gestational diabetes mellitus. *Data restricted to women with a body mass index ≥ 30 kg/m^2^. **Data restricted to women with a body mass index ≥ 25 kg/m^2^. Dakshnamurthy et al.^48^ excluded women with obesity (for control). CI, confidence interval; MD, mean difference in cm; RE, random effect

Sixteen studies reported case control data and all could be included in a meta‐analysis.^37^, ^46^, ^47^, ^48^, ^52^, ^57^, ^58^, ^59^, ^60^, ^67^, ^73^, ^78^, ^80^, ^83^, ^88^, ^103^ The pooled data showed a significantly increased early pregnancy WC among women diagnosed with GDM compared with those not diagnosed with GDM (mean difference 6.18 cm, 95% CI 3.92, 8.44) (Figure 3). There was significant heterogeneity (*I* ^2^ 96.9%) and evidence of publication bias (*p* = 0.02; Table S6B and Figure S2).

#### 3.1.2 WHR and GDM

There were six studies^43^, ^65^, ^72^, ^95^, ^102^, ^103^ reporting categories of WHR and associations with GDM and all were included in the meta‐analysis. There was a significant increase in odds of GDM for women in the category of high WHR compared with low (OR 2.73, 95% CI 1.67, 4.45) with no significant heterogeneity (*I* ^2^ 43.5%) (Figure S3). One study reported WHR as a continuous measure^88^ and found no significant association with GDM per 1 SD increase (AOR 1.65, 95% CI 0.94, 2.91) (Table S6A). Eight studies reported case control data for mean early pregnancy WHR and GDM^37^, ^41^, ^43^, ^78^, ^88^, ^95^, ^97^, ^103^ (Table S6B). Meta‐analysis showed significantly higher WHR among cases of women diagnosed with GDM compared with controls (mean difference 0.03, 95% CI 0.02, 0.04) with significant heterogeneity (*I* ^2^ 87.5%) (Figure S4).

#### 3.1.3 Subcutaneous fat thickness and GDM

There were three studies^64^, ^89^, ^92^ reporting odds of GDM with continuous measures of subcutaneous fat thickness, with pooled data showing a non‐significant association (OR 1.13, 95% CI 1.00, 1.28) and significant heterogeneity (*I* ^2^ 91.5%) (Figure S5). Two studies^20^, ^99^ reported ORs for categories of subcutaneous fat with no significant associations (AORs comparing high to low categories ranged from 1.2, 95% CI 0.56, 2.7 to 2.96, 95% CI 0.95, 9.25) (Table S6A). One study^44^ reported the AUROC as being 0.69 (95% CI 0.62, 0.76) (Table S6A).

#### 3.1.4 FM and GDM

Eight studies^38^, ^41^, ^61^, ^62^, ^67^, ^71^, ^95^, ^101^ reported case control data for GDM and mean FM (% and kg), mean FM index (kg/m^2^), and FM to FFM ratio (Table S6B). The majority showed significantly increased FM for GDM cases compared with controls (Table S6B). Data from seven studies^38^, ^41^, ^62^, ^67^, ^71^, ^95^, ^101^ reporting FM percent could be pooled in a meta‐analysis that showed significantly higher mean FM percent among cases of GDM compared with controls (mean difference 2.12, 95% CI 1.17, 3.7) with significant heterogeneity (*I* ^2^ 89.8%) (Figure S6). Six studies reported associations between early pregnancy FM and diagnosis of GDM,^40^, ^61^, ^62^, ^71^, ^95^, ^101^ none of which could be pooled in a meta‐analysis (Table S6A). Five^40^, ^62^, ^71^, ^95^, ^101^ reported odds of GDM with continuous or categorical measures of FM (kg and percent), FM to FFM ratio, and FM index; all were significant ranging from AOR 1.07 (95% CI 1.03, 1.13) to OR 2.014 (95% CI 1.64, 2.48). One study reported *R* ^2^ 0.038 (±0.01) for FM percent and GDM.^61^

#### 3.1.5 FFM and GDM

Six studies reported case control data for FFM and GDM^38^, ^61^, ^62^, ^71^, ^95^, ^101^ with conflicting results. Three^38^, ^62^, ^101^ reported mean FFM (kg) and could be pooled in meta‐analysis that showed significantly increased mean difference for cases of GDM compared with controls (mean difference 1.54, 95% CI 0.37, 2.70) with significant heterogeneity (*I* ^2^ 79.1%) (Figure S7). One study reported significantly higher lean leg and arm mass in women with GDM compared with controls and a significantly increased odds of GDM with increasing FFM (kg), and lean arm, leg, and trunk mass^101^ (Table S6B). However, four studies^61^, ^71^, ^95^, ^101^ also reported the opposite direction where significantly higher FFM/lean mass was present in controls compared with cases, and there was a significantly reduced odds of GDM with increasing FFM percent^95^ (Table S6A).

#### 3.1.6 Neck circumference and GDM

Five studies reported case control data for mean neck circumference and GDM,^52^, ^58^, ^60^, ^69^, ^97^ which was significantly higher among cases of GDM compared with controls (mean difference 0.77 cm, 95% CI 0.28, 1.26) with significant heterogeneity (*I* ^2^ 84.4%) (Figure S8). Five studies^58^, ^60^, ^66^, ^69^, ^76^ reported associations between early pregnancy neck circumference and GDM but could not be pooled in meta‐analysis (Table S6A). Three studies^60^, ^76^, ^105^ reported significantly increased AORs for higher neck circumference category or per 1‐cm increase (AOR ranging from 1.15, 95% CI 1.06, 1.24^76^ to 1.84, 95% CI 1.04, 3.25^60^), whereas one study found no significant association between neck circumference category and GDM (AOR 0.83, 95% CI 0.36, 1.91^58^). One study reported a neck circumference cut‐off level of 35.70 cm to predict GDM, with a sensitivity of 51.4% and specificity of 81.2%.^66^

#### 3.1.7 Hip circumference and GDM

Four studies reported data for mean hip circumference among GDM cases and controls,^37^, ^73^, ^78^, ^88^ and pooled data showed no significant difference between cases and controls (2.97 cm, 95% CI −0.96, 6.89), with significant heterogeneity (*I* ^2^ 73.8%) (Figure S9). However, the only data with a negative association were from a study that only included women with an obese BMI.^73^ One study^88^ also reported odds of GDM per 1 SD increase in hip circumference and found no significant association (AOR 1.57, 95% CI 0.99, 2.48) (Table S6A).

Additional GDM data that could not be pooled in meta‐analysis were reported for circumference measures (arm, leg, and wrist circumference), type of fat/mass (visceral fat and total adipose tissue), ratios (waist:height, neck:thigh, and waist:thigh), SFT (abdominal SFT and sum of SFTs), and composite adiposity measure (hepatic fat + visceral fat/total adipose tissue).

#### 3.1.8 Additional circumference measures and GDM

Two studies^95^, ^97^ reported arm circumference and odds of GDM (OR 1.03, 95% CI 0.99, 1.08 and 1.69, 95% CI 1.38, 2.07), and case control data showing arm circumference were significantly increased among GDM cases (Table S6A,B). One study^97^ also reported case control data for thigh and wrist circumference, with a significantly increased wrist circumference for women with GDM, but no significant data for leg circumference (Table S6B).

#### 3.1.9 Type of fat/mass and GDM

Five studies^20^, ^39^, ^44^, ^92^, ^101^ reported data for visceral fat measures and GDM. One^20^ reported significantly increased odds of GDM for women within categories of high versus low visceral fat thickness (>4.8 vs. ≤3.0 cm). Three studies^39^, ^92^, ^101^ reported significantly increased odds of GDM for continuous measures of increasing visceral fat (ranging from AOR 2.0, 95% CI 1.61, 2.50 to OR 2.60, 95% CI 2.46, 2.76). One study^44^ reported an AUROC of 0.69 (95% CI 0.62, 0.77) (Table S6A). Six studies^39^, ^41^, ^48^, ^56^, ^92^, ^101^ reported case control data for visceral fat mass, depth, or visceral adiposity index; all showed significantly increased visceral fat in cases of GDM compared with controls (Table S6B). Two studies reported total adipose tissue and GDM. One reported significantly increased odds for total adipose tissue >7 versus <4.5 cm, but not for measures between 4.6 and 7.0 cm.^20^ One reported an AUROC of 0.70 (95% CI 0.62, 0.77)^44^ (Table S6A).

#### 3.1.10 Ratios and GDM

Two studies^88^, ^97^ reported significantly increased odds of GDM with increasing waist:height (OR 1.57, 95% CI 1.25, 1.98 and AOR 2.29, 95% CI 1.35, 3.88) (Table S6A) and significantly higher waist:height in GDM cases compared with controls (Table S6B). One study^97^ also reported significantly increased odds of GDM with increasing neck:thigh (AOR 1.52, 95% CI 1.11, 2.08) (Table S6A) and significantly higher neck:thigh and waist:thigh in GDM cases compared with controls (Table S6B).

#### 3.1.11 SFT and GDM

Two studies reported significantly increased SFT associated with GDM including increased odds per 1‐mm increase sum of SFT (AOR 1.01, 95% CI 1.01, 1.02)^97^ and abdominal SFT > 20 mm (AOR: 21.71, 95% CI 8.33, 56.63)^80^ (Table S6A). There was also a significantly increased mean triceps, bicep, subscapular, suprailiac, abdominal, and sum of SFT in cases of GDM compared with controls (Table S6B).

#### 3.1.12 Composite adiposity measures and GDM

One study^49^ reported odds of a composite GDM outcome (including impaired fasting glucose, gestational impaired glucose tolerance, or GDM) for a combined measure of hepatic fat + visceral adipose tissue quartiles, and hepatic fat and total adipose tissue quartiles. Both analyses showed that women with hepatic fat present and the highest quartile of visceral and total adipose tissue had significantly increased odds of the composite GDM outcome compared with women in the lowest three quartiles and without hepatic fat (AOR 6.5, 95% CI 2.3, 18.5 and 7.8, 95% CI 2.8, 21.7, respectively) (Table S6A).

#### 3.1.13 Insulin‐ and glucose‐related outcomes in the absence of a GDM diagnosis

Seven studies^42^, ^45^, ^50^, ^74^, ^79^, ^82^, ^93^ reported data relating to glucose or insulin measures that did not also report a diagnosis of GDM and it was not possible to pool these data in a meta‐analysis (Table S7). The outcomes were defined by the studies as being homeostasis model assessment‐insulin resistance (HOMA‐IR), insulin, glucose following glucose tolerance test/oral glucose tolerance test, insulin resistance, insulin sensitivity index, and insulinemia. Adiposity measures were WC, subcutaneous and visceral fat, total adipose tissue, and biceps and triceps SFT. The data were conflicting throughout. Two studies^45^, ^79^ reported HOMA‐IR or insulin measures and WC; one^79^ found significantly increased mean insulin and HOMA‐IR among women with high WC (>90 cm), whereas the other^45^ reported no significant correlation. One study^93^ found no significant associations with WC and blood glucose load. One study reported no significant correlation between subcutaneous fat and glycemia, HOMA‐IR, or insulinemia,^42^ whereas one reported significant associations with HOMA‐IR and insulin sensitivity index.^50^ There were conflicting data across the four studies^42^, ^50^, ^74^, ^82^ reporting visceral fat, and there was no association reported for total adipose tissue. However, visceral fat to subcutaneous fat ratio was significantly correlated with insulinemia and HOMA‐IR in one study.^42^ One study reported significant associations between bicep and triceps SFT and blood glucose following adjustments for confounding factors.^93^

### 3.2 Hypertensive disorders of pregnancy

There were 20 studies reporting data relating to hypertensive disorders of pregnancy including preeclampsia, pregnancy‐induced hypertension, and systolic and diastolic blood pressure,^21^, ^42^, ^51^, ^53^, ^56^, ^63^, ^64^, ^65^, ^72^, ^81^, ^84^, ^86^, ^87^, ^90^, ^91^, ^94^, ^96^, ^98^, ^100^, ^104^ and associations with circumference measures (WC, arm circumference, and hip circumference), ratios (WHR, waist to height ratio, and visceral to subcutaneous fat ratio), fat/mass type (visceral fat, subcutaneous fat, FM, and FFM), and SFT (sum of biceps, triceps, and subscapular) (Table S8). Meta‐analysis was possible for hypertensive disorders and WC and WHR.

#### 3.2.1 WC and hypertensive disorders

**FIGURE 4:**
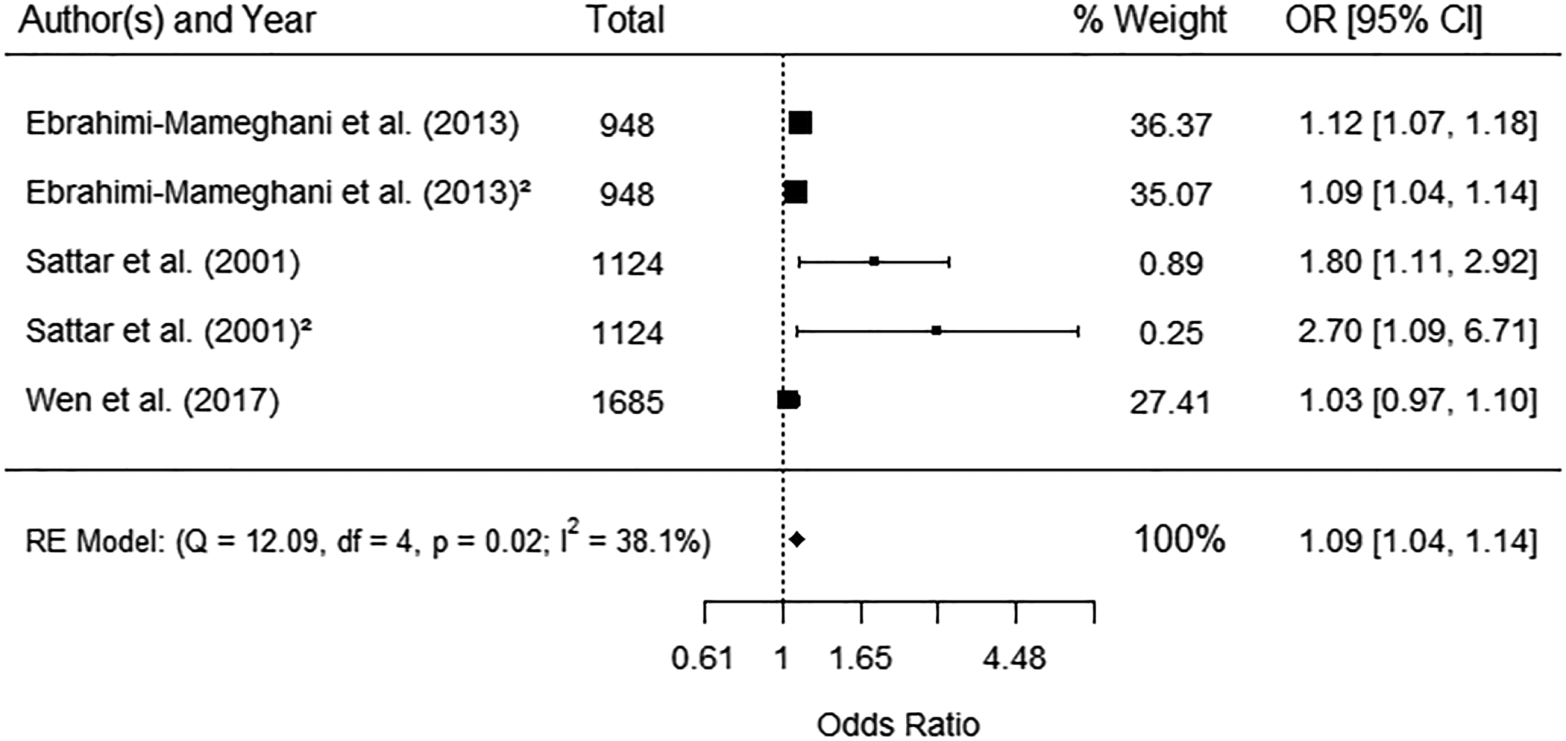
Meta‐analysis of the association between waist circumference categories and hypertensive disorders. Waist circumference categories reported by the studies were ≥80 cm (Ebrahimi‐Mameghani et al.^51^ and Sattar et al.^21^) and ≥65 cm (Wen et al.^104^). Data marked as ^(2)^ were for preeclampsia; other data were pregnancy‐induced hypertension. CI, confidence interval; OR, odds ratio; RE, random effect

Three studies^21^, ^51^, ^104^ reported odds of developing hypertensive disorders in pregnancy and WC categories (defined as ≥80 and ≥65 cm). Pooled data showed significantly increased odds of hypertensive disorders for higher categories of WC (OR 1.09, 95% CI 1.04, 1.14) with no significant heterogeneity (*I* ^2^ 38.1%) (Figure 4). One study^87^ reported significantly increased odds of hypertensive disorders per SD increase in WC (AOR 1.78, 95% CI 1.10, 2.89) (Table S8A). There was also a significant positive correlation reported between WC and hypertension^72^ and diastolic blood pressure, but not systolic blood pressure^56^ (Table S8A). One study^86^ compared using Asian specific and general population criteria to predict gestational hypertension and complications (Table S8A).

**FIGURE 5:**
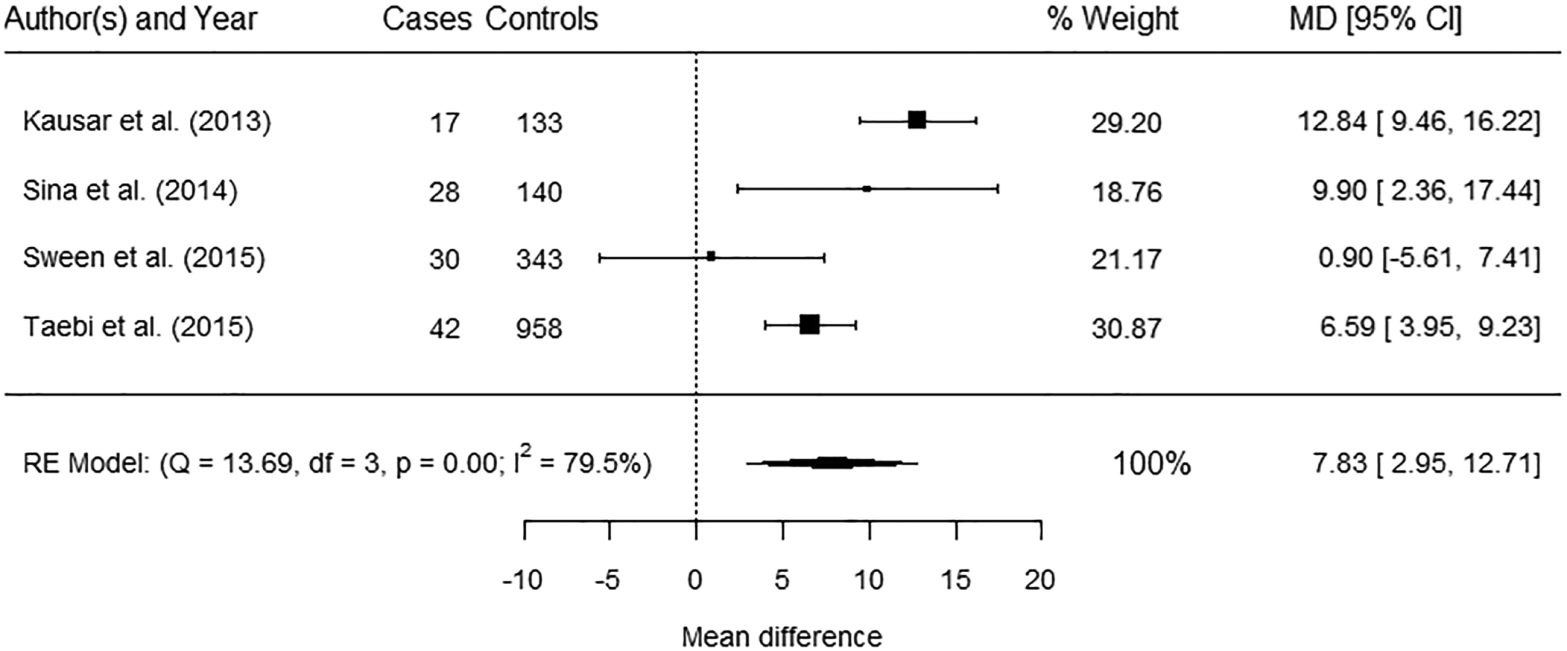
Meta‐analysis of the association between waist circumference (mean differences) and hypertensive disorders. Kausar et al.^63^ and Sina et al.^87^ reported combined category of preeclampsia or gestational hypertension; Sween et al.^90^ and Taebi et al.^91^ reported preeclampsia. CI, confidence interval; MD, mean difference (cm); RE, random effect

Four studies^63^, ^87^, ^90^, ^91^ were pooled in meta‐analysis that showed a significantly higher mean WC among cases of hypertensive disorders compared with controls (mean difference 7.83 cm, 95% CI 3.95, 9.23) with significant heterogeneity (*I* ^2^ 79.5%) (Figure 5). There were also three studies^21^, ^81^, ^94^ reporting median WC; two reported significantly higher WC for cases of preeclampsia^21^ and pregnancy‐induced hypertension,^21^, ^81^ whereas one study stratified their analysis for preeclampsia according to BMI and found no significant difference within groups of women with a recommended or obese BMI (Table S8B).

#### 3.2.2 WHR and hypertensive disorders

Pooled analysis of four studies^65^, ^72^, ^91^, ^98^ reporting categories of WHR showed a significantly increased association between high WHR categories and hypertensive disorders compared with low WHR categories (OR 5.22, 95% CI 1.09, 25.06) with significant heterogeneity (*I* ^2^ 79.3%) (Figure S10). One study^87^ reported odds of developing gestational hypertensive disorders per 1 SD increase in WHR and found no significant association (AOR 1.65, 95% CI 0.80, 3.39) (Table S8A). Five studies^63^, ^87^, ^90^, ^91^, ^98^ were pooled in a meta‐analysis showing a significantly higher mean WHR for cases of hypertensive disorders compared controls (mean difference 0.04, 95% CI 0.02, 0.07) with significant heterogeneity (*I* ^2^ 84.9%) (Figure S11 and Table S8B). Additional hypertensive disorders data that could not be pooled in meta‐analysis were reported for circumference measures (arm and hip), type of fat/mass (subcutaneous fat, visceral fat, FM, and FFM), ratios (waist:height), and SFT (sum of biceps, triceps, and subscapular).

#### 3.2.3 Circumference measures and hypertensive disorders

One study^53^ reported significant associations between the highest category of mid‐upper arm circumference (MUAC, >25 cm), overall preeclampsia (AOR 3.33, 95% CI 1.87, 5.79), and late onset preeclampsia (≥34 weeks, AOR 3.63, 95% CI 1.89, 6.97), but not for early onset preeclampsia (<34 weeks) or for the middle MUAC category (23–24.9 cm) (Table S8A). One study^94^ reported a significantly increased median arm circumference for cases of preeclampsia for women with a recommended BMI, but not for obese BMI (Table S8B). One study^87^ reported odds of any gestational hypertensive disorders per 1 SD increase in hip circumference and found no significant association (AOR 1.53, 95% CI 0.96, 2.52) (Table S8A). Three studies^87^, ^91^, ^94^ reported case control data for hip circumference. One^91^ showed a significantly increased mean hip circumference for women who developed preeclampsia, whereas two studies reported no significant difference for gestational hypertensive disorders^87^ or preeclampsia among women with a recommended or obese BMI^94^ (Table S8B).

#### 3.2.4 Fat/mass type and hypertensive disorders

Three studies^42^, ^56^, ^64^ reported data for subcutaneous fat and three for visceral fat.^42^, ^56^, ^84^ One^64^ found no significant odds of pregnancy‐induced hypertension per 5‐mm increase in subcutaneous fat (AOR 1.03, 95% CI 0.89, 1.18), while one^84^ found a significant increased risk of preeclampsia with preterm birth for visceral fat thickness ≥ 5.2 cm (adjusted risk ratio [ARR] 16.9, 95% CI 1.2, 231.1) but not for preeclampsia overall (ARR 3.4, 95% CI 0.9, 13.4) (Table S8A). Two studies reported no significant correlations between subcutaneous or visceral fat and systolic blood pressure.^42^, ^56^ One study also reported no significant correlation with diastolic blood pressure for either visceral or subcutaneous fat,^56^ whereas the other found significant correlations with both visceral and subcutaneous fat^42^ (Table S8A). Cases of preeclampsia had significantly higher FM than controls^81^, ^90^, ^100^ (Table S8B), and there was a significant association between preeclampsia and categories of high FM (AOR ranging from 1.34, 95% CI 1.01, 2.68 to 6.84, 95% CI 4.15, 41.6)^96^ and per 1% increase in body fat for women with an obese BMI (AOR 1.13, 95% CI 1.01, 1.26), but not for women of any BMI (AOR 1.01, 95% CI 0.97, 1.05)^90^ (Table S8A). There was conflicting evidence for FFM. One study reported significantly lower mean muscle and water mass percentage among women with preeclampsia compared with controls, but no difference in bone density^100^ (Table S8B), and another^96^ found no significant association between high FFM index categories and preeclampsia (Table S8A). Whereas, one study^81^ reported significantly increased FFM, and total body water, among women who developed hypertensive disorders of pregnancy (Table S8B).

#### 3.2.5 Ratios and hypertensive disorders

One study^87^ reported no significant association between waist to height ratio and any gestational hypertensive disorders (AOR 1.44, 95% CI 0.83, 2.51) (Table S8A), and two studies^63^, ^87^ reported mean waist to height ratio for cases of preeclampsia and gestational hypertension with conflicting results (Table S8B).

#### 3.2.6 SFTs and hypertensive disorders

One study^81^ reported significantly higher median sum of SFTs for women who developed hypertensive disorders of pregnancy with appropriate gestational age (Table S8B).

### 3.3 Heterogeneity, publication bias, and sensitivity analysis

There was heterogeneity in 10 out of the 14 meta‐analyses (*I* ^2^ 79.1% to 99.8%). However, given that in most of the analyses there were very few studies, no further analyses were performed to identify factors explaining observed heterogeneity. Sensitivity analyses were performed for meta‐analyses comprising at least 10 studies. The analyses showed that none of the studies did substantially influence the overall direction of association, effect size, statistical significance, or heterogeneity. There was evidence of publication bias in the analyses of WC (mean differences) and GDM (*p* = 0.024).

### 3.4 Narrative synthesis

It was not possible to conduct any meta‐analysis for delivery‐related outcomes, maternal lipids, metabolic syndrome, composite pregnancy outcomes, or gestational weight gain. Data for these outcomes have been synthesized narratively.

#### 3.4.1 Delivery‐related outcomes

Seven studies^55^, ^64^, ^72^, ^75^, ^77^, ^85^, ^89^ reported outcomes relating to the mode delivery including caesarean delivery,^55^, ^64^, ^75^, ^77^, ^89^ instrumental or caesarean delivery (defined as abnormal delivery),^72^ and induction or non‐spontaneous birth^64^, ^72^, ^85^ (Table S9). High category of WC (≥80 cm) was significantly associated with caesarean delivery (AOR 1.71, 95% CI 1.11, 2.63).^55^ Increasing WC was also significantly correlated with abnormal delivery and induction^72^ and had an AUROC of 0.706 for caesarean.^77^ High WHR was significantly associated with caesarean (OR ranging from 1.43, 95% CI 1.08, 1.89 to 1.74, 95% CI 1.35, 2.25^75^; AUROC 0.732^77^), abnormal delivery (OR 8.35, 95% CI 2.79, 25.0) and induction (OR 4.06, 95% CI 1.70, 9.66),^72^ but not non‐spontaneous birth (ORs ranged from 0.94, 95% CI 0.74, 1.19 to 1.11, 95% CI 0.88, 1.40).^85^ One study reported a combined measure of WHR and BMI and showed a significantly increased odds of caesarean delivery for women with a WHR ≥ 0.85 and a BMI ≥ 30 kg/m^2^ (2.48, 95% CI 1.88, 3.28).^75^ Two studies^64^, ^89^reported associations with delivery outcomes per 5‐mm increase in subcutaneous fat; one found a significantly increased odds of caesarean delivery (AOR 1.05, 95% CI 1.03, 1.07),^89^ whereas the other found no significant association with caesarean, assisted delivery, or induction (AOR ranged from 0.94, 95% CI 0.78, 1.13 to 1.09, 95% CI 0.99, 1.2)^64^ (Table S9).

#### 3.4.2 Maternal lipids

Six studies reported data for maternal lipids (including triglycerides [TGs], high‐density lipoprotein‐cholesterol [HDL‐C], low‐density lipoprotein‐cholesterol [LDL‐C], very‐low‐density lipoprotein‐cholesterol [VLDL‐C], total cholesterol, and free fatty acids) and waist and neck circumference, subcutaneous and visceral fat, and WHR.^42^, ^56^, ^65^, ^66^, ^79^, ^82^ The data reported were primarily correlations with mixed results. Women with a higher early pregnancy WC had significantly positive correlation and increased TGs (g/L) before and after an OGTT, but no significant correlation with HDL‐C, LDL‐C, or total cholesterol (Table S10). There was no significant correlation between neck circumference and TGs or total cholesterol. Mixed results were reported for subcutaneous fat and TGs, but no significant correlations with HDL‐C, LDL‐C, cholesterol/HDL‐C, total cholesterol, or free fatty acids. Visceral fat showed a significant positive correlation with TGs, HDL‐C, total cholesterol/HDL‐C ratio, but not for LDL‐C, VLDL‐C, total cholesterol, or free fatty acids. WHR was significantly positively correlated with VLDL‐C, but not TGs, HDL‐C, LDL‐C, total cholesterol, or TGs/LDL or cholesterol/HDL ratios. The ratio of visceral to subcutaneous fat showed a significant positive correlation with TGs and total cholesterol/HDL‐C ratio, but not for HDL‐C, LDL‐C, total cholesterol, and free fatty acids (Table S10).

#### 3.4.3 Metabolic syndrome

Two studies reported case control data for maternal metabolic syndrome during pregnancy and in the immediate postpartum period^56^, ^70^ and waist, arm, and leg circumference, subcutaneous and visceral fat, and triceps and suprailiac SFTs (Table S11). Women who developed metabolic syndrome in pregnancy and postpartum had significantly increased early pregnancy measures of WC and SFT, but mixed results for all other measures. One study^56^ found that both visceral and subcutaneous fat thickness were significantly higher among cases than controls, whereas the other^70^ only found a significant association with subcutaneous fat thickness and postpartum metabolic syndrome. There was a significantly increased arm circumference among women with metabolic syndrome diagnosed in pregnancy but not postpartum, and no significant association with leg circumference.^70^

#### 3.4.4 Composite adverse pregnancy outcomes

There were four studies that reported composite outcomes^54^, ^55^, ^65^, ^89^ (see Table S12 for defintions of outcomes) and WC and a combined measure of WC and BMI^55^; WHR^65^; subcutaneous fat thickness^89^; and FM.^54^ Data reported for women categorized as having high adiposity compared with low adiposity showed significantly increased odds of adverse outcomes for WC (AOR 1.98, 95% CI 1.30, 3.01), combined WC and BMI (AORs ranging from 2.10, 95% CI 1.14, 3.88 to 3.96, 95% CI 2.40, 6.54), and FM (AOR 11.58, 95% CI 1.96, 67.85), but not for WHR (OR 1.43, 95% CI 0.29, 6.97) (Table S12). When adiposity was measured as a continuous exposure, there was also a significantly increased odds with every 5 mm in subcutaneous fat (AOR 1.04, 95% CI 1.01, 1.06),^89^ but not for FM (OR 1.00, 95% CI 0.92, 1.09).^54^ Case control analysis showed no significant difference between early pregnancy FM of women who developed adverse pregnancy outcomes compared with those who did not (Table S12).

#### 3.4.5 Gestational weight gain

Only one study reported gestational weight gain as an outcome,^68^ which was significantly negatively correlated with FM (Pearson's *r* −0.24, *p* < 0.0001) (Table S13).

## 4 DISCUSSION

This systematic review has identified a large body of existing evidence that reports the associations between early pregnancy adiposity measures and maternal health outcomes. Early pregnancy WC was the most frequently reported adiposity measure. Meta‐analysis and narrative synthesis suggest that this is a strong potential predictor variable for adverse maternal health outcomes. WC was consistently significantly associated with GDM, hypertensive disorders, delivery‐related outcomes, metabolic syndrome, and composite adverse pregnancy outcomes. Similarly, WHR shows potential as it was significantly associated with GDM, hypertensive disorders, and delivery‐related outcomes. FM, neck circumference, SFT, measures of visceral fat, arm circumference, and waist to height ratio were also significantly associated with a range of adverse outcomes, although not as frequently reported in the included studies. However, the evidence base was generally conflicting or suggestive of no strong association between subcutaneous fat, FFM, or hip circumference and adverse maternal pregnancy outcomes suggesting that these may have limited use in predicting individual risk.

There was some, albeit limited, evidence that certain measures may or may not be associated with adverse pregnancy outcomes depending on the maternal pre‐pregnancy BMI. For example, arm circumference was significantly associated with hypertensive disorders among women with a recommended BMI but not for those with an obese BMI, while FM appeared to have the reverse association. Current UK guidelines for obesity in the general population^8^ recommend using WC to determine obesity‐related risk for people with a BMI < 35 kg/m^2^. A recent consensus statement from the International Atherosclerosis Society and International Chair on Cardio‐metabolic Risk working group on visceral obesity summarizes the evidence base on risk prediction models (in non‐pregnant populations) using WC and BMI. They suggest that the use of WC as a continuous variable, adjusted for BMI, works better than BMI alone to identify individuals with a high‐risk obesity phenotype and that this is partially explained by the ability of WC to identify adults with increased visceral fat mass.^106^ A similar combination of adiposity measures may be useful in pregnancy. In addition, pregnancy offers a unique opportunity to directly measure abdominal visceral fat using ultrasound at routine antenatal appointments, which could potentially eliminate the need for alternative measurements such as WC to estimate visceral fat mass. However, there were limited data in this review that had been analyzed by BMI sub‐groups, or adjusted adiposity measurements for BMI in risk prediction models, and this warrants further investigation.

Although this review identified a wealth of existing data that could be used to examine how useful early pregnancy adiposity measures are at predicting risk of adverse pregnancy outcomes relating to maternal health, there were some limitations in being able to conduct thorough meta‐analysis. The data reported by the included studies were heterogeneous with a high degree of variation in the way results were presented including ORs, correlations, means, medians, and AUROC. There was also heterogeneity between studies reporting adiposity measures as continuous variables, applying inconsistent category definitions, and a combination of unadjusted and multivariable models that adjusted for a wide range of factors. There was also heterogeneity in the use of adiposity measures that could be combined, for example, some studies collecting WC and height data but not reporting waist to height ratio. There were also differences between studies in the criteria they applied to define the outcomes. In particular, studies reporting GDM prior to the widespread adoption of the IADPSG criteria for diagnosing GDM in 2010 used a range of different diagnostic criteria, which are likely to have identified different groups of women as having GDM.^107^ The heterogeneity in methods of analysis and reporting presents challenges when trying to pool data to directly compare different adiposity measures. Using an individual participant data (IPD) meta‐analysis approach could help to overcome some of these challenges by obtaining the raw data to standardize analysis approaches across studies.^108^, ^109^ IPD meta‐analysis would also facilitate the incorporation of data from additional studies that have not published associations between maternal adiposity and pregnancy outcomes, addressing potential implications of publication bias. For example, there were many studies excluded from this systematic review as they did not report associations between adiposity measures and outcome variables despite collecting these data^110^, ^111^; an IPD meta‐analysis could incorporate the inclusion of these datasets. This alternative approach to meta‐analysis would enable a direct comparison of adiposity measures to determine which might be best at predicting risk of a range of adverse pregnancy outcomes.^108^, ^109^ It would also facilitate comparing these measures with the current use of BMI within the same population of women.

This systematic review has strengths and limitations. The development and implementation of the rigorous search strategy involved experienced information scientists, database searches were supplemented with additional searches, and we contacted authors for additional information when required to maximize the number of studies possible to include in the meta‐analyses. Procedures to minimize human error and subjectivity included duplicate independent screening and quality assessment, and validation of all data extraction. We also transformed data where possible to increase the number of studies possible to be pooled in meta‐analysis. However, a key limitation relates to the significant heterogeneity that was present in all but four meta‐analyses. We had a limited number of studies in each meta‐analysis, which meant we were not able to explore sources of heterogeneity using meta‐regression as was planned. The low number of studies that could be pooled in each individual meta‐analysis also meant that the usefulness of exploring publication bias and performing sensitivity analysis was limited. Finally, although we did not limit our search by type of pregnancy outcome, we identified only a few studies reporting associations between maternal adiposity and delivery outcomes or gestational weight gain, and no studies reporting maternal mental health, hemorrhage, infection, or breastfeeding outcomes, which are all significantly associated with maternal BMI. Future adiposity studies should explore a wider range of outcomes relating to maternal health and well‐being.

The evidence base to date shows that large‐scale behavioral interventions that aim to reduce the risks associated with maternal obesity have been successful at improving maternal behavior and weight‐related outcomes,^12^ which may be viewed as being a public health success, but have yet to consistently significantly reduce the impact of obesity on clinical outcomes such as GDM.^13^ However, there is a consistent direction of effect across multiple meta‐analyses of interventions, which suggests potential for a reduction in risk, although there is a lack of statistical significance.^13^ Therefore, interventions may be more successful in consistently preventing adverse outcomes associated with obesity with better targeting.

A primary aim of prenatal care is to improve health outcomes for both mother and baby. Clinicians have a role to assess the degree of risk for each pregnant woman they see and plan patient centered and individualized care with them. Current clinical guidelines use BMI to determine individual risk in pregnancy, which does not provide an accurate measure of adiposity or individual health risks, and this practice is unlikely to be cost‐effective at preventing adverse outcomes. A large proportion of women will not experience the adverse pregnancy outcomes that population studies show they are significantly at risk of developing with a BMI ≥ 30 kg/m^2^. Yet BMI is used in the clinical context as a screening tool to determine individual risk and which women need additional antenatal care. This could result in unnecessary clinical intervention and reduced birth and care choices for these women. Importantly, this also potentially overlooks women with a BMI < 30 kg/m^2^ who have high adiposity but are not currently deemed to need additional care. This systematic review and meta‐analysis has identified a number of potential early pregnancy adiposity measures that could be used in routine clinical care to identify women at increased risk of adiposity‐related adverse outcomes. Our meta‐analysis has identified some promising evidence to help inform clinical practice, for example, relating to WC and WHR and the risk of GDM. However, further research is needed to explore whether these measures work better than BMI at predicting risk of adverse pregnancy outcomes, or if they could be used in combination with BMI or other predictor variables in a risk prediction model. It is essential that future studies prioritize adiposity measures that can be easily implemented into routine maternity care. Further research should compare these measures to determine which could be used most effectively to direct early intervention to women who need it most, to support the best chance of good pregnancy outcomes.

## CONFLICT OF INTEREST

There are no conflicts of interest for any of the authors.

## Supporting information

Supplement Information 1. Data extraction.
Supplement Information 2. Method of managing duplicate data.
Supplement Information 3. Narrative synthesis methods
Figure S1. PRISMA Flow‐chart of the study selection process.
Figure S2.Funnel plot for publication bias.
Figure S3. Meta‐analysis of the association between waist hip ratio (WHR) categories and GDM.
Figure S4. Meta‐analysis of the association between waist hip ratio (WHR) (mean difference) and GDM.
Figure S5. Meta‐analysis of the association between continuous measures of subcutaneous fat thickness and GDM.
Figure S6. Meta‐analysis of the association between fat mass percent (mean differences) and GDM.
Figure S7. Meta‐analysis of the association between fat free mass (mean differences, kg) and GDM.
Figure S8. Meta‐analysis of the association between neck circumference (mean differences) and GDM.
Figure S9. Meta‐analysis of the association between hip circumference (mean differences) and GDM.
Figure S10. Meta‐analysis of the association between waist hip ratio categories and hypertensive disorders.
Figure S11. Meta‐analysis of the association between waist hip ratio (mean differences) and hypertensive disorders.
Table S1A. Search strategies.
Table S1B. Search strategies‐CINAHL Translation.
Table S2. Contacting authors for additional information.
Table S3. Table of included studies/ study characteristics.
Table S4. Summary of maternal adiposity exposures and maternal outcomes reported.
Table S5. Newcastle Ottawa scale for quality assessment of A) cohort studies; B) case control studies; C) Adapted Newcastle‐Ottawa Scale for Cohort Studies; D) Adapted Newcastle‐Ottawa Scale for Case‐Control Studies.
Table S6. Gestational mellitus diabetes (A for association data and B for case control data reported).
Table S7. Insulin‐ and glucose‐related outcomes (association data) for studies not reporting a GDM diagnosis outcome.
Table S8. Hypertensive disorders of pregnancy (A for association data and B for case control data reported).
Table S9. Delivery‐related outcomes.
Table S10. Maternal lipids.
Table S11. Metabolic syndrome.
Table S12. Composite adverse pregnancy outcomes (A for association data and B for case control data reported)
Table S13. Gestational weight gain.
