# Supplementary material for: Association between maternal adiposity measures and adverse maternal outcomes of pregnancy: Systematic review and meta‐analysis
Source: Obes Rev. 2022 Apr 25;23(7):e13449. doi: 10.1111/obr.13449 (PMC9285432; doi:10.1111/obr.13449)
Supplement: Supplementary file 1 — Supplement Information 1. Data extraction. Supplement Information 2. Method of managing duplicate data. Supplement Information 3. Narrative synthesis methods Figure S1. PRISMA Flow‐chart of the study selection process. Figure S2.Funnel plot for publication bias. Figure S3. Meta‐analysis of the association between waist hip ratio (WHR) categories and GDM. Figure S4. Meta‐analysis of the association between waist hip ratio (WHR) (mean difference) and GDM. Figure S5. Meta‐analysis of the association between continuous measures of subcutaneous fat thickness and GDM. Figure S6. Meta‐analysis of the association between fat mass percent (mean differences) and GDM. Figure S7. Meta‐analysis of the association between fat free mass (mean differences, kg) and GDM. Figure S8. Meta‐analysis of the association between neck circumference (mean differences) and GDM. Figure S9. Meta‐analysis of the association between hip circumference (mean differences) and GDM. Figure S10. Meta‐analysis of the association between waist hip ratio categories and hypertensive disorders. Figure S11. Meta‐analysis of the association between waist hip ratio (mean differences) and hypertensive disorders. Table S1A. Search strategies. Table S1B. Search strategies‐CINAHL Translation. Table S2. Contacting authors for additional information. Table S3. Table of included studies/ study characteristics. Table S4. Summary of maternal adiposity exposures and maternal outcomes reported. Table S5. Newcastle Ottawa scale for quality assessment of A) cohort studies; B) case control studies; C) Adapted Newcastle‐Ottawa Scale for Cohort Studies; D) Adapted Newcastle‐Ottawa Scale for Case‐Control Studies. Table S6. Gestational mellitus diabetes (A for association data and B for case control data reported). Table S7. Insulin‐ and glucose‐related outcomes (association data) for studies not reporting a GDM diagnosis outcome. Table S8. Hypertensive disorders of pregnancy (A for association data and B for case control data r [file OBR-23-0-s001.docx]

**Online Supplementary Material**

**Supplementary information**

Supplement Information 1. Data extraction

Supplement Information 2. Method of managing duplicate data

Supplement Information 3. Narrative synthesis methods

**Supplementary figures:**

Figure S1. PRISMA Flow-chart of the study selection process

Figure S2.Funnel plot for publication bias

Figure S3. Meta-analysis of the association between waist hip ratio (WHR) categories and GDM

Figure S4. Meta-analysis of the association between waist hip ratio (WHR) (mean difference) and GDM.

Figure S5. Meta-analysis of the association between continuous measures of subcutaneous fat thickness and GDM.

Figure S6. Meta-analysis of the association between fat mass percent (mean differences) and GDM.

Figure S7. Meta-analysis of the association between fat free mass (mean differences, kg) and GDM.

Figure S8. Meta-analysis of the association between neck circumference (mean differences) and GDM.

Figure S9. Meta-analysis of the association between hip circumference (mean differences) and GDM.

Figure S10. Meta-analysis of the association between waist hip ratio categories and hypertensive disorders

Figure S11. Meta-analysis of the association between waist hip ratio (mean differences) and hypertensive disorders

**Supplementary tables:**

Table S1A. Search strategies

Table S1B. Search strategies-CINAHL Translation

Table S2. Contacting authors for additional information

Table S3. Table of included studies/ study characteristics

Table S4. Summary of maternal adiposity exposures and maternal outcomes reported

Table S5. Newcastle Ottawa scale for quality assessment of A) cohort studies; B) case control studies; C) Adapted Newcastle-Ottawa Scale for Cohort Studies; D) Adapted Newcastle-Ottawa Scale for Case-Control Studies

Table S6. Gestational mellitus diabetes (A for association data and B for case control data reported)

Table S7. Insulin- and glucose-related outcomes (association data) for studies not reporting a GDM diagnosis outcome

Table S8. Hypertensive disorders of pregnancy (A for association data and B for case control data reported)

Table S9. Delivery-related outcomes

Table S10. Maternal lipids

Table S11. Metabolic syndrome

Table S12. Composite adverse pregnancy outcomes (A for association data and B for case control data reported)

Table S13. Gestational weight gain

**Supplement Information 1. Data Extraction**

Data extraction included information about:

- Study context, design and conduct: year published, country of study, study design, study period of data collection, inclusion and exclusion criteria, and sample size
- Adiposity measures: type and units of measurement, gestational age at time of measurement, and whether any categories were applied and definitions used
- Pregnancy outcomes: type of outcome, how it was defined, and whether any categories were applied and definitions used
- Results reported: the type of data, whether it was adjusted and if so what adjustments were applied
- Relevant data: effect size, means, correlations, estimates of variance, and statistical significance

**Supplement Information 2. Method of managing duplicate data**

Where multiple publications reported data for the same study population, these were further assessed. If publications reported different combinations of adiposity exposure and outcomes, then all data were included as they informed different analysis in this systematic review. If the same combinations of adiposity and outcome were reported, then only data from one publication was included unless these were different types of data that were to be synthesized separately. For example, if one publication reported the odds of developing GDM with increasing waist circumference, and another reported case control data (e.g., mean waist circumference for women diagnosed with GDM and non‐GDM) for the same study population, then both were included in separate analysis. Similarly, duplicate data were also reported within studies. For example, where a study reported unadjusted and adjusted ORs (AORs), then the most adjusted results were used, and if they reported ORs and another type of data (e.g., correlation), then the ORs were prioritized. When conversion of relative risks to ORs was required for meta‐analysis, then unadjusted data were used. If a study reported multiple results for the same outcome using different diagnostic criteria (e.g., GDM diagnosis using the International Association of Diabetes and Pregnancy Study Groups [IADPSG] criteria as well as the Canadian Diabetes Association (CDA) criteria), the data that were most comparable with other studies were included. When studies reported GDM and measures of glucose or insulin, then only GDM data were included. Measures of insulin or glucose were included when there was no corresponding diagnosis of GDM reported for the study population.

**Supplement Information 3. Narrative Synthesis Methods**

All data reported for each included study were tabulated. Separate tables were produced for (i) associations between increasing adiposity and pregnancy outcome (e.g., ORs for GDM with increasing WC, or correlations between adiposity and outcome variables) and (ii) case control data for early pregnancy adiposity (e.g., mean WC for women with or without GDM). The tables were further grouped into thematic categories according to the pregnancy outcomes (e.g., GDM), with subcategories for adiposity measurements (e.g., WC). When required, the data reported by the studies were transformed, if possible, to aid interpretation and to enable comparison of data between studies (e.g., calculation of ORs using frequency data reported in the papers). Patterns in the associations between adiposity measures and maternal health and pregnancy outcomes were described according to the type of data reported (e.g., ORs and CIs, correlations and p values, and means and standard deviations).

**Figure S1. PRISMA Flow-chart of the study selection process**

**Identification of studies via databases and registers**

Records identified from

- Databases (n =22089)
- Additional records identified through other sources (n=1938)

Records removed *before screening*:

Duplicate records removed (n =3005)

**Identification**

Records screened for title and abstracts

(n =21022)

Records excluded

(n = 20052)

Reports excluded (n=875):

- No adiposity measure/BMI only (n=425)
- Not < 20 weeks gestation at measurement (n=81)
- Not in pregnancy (n=84)
- Underweight focus (n=20)
- Not primary research (n=128)
- Restricted population (n=44)
- No data on associations (n=31)
- Not singleton pregnancy (n=2)
- No eligible outcome (n=31)
- No maternal outcomes (n=21)
- Duplicate studies (n=8)

**Screening**

Full texts assessed for eligibility

(n = 945)

Studies included in review

(n =70)

**Included**

Adapted from: Page *et al.* The PRISMA 2020 statement: an updated guideline for reporting systematic reviews. BMJ 2021;372:n71

**Figure S2: Funnel plot for publication bias**

**
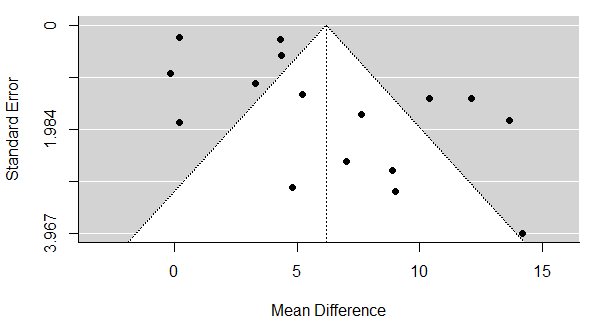
**

**Figure S3: Meta-analysis of the association between waist hip ratio (WHR) categories and GDM**


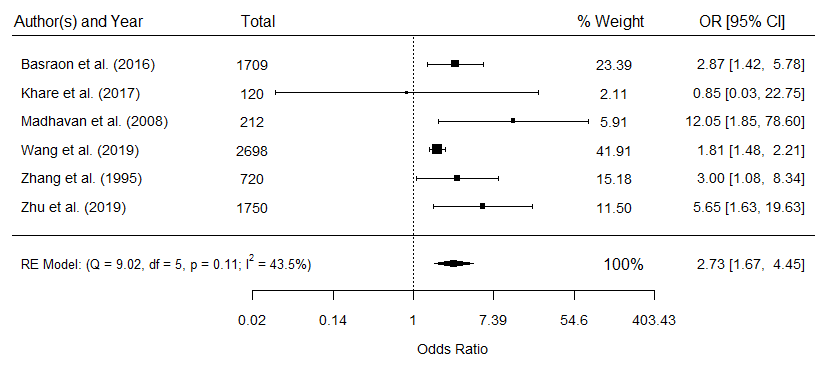


Categories of high waist hip ratio reported by the included studies were >0.80 (Basraon et al. 2016 ^1^, Zhu et al. 2019 ^2^, Khare et al. 2017 ^3^), >0.85 (Madhavan et al. 2008 ^4^, Wang et al. 2019 ^5^), >0.706 (Zhang et al. 1995 ^6^). OR – odds ratio, CI – confidence interval, RE—random effect.

**Figure S4: Meta-analysis of the association between waist hip ratio (WHR) (mean difference) and GDM.**


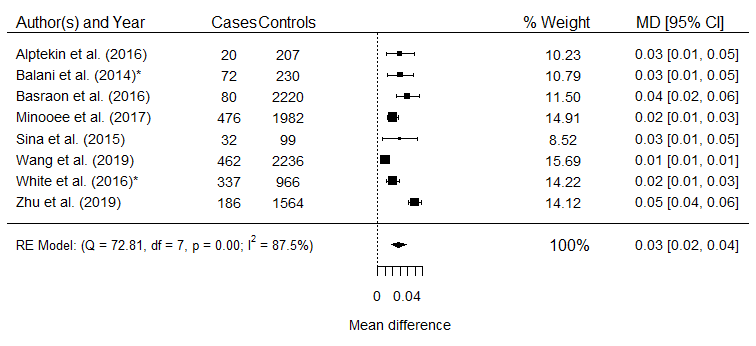


* Data restricted to women with a BMI ≥30kg/m^2^, MD – mean difference, CI – confidence interval, RE—random effect.

(Alptekin et al. 2016 ^7^, Balani et al. (2014) ^8^, Basraon et al. 2016 ^1^, Minooee et al. 2017 ^9^, Sina et al. 2015 ^10^, Wang et al. 2019 ^5^, White et al. 2016 ^11^, Zhu et al. 2019 ^2^)

**Figure S5: Meta-analysis of the association between continuous measures of subcutaneous fat thickness and GDM.**
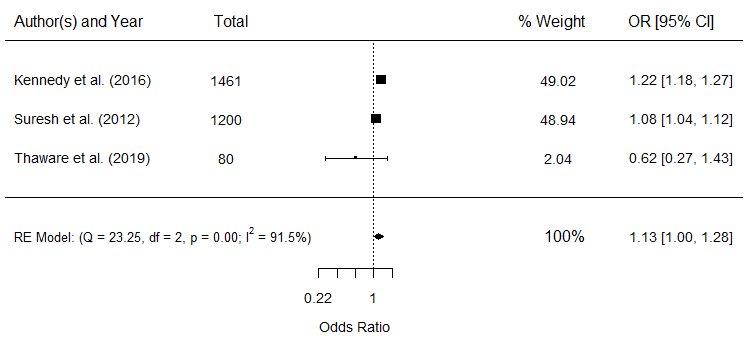


Units of measurement for increase in subcutaneous fat thickness reported by the included studies: 1 standard deviation (Thaware et al. 2019 ^12^), 5mm (Kennedy et al. 2016 ^13^, Suresh et al. 2012 ^14^). OR – odds ratio, CI – confidence interval, RE—random effect.

**Figure S6: Meta-analysis of the association between fat mass percent (mean differences) and GDM.**

*
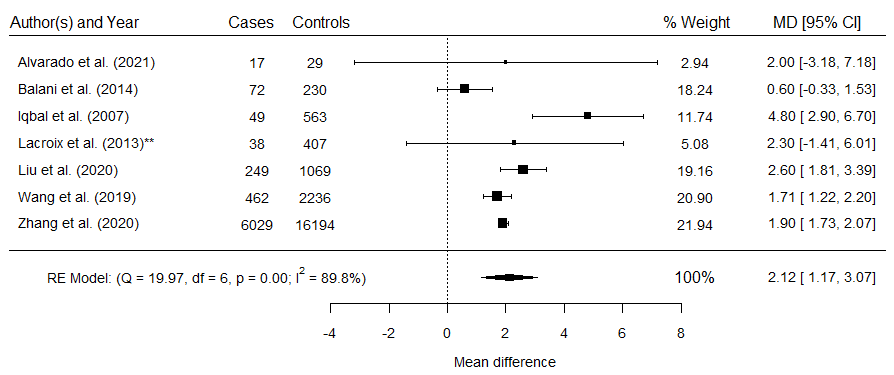
*

^**^ Data restricted to women with a BMI ≥25kg/m^2^, MD – mean difference in percent, CI – confidence interval, RE—random effect.

(Alvarado et al. 2021 ^15^, Balani et al. 2014 ^8^, Iqbal et al. 2007 ^16^, Lacroix et al. 2013 ^17^, Liu et al. 2020 ^18^, Wang et al. 2019 ^5^, Zhang et al. 2020 ^19^)

**Figure S7: Meta-analysis of the association between fat free mass (mean differences, kg) and GDM.**


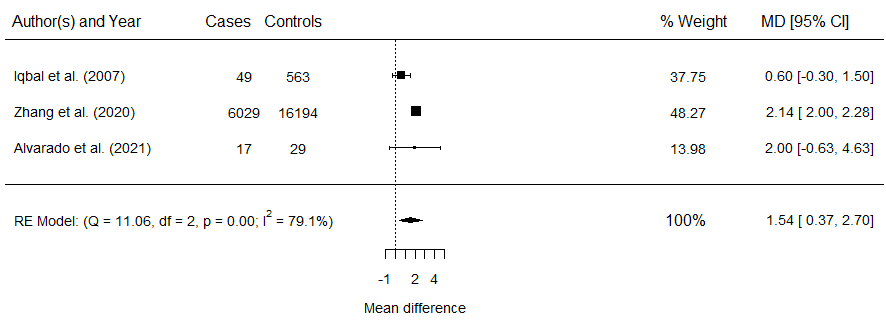


MD – mean difference in cm, CI – confidence interval, RE—random effect.

(Iqbal et al. 2007 ^16^, Zhang et al. 2020 ^19^, Alvarado et al. 2021 ^15^)

**Figure S8: Meta-analysis of the association between neck circumference (mean differences) and GDM.**

*
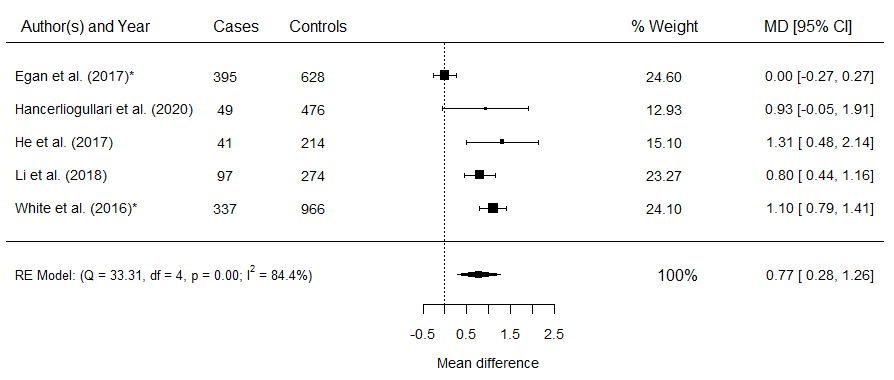
*

^*^ Data restricted to women with a BMI ≥30kg/m^2^, MD – mean difference in cm, CI – confidence interval, RE - random effect.

(Egan et al. 2017 ^20^, Hancerliogullari et al. 2020 ^21^, He et al. 2017 ^22^)

**Figure S9: Meta-analysis of the association between hip circumference (mean differences) and GDM.**


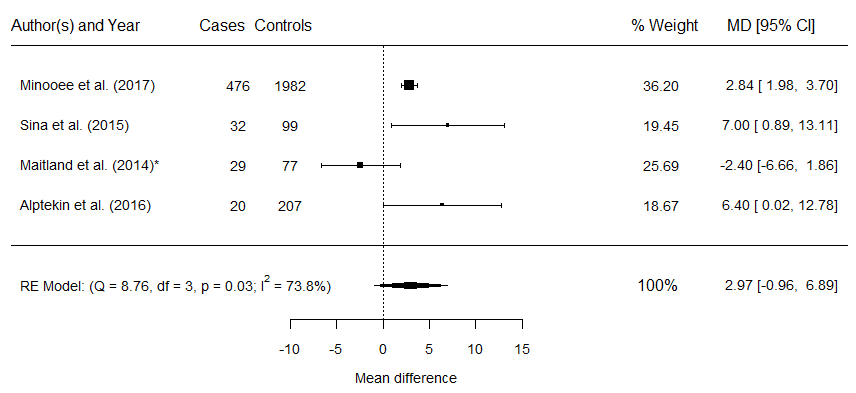


^*^ Data restricted to women with a BMI ≥30kg/m^2^, MD – mean difference in cm, CI – confidence interval, RE—random effect.

(Minooee et al. 2017 ^9^, Sina et al. 2015 ^10^, Maitland et al. 2014 ^23^, Alptekin et al. 2016 ^7^)

**Figure S10: Meta-analysis of the association between waist hip ratio categories and hypertensive disorders**

*
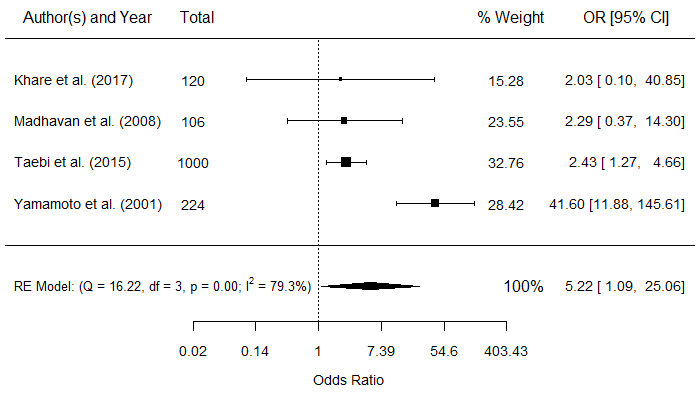
*

Note: Yamamoto et al. 2001 ^24^ and Taebi et al. 2015 ^25^ reported preeclampsia, Khare et al. 2017 ^3^ reported combined outcomes of gestational hypertension or preeclampsia, and Madhavan et al. 2008 ^4^ reported pregnancy induced hypertension. Categories of high waist to hip ratio reported in the papers were ≥0.90 ^24^ and >0.85 ^3, 4, 25^. OR – odds ratio, CI – confidence interval, RE—random effect.

**Figure S11: Meta-analysis of the association between waist hip ratio (mean differences) and hypertensive disorders**

*
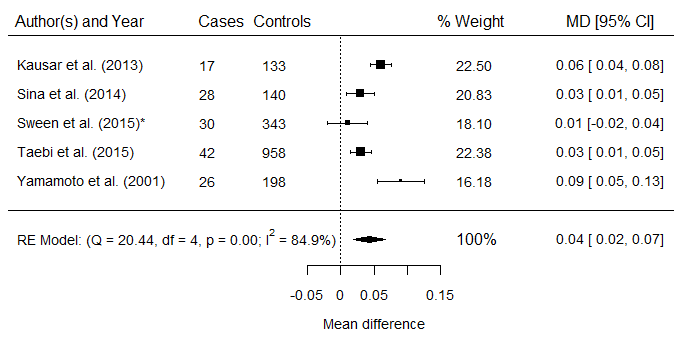
*

Note: Yamamoto et al. 2001 ^24^, Sween et al 2015 ^26^, and Taebi et al. 2015 ^25^ reported preeclampsia, Kausar et al. 2013 ^27^ and Sina et al 2014 ^28^ reported combined outcomes of gestational hypertension or preeclampsia.

* Population was restricted to women with an overweight or obese BMI only. MD – mean difference, CI – confidence interval, RE—random effect.

**Table S1A. Search strategy** Ovid Multifile: Database: Embase Classic+Embase <1947 to 2021 February 24>, Ovid MEDLINE(R) ALL <1946 to February 24, 2021>, APA PsycInfo <1806 to February Week 3 2021>, JBI EBP Database - <Current to Febuary 17, 2021>, EBM Reviews - Cochrane Central Register of Controlled Trials <January 2021>, EBM Reviews - Cochrane Database of Systematic Reviews <2005 to February 19, 2021>

| 1 Pregnancy/ (1686342)  2 exp Pregnancy Complications/ (597252)  3 Pregnant Women/ (89442)  4 exp Pregnancy Trimesters/ (871175)  5 pregnan*.tw,kw. (1401245)  6 Prenatal Care/ (76060)  7 (prenatal* or antenatal* or ante natal* or antepartum or ante partum).tw,kw. (380372)  8 or/1-7 [PREGNANCY] (2423805)  9 exp Overweight/ (830856)  10 (obesity* or obese or overweight or over weight or adiposit*).tw,kw. (974435)  11 (body adj2 (size* or shape* or weight* or fat* or silhouette*)).tw,kw. (760649)  12 exp Adipose Tissue/ (288765)  13 exp Body Fat Distribution/ (23683)  14 ((abdominal* or central* or subcutaneous*) adj2 (obes* or fat* or adipos*)).tw,kw. (108042)  15 Anthropometry/ (102243)  16 Waist Circumference/ or Waist-Hip Ratio/ or Waist-Height Ratio/ (81581)  17 Skinfold Thickness/ (20977)  18 (anthropometr* or BMI or BMIs or skinfold* or SFT or SFTs).tw,kw. (676080)  19 (waist* adj2 (circumference* or hip* or height or thigh*)).tw,kw. (114064)  20 (waisthip or waistheight or waistthigh or WHR or WHtR or WTR).tw,kw. (17640)  21 or/9-20 [ADIPOSITY] (2322966)  22 8 and 21 [ADIPOSITY IN PREGNANCY] (137699)  23 exp Animals/ not (exp Animals/ and Humans/) (19390249)  24 22 not 23 [ANIMAL-ONLY REMOVED] (79735)  25 (comment or editorial or interview or news or newspaper article).pt. (2205148)  26 (letter not (letter and randomized controlled trial)).pt. (2303945)  27 24 not (25 or 26) [OPINION PIECES REMOVED] (77962)  28 exp Risk/ (3923005) 29 (risk or risked or risks or risky or risking or risk-related).tw,kw. (6506677)  30 predict*.tw,kw. (4534400)  31 logistic*.tw,kw. (961926)  32 (logit* adj1 model*).tw,kw. (7361)  33 Prevalence/ (1081509)  34 prevalen*.tw,kw. (2163016)  35 Pregnancy Complications/ep [epidemiology] (13667)  36 or/28-35 [RISK/PREDICTION] (12669657) | 37 27 and 36 (43537)  38 Pregnancy Outcome/ (119502)  39 ((pregnan* or prenatal* or antenatal* or ante natal* or antepartum or ante partum or perinatal* or peripartum) adj3 outcome*).tw,kw. (129900)  40 ((maternal* or mother* or baby or babies or f?etal* or f?etus* or neonat* or newborn*) adj3 outcome*).tw,kw. (98302)  41 exp Pregnancy Complications/mo [mortality] (6953)  42 Maternal Mortality/ (35809)  43 Fetal Mortality/ (5203)  44 Perinatal Mortality/ (17290)  45 (mortalit* or death* or fatal*).tw,kw. (4292002)  46 exp Fetal Death/ (78083)  47 ((baby or babies or f?etal or f?etus* or neonat* or newborn*) adj3 (dead or demise? or died or dying)).tw,kw. (19072)  48 (stillbirth* or stillborn*).tw,kw. (42521)  49 exp Abortion, Spontaneous/ (84338)  50 ((abort* adj2 spontaneous*) or miscarriage? or (recur* adj2 loss*) or (habit* adj2 abort*)).tw,kw. (81076)  51 exp Hospitalization/ (700041)  52 hospitali*.tw,kw. (824437)  53 ((admit* or admission* or readmit* or readmission*) adj3 (hospital? or critical care or intensive care or ICU or ICUs or NICU or NICUs or SICU or SICUs)).tw,kw. (431725)  54 exp Hypertension, Pregnancy-Induced/ (58650)  55 ((eclamp* or hypertensi* or preeclamp* or pre-eclamp* or toxemi* or toxaemi* or EPH or hemolys#s) adj3 (gestational* or maternal* or "new onset" or pregnancy-induced)).tw,kw. (34625)  56 PIH.tw,kw. (5606)  57 HELLP.tw,kw. (7319)  58 exp Diabetes, Gestational/ (54256)  59 ((diabet* or DM or T2DM) adj3 (gestational* or maternal* or "new onset" or pregnancy-induced)).tw,kw. (64220)  60 PID.tw,kw. (14217)  61 macrosomi*.tw,kw. (12013)  62 Venous Thromboembolism/ (52193)  63 (DVT or thromboemboli* or VTE).tw,kw. (204826)  64 exp Placenta Diseases/ (48637)  65 Placental Circulation/ (7393)  66 (placenta* adj3 (abnormal* or disease* or disorder* or dysfunction*)).tw,kw. (14408)  67 (placenta* adj2 mediate*).tw,kw. (1274)  68 Fetal Growth Retardation/ (34241)  69 ((f?etal or f?etus* or intrauterin*) adj grow* adj3 (restrict* or retard*)).tw,kw. (47666) | 70 (FGR or IUGR).tw,kw. (21918)  71 Premature Birth/ (84374)  72 (preterm or prematur*).tw,kw. (567271)  73 exp Cesarean Section/ (159130)  74 (cesarean* or caesarean*).tw,kw. (175345)  75 (C-section* or Csection*).tw,kw. (6087)  76 (abdom* adj3 deliver*).tw,kw. (2515)  77 (postdate* or post-date* or postmatur* or post-matur* or postterm* or post-term*).tw,kw. (9501)  78 (42 week? or 43 week?).tw,kw. (11739)  79 ("42 0/7" or "42 1/7" or "42 2/7" or "42 3/7" or "42 4/7" or "42 5/7" or "42 6/7" or "42 7/7" or "43 0/7" or "43 1/7" or "43 2/7" or "43 3/7" or "43 4/7" or "43 5/7" or "43 6/7" or "43 7/7").tw,kw. (733)  80 Fetal Macrosomia/ (7562)  81 ("large-for-gestational age" or LGA).tw,kw. (10242)  82 Congenital Abnormalities/ (46193)  83 ((birth or congenital) adj3 (abnormal* or anomal* or defect* or deform*)).tw,kw. (169007)  84 exp Neural Tube Defects/ (63956)  85 (NTD or NTDs).tw,kw. (11657)  86 (neural tube? adj2 (defect* or deform* or malform*)).tw,kw. (18950)  87 (acrani* or craniorachischis* or diastematomyeli* or exencephal* or iniencephal* or neurenteric cyst* or neuroenteric cyst* or occult spinal dysraphism* or spinal cord myelodysplasi* or (tethered adj2 cord syndrome*)).tw,kw. (7575)  88 (anencephal* or aprosencephal*).tw,kw. (7717)  89 (congenital*.tw,kw. or cn.fs.) and ((absen* or lack* or missing) adj2 brain*).tw,kw. (129)  90 (congenital*.tw,kw. or cn.fs.) and ((absen* or lack* or missing) adj2 crani$2 vault*).tw,kw. (10)  91 (meroanencephal* or holoanencephal*).tw,kw. (18)  92 (encephalocele or bifid cranium or cephalocele or cerebellar hernia* or cerebral hernia* or cranial meningoencephalocele or craniocele or cranium bifidum or notoencephalocelecranial or tonsillar hernia*).tw,kw. (8674)  93 (rachischisis or schistorrhachis or status dysraphicus).tw,kw. (493)  94 ((cleft or open) adj1 (spine? or spinal)).tw,kw. (636)  95 (spina? adj (bifida? or bifidum? or dysraphia* or dysraphism*)).tw,kw. (22724)  96 dermal sinus*.tw,kw. (1307)  97 (Arnold-Chiari adj1 (syndrome* or malform* or deform*)).tw,kw. (2304)  98 Chiari malformation?.tw,kw. (6605)  99 (Cantrell* adj1 pentalog*).tw,kw. (173)  100 ((Cantrell Haller Ravitch or thoracoabdominal) adj syndrome*).tw,kw. (21) |
| --- | --- | --- |

| 101 exp Cardiovascular Abnormalities/ (436109)  102 ((heart or cardiac* or cardio* or aort* or arter* or ventric* or vascular*) adj3 (abnormal* or anomal* or defect* or deform* or malform*)).tw,kw. (291857)  103 ((septa? or septum) adj3 (abnormal* or anomal* or defect* or deform* or malform*)).tw,kw. (69689)  104 Cleft Lip/ (33168)  105 Cleft Palate/ (48907)  106 (cleft? adj1 (lip or lips or palat*)).tw,kw. (53688)  107 (congenital fissur* adj1 (lip or lips or palat*)).tw,kw. (0)  108 ((orofacial or oro facial) adj1 cleft?).tw,kw. (2768)  109 (labioschis* or cheiloschis* or abioschiz*).tw,kw. (73)  110 (harelip? or hare lip?).tw,kw. (1683)  111 (palatischis* or palatoschis* or palatoschiz* or palatum fissu*).tw,kw. (330)  112 Anorectal Malformations/ (3480)  113 ((anorectal* or ano-rectal*) adj3 (abnormal* or anomal* or atresia* or defect* or deform* or malform* or stenos*)).tw,kw. (6931)  114 ((anus$2 or anal$2) adj3 (abnormal* or anomal* or atresia* or defect* or deform* or malform* or stenos*)).tw,kw. (6453)  115 ((rectum? or rectal$2) adj3 (abnormal* or anomal* or atresia* or defect* or deform* or malform* or stenos*)).tw,kw. (4788)  116 Hydrocephalus/ (66479)  117 hydrocephal*.tw,kw. (72493)  118 (aqueductal stenos* or cerebral ventriculomegal* or Dandy-Walker or Luschka-Magendie foramina atresia* or Hakim$2).tw,kw. (6685)  119 exp Limb Deformities, Congenital/ (76728)  120 ((arm or arms or carpal or carpus or femur* or finger? or foot or feet or fibula? or forearm? or fore arm? or hand or hands or hip or hips or humeral or humerus or knee? or kneecap? or leg or legs or metacarp* or metatars* or patell* or radius or radial or talipedes or talipes or tarsal or tibia? or toe or toes or ulna? or wrist?) adj3 (abnormal* or anomal* or defect* or deform* or malform* or reduc*)).tw,kw. (114261)  121 ((limb or limbs) adj3 (abnormal* or anomal* or defect* or deform* or malform* or reduc*)).tw,kw. (22691)  122 (extremit* adj3 (abnormal* or anomal* or defect* or deform* or malform* or reduc*)).tw,kw. (6470)  123 (clubfoot or club foot or clubfeet or club feet).tw,kw. (8237)  124 (flatfoot or flat foot or flatfeet or flat feet).tw,kw. (5764)  125 (arachnodactyl* or brachydactyl* or ectromelia* or amelia* or hemimelia* or phocomelia* or sirenomelia* or hyperdactyl* or polydactyl* or polysyndactyl* or syndactyl*).tw,kw. (23965)  126 Gastroschisis/ (5218)  127 gastroschis*.tw,kw. (5903)  128 or/38-127 [OUTCOMES] (7495804)  129 37 and 128 (26984)  130 limit 129 to english [Limit not valid in JBI EBP Database -; records were retained] (24886)  131 130 use medall [MEDLINE RECORDS] (11796) | 132 ("20190128" or "20190129" or 2019013* or 201902* or 201903* or 201904* or 201905* or 201906* or 201907* or 201908* or 201909* or 201910* or 201911* or 201912* or 2020* or 2021*).dt. (2905071)  133 131 and 132 [MEDLINE UPDATE] (2204)  134 pregnancy/ (1686342)  135 exp pregnancy complication/ (597252)  136 pregnant woman/ (102656)  137 first trimester pregnancy/ or second trimester pregnancy/ or third trimester pregnancy/ (125195)  138 pregnan*.tw,kw. (1401245)  139 prenatal care/ (76060)  140 (prenatal* or antenatal* or ante natal* or antepartum or ante partum).tw,kw. (380372)  141 or/134-140 [PREGNANCY] (2416774)  142 exp obesity/ (820921)  143 (obesity* or obese or overweight or over weight or adiposit*).tw,kw. (974435)  144 (body adj2 (size* or shape* or weight* or fat* or silhouette*)).tw,kw. (760649)  145 exp adipose tissue/ (288765)  146 ((abdominal* or central* or subcutaneous*) adj2 (obes* or fat* or adipos*)).tw,kw. (108042)  147 anthropometry/ (102243)  148 waist circumference/ or waist hip ratio/ or waist to height ratio/ or weight height ratio/ (81999)  149 skindfold thickness/ (0)  150 (anthropometr* or BMI or BMIs or skinfold* or SFT or SFTs).tw,kw. (676080)  151 (waist* adj2 (circumference* or hip* or height or thigh*)).tw,kw. (114064)  152 (waisthip or waistheight or waistthigh or WHR or WHtR or WTR).tw,kw. (17640)  153 or/142-152 [OBESITY/OVERWEIGHT/ADIPOSITY] (2315766)  154 141 and 153 [OBESITY/OVERWEIGHT/ADIPOSITY IN PREGNANCY] (137245)  155 exp animal experimentation/ or exp models animal/ or exp animal experiment/ or nonhuman/ or exp vertebrate/ (54489254)  156 exp human/ or exp human experimentation/ or exp human experiment/ (43135372)  157 155 not 156 (11355748)  158 154 not 157 [ANIMAL-ONLY REMOVED] (109820)  159 editorial.pt. (1248800)  160 letter.pt. not (letter.pt. and randomized controlled trial/) (2303614)  161 158 not (159 or 160) [OPINION PIECES REMOVED] (107676)  162 risk/ (650833)  163 risk assessment/ (904990)  164 risk factor/ (2041736)  165 (risk or risked or risks or risky or risking or risk-related).tw,kw. (6506677)  166 predict*.tw,kw. (4534400)  167 logistic*.tw,kw. (961926) | 168 (logit* adj1 model*).tw,kw. (7361)  169 prevalence/ (1081509)  170 prevalen*.tw,kw. (2163016)  171 exp pregnancy complication/ep [Epidemiology] (65043)  172 or/162-171 [RISK] (12455624)  173 161 and 172 (62606)  174 pregnancy outcome/ (119502)  175 ((pregnan* or prenatal* or antenatal* or ante natal* or antepartum or ante partum or perinatal* or peripartum) adj3 outcome*).tw,kw. (129900)  176 maternal mortality/ (35809)  177 fetus mortality/ (4686)  178 exp perinatal mortality/ (31574)  179 prenatal mortality/ (356)  180 (mortalit* or death* or fatal*).tw,kw. (4292002)  181 exp fetus death/ (48206)  182 ((baby or babies or f?etal or f?etus* or neonat* or newborn*) adj3 (dead or demise? or died or dying)).tw,kw. (19072)  183 (stillbirth* or stillborn*).tw,kw. (42521)  184 abortion, spontaneous/ (48590)  185 ((abort* adj2 spontaneous*) or miscarriage? or (recur* adj2 loss*) or (habit* adj2 abort*)).tw,kw. (81076)  186 exp hospitalization/ (700041)  187 hospital admission/ (214782)  188 hospitali*.tw,kw. (824437)  189 ((admit* or admission* or readmit* or readmission*) adj3 (hospital? or critical care or intensive care or ICU or ICUs or NICU or NICUs or SICU or SICUs)).tw,kw. (431725)  190 maternal hypertension/ (20037)  191 exp "eclampsia and preeclampsia"/ (68646)  192 ((eclamp* or hypertensi* or preeclamp* or pre-eclamp* or toxemi* or toxaemi* or EPH or hemolys#s) adj3 (gestational* or maternal* or "new onset" or pregnancy-induced)).tw,kw. (34625)  193 PIH.tw,kw. (5606)  194 HELLP syndrome/ (7331)  195 HELLP.tw,kw. (7319)  196 exp pregnancy diabetes mellitus/ (40028)  197 ((diabet* or DM or T2DM) adj3 (gestational* or maternal* or "new onset" or pregnancy-induced)).tw,kw. (64220)  198 PID.tw,kw. (14217)  199 macrosomia/ (7227)  200 macrosomi*.tw,kw. (12013)  201 exp venous thromboembolism/ (182436)  202 (DVT or thromboemboli* or VTE).tw,kw. (204826)  203 exp placenta disorder/ (48637)  204 (placenta* adj3 (abnormal* or disease* or disorder* or dysfunction*)).tw,kw. (14408)  205 (placenta* adj2 mediate*).tw,kw. (1274)  206 exp intrauterine growth retardation/ (63720)  207 ((f?etal or f?etus* or intrauterin*) adj grow* adj3 (restrict* or retard*)).tw,kw. (47666) |
| --- | --- | --- |

| 208 (FGR or IUGR).tw,kw. (21918)  209 preterm birth/ (66134)  210 (preterm or prematur*).tw,kw. (567271)  211 exp cesarean section/ (159130)  212 (cesarean* or caesarean*).tw,kw. (175345)  213 (C-section* or Csection*).tw,kw. (6087)  214 (abdom* adj3 deliver*).tw,kw. (2515)  215 postmaturity/ (1018)  216 (postdate* or post-date* or postmatur* or post-matur* or postterm* or post-term*).tw,kw. (9501)  217 (42 week? or 43 week?).tw,kw. (11739)  218 ("42 0/7" or "42 1/7" or "42 2/7" or "42 3/7" or "42 4/7" or "42 5/7" or "42 6/7" or "42 7/7" or "43 0/7" or "43 1/7" or "43 2/7" or "43 3/7" or "43 4/7" or "43 5/7" or "43 6/7" or "43 7/7").tw,kw. (733)  219 large for gestational age/ (3512)  220 ("large-for-gestational age" or LGA).tw,kw. (10242)  221 congenital disorder/ (90520)  222 ((birth or congenital) adj3 (abnormal* or anomal* or defect* or deform*)).tw,kw. (169007)  223 neural tube defect/ (23168)  224 (NTD or NTDs).tw,kw. (11657)  225 (neural tube? adj2 (defect* or deform* or malform*)).tw,kw. (18950)  226 (acrani* or craniorachischis* or diastematomyeli* or exencephal* or iniencephal* or neurenteric cyst* or neuroenteric cyst* or occult spinal dysraphism* or spinal cord myelodysplasi* or (tethered adj2 cord syndrome*)).tw,kw. (7575)  227 (anencephal* or aprosencephal*).tw,kw. (7717)  228 (congenital*.tw,kw. or cn.fs.) and ((absen* or lack* or missing) adj2 brain*).tw,kw. (129)  229 (congenital*.tw,kw. or cn.fs.) and ((absen* or lack* or missing) adj2 crani$2 vault*).tw,kw. (10)  230 (meroanencephal* or holoanencephal*).tw,kw. (18)  231 (encephalocele or bifid cranium or cephalocele or cerebellar hernia* or cerebral hernia* or cranial meningoencephalocele or craniocele or cranium bifidum or notoencephalocelecranial or tonsillar hernia*).tw,kw. (8674)  232 (rachischisis or schistorrhachis or status dysraphicus).tw,kw. (493)  233 ((cleft or open) adj1 (spine? or spinal)).tw,kw. (636)  234 (spina? adj (bifida? or bifidum? or dysraphia* or dysraphism*)).tw,kw. (22724)  235 dermal sinus*.tw,kw. (1307)  236 (Arnold-Chiari adj1 (syndrome* or malform* or deform*)).tw,kw. (2304)  237 Chiari malformation?.tw,kw. (6605)  238 (Cantrell* adj1 pentalog*).tw,kw. (173)  239 ((Cantrell Haller Ravitch or thoracoabdominal) adj syndrome*).tw,kw. (21)  240 exp cardiovascular malformation/ (232814) | 241 ((heart or cardiac* or cardio* or aort* or arter* or ventric* or vascular*) adj3 (abnormal* or anomal* or defect* or deform* or malform*)).tw,kw. (291857)  242 ((septa? or septum) adj3 (abnormal* or anomal* or defect* or deform* or malform*)).tw,kw. (69689)  243 cleft lip/ (33168)  244 cleft palate/ (48907)  245 (cleft? adj1 (lip or lips or palat*)).tw,kw. (53688)  246 (congenital fissur* adj1 (lip or lips or palat*)).tw,kw. (0)  247 ((orofacial or oro facial) adj1 cleft?).tw,kw. (2768)  248 (abioschiz* or cheiloschis* or labioschis*).tw,kw. (73)  249 (harelip? or hare lip?).tw,kw. (1683)  250 (palatischis* or palatoschis* or palatoschiz* or palatum fissu*).tw,kw. (330)  251 exp anorectal malformation/ (8086)  252 ((anorectal* or ano-rectal*) adj3 (abnormal* or anomal* or atresia* or defect* or deform* or malform* or stenos*)).tw,kw. (6931)  253 ((anus$2 or anal$2) adj3 (abnormal* or anomal* or atresia* or defect* or deform* or malform* or stenos*)).tw,kw. (6453)  254 ((rectum? or rectal$2) adj3 (abnormal* or anomal* or atresia* or defect* or deform* or malform* or stenos*)).tw,kw. (4788)  255 congenital hydrocephalus/ (23115)  256 hydrocephal*.tw,kw. (72493)  257 (aqueductal stenos* or cerebral ventriculomegal* or Dandy-Walker or Luschka-Magendie foramina atresia* or Hakim$2).tw,kw. (6685)  258 exp limb malformation/ (52385)  259 ((arm or arms or carpal or carpus or femur* or finger? or foot or feet or fibula? or forearm? or fore arm? or hand or hands or hip or hips or humeral or humerus or knee? or kneecap? or leg or legs or metacarp* or metatars* or patell* or radius or radial or talipedes or talipes or tarsal or tibia? or toe or toes or ulna? or wrist?) adj3 (abnormal* or anomal* or defect* or deform* or malform* or reduc*)).tw,kw. (114261)  260 ((limb or limbs) adj3 (abnormal* or anomal* or defect* or deform* or malform* or reduc*)).tw,kw. (22691)  261 (extremit* adj3 (abnormal* or anomal* or defect* or deform* or malform* or reduc*)).tw,kw. (6470)  262 (clubfoot or club foot or clubfeet or club feet).tw,kw. (8237)  263 (flatfoot or flat foot or flatfeet or flat feet).tw,kw. (5764)  264 (arachnodactyl* or brachydactyl* or ectromelia* or amelia* or hemimelia* or phocomelia* or sirenomelia* or hyperdactyl* or polydactyl* or polysyndactyl* or syndactyl*).tw,kw. (23965)  265 gastroschisis/ (5218)  266 gastroschis*.tw,kw. (5903)  267 or/174-266 [OUTCOMES] (7489447)  268 173 and 267 (39327)  269 conference abstract.pt. (4047447)  270 268 not 269 [CONFERENCE ABSTRACTS REMOVED] (30604)  271 limit 270 to english [Limit not valid in JBI EBP Database -; records were retained] (28029) | 272 271 use emczd [EMBASE RECORDS] (13993)  273 ("20190128" or "20190129" or 2019013* or 201902* or 201903* or 201904* or 201905* or 201906* or 201907* or 201908* or 201909* or 201910* or 201911* or 201912* or 2020* or 2021*).dc. (4252230)  274 272 and 273 [EMBASE UPDATE] (3276)  275 exp Pregnancy/ (1802902)  276 pregnan*.tw,kw. (1401245)  277 exp Prenatal Care/ (194925)  278 (prenatal* or antenatal* or ante natal* or antepartum or ante partum).tw,kw. (380372)  279 or/275-278 [PREGNANCY] (2421757)  280 exp Overweight/ (830856)  281 (obesity* or obese or overweight or over weight or adiposit*).tw,kw. (974435)  282 (body adj2 (size* or shape* or weight* or fat* or silhouette*)).tw,kw. (760649)  283 Body Fat/ (125428)  284 ((abdominal* or central* or subcutaneous*) adj2 (obes* or fat* or adipos*)).tw,kw. (108042)  285 Anthropometry/ (102243)  286 (anthropometr* or BMI or BMIs or skinfold* or SFT or SFTs).tw,kw. (676080)  287 (waist* adj2 (circumference* or hip* or height or thigh*)).tw,kw. (114064)  288 (waisthip or waistheight or waistthigh or WHR or WHtR or WTR).tw,kw. (17640)  289 or/280-288 [ADIPOSITY] (2234627)  290 279 and 289 [ADIPOSITY IN PREGNANCY] (132867)  291 exp Animals/ not (exp Animals/ and Humans/) (19390249)  292 290 not 291 [ANIMAL-ONLY REMOVED] (77691)  293 Risk Factors/ (1675648)  294 Risk Assessment/ (904990)  295 Predisposition/ (101593)  296 (risk or risked or risks or risky or risking or risk-related).tw,kw. (6506677)  297 predict*.tw,kw. (4534400)  298 logistic*.tw,kw. (961926)  299 (logit* adj1 model*).tw,kw. (7361)  300 prevalen*.tw,kw. (2163016)  301 or/293-300 [RISK/PREDICTION] (12236763)  302 292 and 301 (42513)  303 Pregnancy Outcomes/ (56311)  304 ((pregnan* or prenatal* or antenatal* or ante natal* or antepartum or ante partum or perinatal* or peripartum) adj3 outcome*).tw,kw. (129900)  305 ((maternal* or mother* or baby or babies or f?etal* or f?etus* or neonat* or newborn*) adj3 outcome*).tw,kw. (98302)  306 Mortality Rate/ (124692)  307 (mortalit* or death* or fatal*).tw,kw. (4292002) |
| --- | --- | --- |

| 308 ((baby or babies or f?etal or f?etus* or neonat* or newborn*) adj3 (dead or demise? or died or dying)).tw,kw. (19072)  309 (stillbirth* or stillborn*).tw,kw. (42521)  310 Spontaneous Abortion/ (68320)  311 ((abort* adj2 spontaneous*) or miscarriage? or (recur* adj2 loss*) or (habit* adj2 abort*)).tw,kw. (81076)  312 exp Hospitalization/ (700041)  313 hospitali*.tw,kw. (824437)  314 ((admit* or admission* or readmit* or readmission*) adj3 (hospital? or critical care or intensive care or ICU or ICUs or NICU or NICUs or SICU or SICUs)).tw,kw. (431725)  315 Preeclampsia/ (95190)  316 ((eclamp* or hypertensi* or preeclamp* or pre-eclamp* or toxemi* or toxaemi* or EPH or hemolys#s) adj3 (gestational* or maternal* or "new onset" or pregnancy-induced)).tw,kw. (34625)  317 PIH.tw,kw. (5606)  318 HELLP.tw,kw. (7319)  319 Gestational Diabetes/ (47662)  320 ((diabet* or DM or T2DM) adj3 (gestational* or maternal* or "new onset" or pregnancy-induced)).tw,kw. (64220)  321 PID.tw,kw. (14217)  322 macrosomi*.tw,kw. (12013)  323 Embolisms/ (14384)  324 (DVT or thromboemboli* or VTE).tw,kw. (204826)  325 (placenta* adj3 (abnormal* or disease* or disorder* or dysfunction*)).tw,kw. (14408)  326 (placenta* adj2 mediate*).tw,kw. (1274)  327 ((f?etal or f?etus* or intrauterin*) adj grow* adj3 (restrict* or retard*)).tw,kw. (47666)  328 (FGR or IUGR).tw,kw. (21918)  329 Premature Birth/ (84374)  330 (preterm or prematur*).tw,kw. (567271)  331 Caesarean Birth/ (109611)  332 (cesarean* or caesarean*).tw,kw. (175345)  333 (C-section* or Csection*).tw,kw. (6087)  334 (abdom* adj3 deliver*).tw,kw. (2515)  335 (postdate* or post-date* or postmatur* or post-matur* or postterm* or post-term*).tw,kw. (9501)  336 (42 week? or 43 week?).tw,kw. (11739)  337 ("42 0/7" or "42 1/7" or "42 2/7" or "42 3/7" or "42 4/7" or "42 5/7" or "42 6/7" or "42 7/7" or "43 0/7" or "43 1/7" or "43 2/7" or "43 3/7" or "43 4/7" or "43 5/7" or "43 6/7" or "43 7/7").tw,kw. (733)  338 ("large-for-gestational age" or LGA).tw,kw. (10242)  339 Congenital Disorders/ (90520)  340 ((birth or congenital) adj3 (abnormal* or anomal* or defect* or deform*)).tw,kw. (169007)  341 (NTD or NTDs).tw,kw. (11657)  342 (neural tube? adj2 (defect* or deform* or malform*)).tw,kw. (18950) | 343 (acrani* or craniorachischis* or diastematomyeli* or exencephal* or iniencephal* or neurenteric cyst* or neuroenteric cyst* or occult spinal dysraphism* or spinal cord myelodysplasi* or (tethered adj2 cord syndrome*)).tw,kw. (7575)  344 (anencephal* or aprosencephal*).tw,kw. (7717)  345 (congenital*.tw,kw. or cn.fs.) and ((absen* or lack* or missing) adj2 brain*).tw,kw. (129)  346 (congenital*.tw,kw. or cn.fs.) and ((absen* or lack* or missing) adj2 crani$2 vault*).tw,kw. (10)  347 (meroanencephal* or holoanencephal*).tw,kw. (18)  348 (encephalocele or bifid cranium or cephalocele or cerebellar hernia* or cerebral hernia* or cranial meningoencephalocele or craniocele or cranium bifidum or notoencephalocelecranial or tonsillar hernia*).tw,kw. (8674)  349 (rachischisis or schistorrhachis or status dysraphicus).tw,kw. (493)  350 ((cleft or open) adj1 (spine? or spinal)).tw,kw. (636)  351 Spina Bifida/ (16965)  352 (spina? adj (bifida? or bifidum? or dysraphia* or dysraphism*)).tw,kw. (22724)  353 dermal sinus*.tw,kw. (1307)  354 (Arnold-Chiari adj1 (syndrome* or malform* or deform*)).tw,kw. (2304)  355 Chiari malformation?.tw,kw. (6605)  356 (Cantrell* adj1 pentalog*).tw,kw. (173)  357 ((Cantrell Haller Ravitch or thoracoabdominal) adj syndrome*).tw,kw. (21)  358 ((heart or cardiac* or cardio* or aort* or arter* or ventric* or vascular*) adj3 (abnormal* or anomal* or defect* or deform* or malform*)).tw,kw. (291857)  359 ((septa? or septum) adj3 (abnormal* or anomal* or defect* or deform* or malform*)).tw,kw. (69689)  360 Cleft Palate/ (48907)  361 (cleft? adj1 (lip or lips or palat*)).tw,kw. (53688)  362 (congenital fissur* adj1 (lip or lips or palat*)).tw,kw. (0)  363 ((orofacial or oro facial) adj1 cleft?).tw,kw. (2768)  364 (labioschis* or cheiloschis* or abioschiz*).tw,kw. (73)  365 (harelip? or hare lip?).tw,kw. (1683)  366 (palatischis* or palatoschis* or palatoschiz* or palatum fissu*).tw,kw. (330)  367 ((anorectal* or ano-rectal*) adj3 (abnormal* or anomal* or atresia* or defect* or deform* or malform* or stenos*)).tw,kw. (6931)  368 ((anus$2 or anal$2) adj3 (abnormal* or anomal* or atresia* or defect* or deform* or malform* or stenos*)).tw,kw. (6453)  369 ((rectum? or rectal$2) adj3 (abnormal* or anomal* or atresia* or defect* or deform* or malform* or stenos*)).tw,kw. (4788)  370 Hydrocephalus/ (66479)  371 hydrocephal*.tw,kw. (72493)  372 (aqueductal stenos* or cerebral ventriculomegal* or Dandy-Walker or Luschka-Magendie foramina atresia* or Hakim$2).tw,kw. (6685) | 373 ((arm or arms or carpal or carpus or femur* or finger? or foot or feet or fibula? or forearm? or fore arm? or hand or hands or hip or hips or humeral or humerus or knee? or kneecap? or leg or legs or metacarp* or metatars* or patell* or radius or radial or talipedes or talipes or tarsal or tibia? or toe or toes or ulna? or wrist?) adj3 (abnormal* or anomal* or defect* or deform* or malform* or reduc*)).tw,kw. (114261)  374 ((limb or limbs) adj3 (abnormal* or anomal* or defect* or deform* or malform* or reduc*)).tw,kw. (22691)  375 (extremit* adj3 (abnormal* or anomal* or defect* or deform* or malform* or reduc*)).tw,kw. (6470)  376 (clubfoot or club foot or clubfeet or club feet).tw,kw. (8237)  377 (flatfoot or flat foot or flatfeet or flat feet).tw,kw. (5764)  378 (arachnodactyl* or brachydactyl* or ectromelia* or amelia* or hemimelia* or phocomelia* or sirenomelia* or hyperdactyl* or polydactyl* or polysyndactyl* or syndactyl*).tw,kw. (23965)  379 gastroschis*.tw,kw. (5903)  380 or/303-379 [OUTCOMES] (7221328)  381 302 and 380 (25624)  382 limit 381 to english [Limit not valid in JBI EBP Database -; records were retained] (23637)  383 382 use medall,emczd,coch,cctr,jbi (23016)  384 382 not 383 [PSYCINFO RECORDS] (621)  385 (2019011* or 2019012* or 2019013* or 201902* or 201903* or 201904* or 201905* or 201906* or 201907* or 201908* or 201909* or 201910* or 201911* or 201912* or 2020* or 2021*).up. (37664933)  386 384 and 385 [PSYCINFO UPDATE] (71)  387 Pregnancy/ (1686342)  388 exp Pregnancy Complications/ (597252)  389 Pregnant Women/ (89442)  390 exp Pregnancy Trimesters/ (871175)  391 pregnan*.tw,kw. (1401245)  392 Prenatal Care/ (76060)  393 (prenatal* or antenatal* or ante natal* or antepartum or ante partum).tw,kw. (380372)  394 or/387-393 [PREGNANCY] (2423805)  395 exp Overweight/ (830856)  396 (obesity* or obese or overweight or over weight or adiposit*).tw,kw. (974435)  397 (body adj2 (size* or shape* or weight* or fat* or silhouette*)).tw,kw. (760649)  398 exp Adipose Tissue/ (288765)  399 exp Body Fat Distribution/ (23683)  400 ((abdominal* or central* or subcutaneous*) adj2 (obes* or fat* or adipos*)).tw,kw. (108042)  401 Anthropometry/ (102243)  402 Waist Circumference/ or Waist-Hip Ratio/ or Waist-Height Ratio/ (81581)  403 Skinfold Thickness/ (20977) |
| --- | --- | --- |

| 404 (anthropometr* or BMI or BMIs or skinfold* or SFT or SFTs).tw,kw. (676080)  405 (waist* adj2 (circumference* or hip* or height or thigh*)).tw,kw. (114064)  406 (waisthip or waistheight or waistthigh or WHR or WHtR or WTR).tw,kw. (17640)  407 or/395-406 [ADIPOSITY] (2322966)  408 394 and 407 [ADIPOSITY IN PREGNANCY] (137699)  409 exp Animals/ not (exp Animals/ and Humans/) (19390249)  410 408 not 409 [ANIMAL-ONLY REMOVED] (79735)  411 (comment or editorial or interview or news or newspaper article).pt. (2205148)  412 (letter not (letter and randomized controlled trial)).pt. (2303945)  413 410 not (411 or 412) [OPINION PIECES REMOVED] (77962)  414 exp Risk/ (3923005)  415 (risk or risked or risks or risky or risking or risk-related).tw,kw. (6506677)  416 predict*.tw,kw. (4534400)  417 logistic*.tw,kw. (961926)  418 (logit* adj1 model*).tw,kw. (7361)  419 Prevalence/ (1081509)  420 prevalen*.tw,kw. (2163016)  421 Pregnancy Complications/ep [epidemiology] (13667)  422 or/414-421 [RISK/PREDICTION] (12669657)  423 413 and 422 (43537)  424 Pregnancy Outcome/ (119502)  425 ((pregnan* or prenatal* or antenatal* or ante natal* or antepartum or ante partum or perinatal* or peripartum) adj3 outcome*).tw,kw. (129900)  426 ((maternal* or mother* or baby or babies or f?etal* or f?etus* or neonat* or newborn*) adj3 outcome*).tw,kw. (98302)  427 exp Pregnancy Complications/mo [mortality] (6953)  428 Maternal Mortality/ (35809)  429 Fetal Mortality/ (5203)  430 Perinatal Mortality/ (17290)  431 (mortalit* or death* or fatal*).tw,kw. (4292002)  432 exp Fetal Death/ (78083)  433 ((baby or babies or f?etal or f?etus* or neonat* or newborn*) adj3 (dead or demise? or died or dying)).tw,kw. (19072)  434 (stillbirth* or stillborn*).tw,kw. (42521)  435 exp Abortion, Spontaneous/ (84338)  436 ((abort* adj2 spontaneous*) or miscarriage? or (recur* adj2 loss*) or (habit* adj2 abort*)).tw,kw. (81076)  437 exp Hospitalization/ (700041)  438 hospitali*.tw,kw. (824437)  439 ((admit* or admission* or readmit* or readmission*) adj3 (hospital? or critical care or intensive care or ICU or ICUs or NICU or NICUs or SICU or SICUs)).tw,kw. (431725)  440 exp Hypertension, Pregnancy-Induced/ (58650) | 441 ((eclamp* or hypertensi* or preeclamp* or pre-eclamp* or toxemi* or toxaemi* or EPH or hemolys#s) adj3 (gestational* or maternal* or "new onset" or pregnancy-induced)).tw,kw. (34625)  442 PIH.tw,kw. (5606)  443 HELLP.tw,kw. (7319)  444 exp Diabetes, Gestational/ (54256)  445 ((diabet* or DM or T2DM) adj3 (gestational* or maternal* or "new onset" or pregnancy-induced)).tw,kw. (64220)  446 PID.tw,kw. (14217)  447 macrosomi*.tw,kw. (12013)  448 Venous Thromboembolism/ (52193)  449 (DVT or thromboemboli* or VTE).tw,kw. (204826)  450 exp Placenta Diseases/ (48637)  451 Placental Circulation/ (7393)  452 (placenta* adj3 (abnormal* or disease* or disorder* or dysfunction*)).tw,kw. (14408)  453 (placenta* adj2 mediate*).tw,kw. (1274)  454 Fetal Growth Retardation/ (34241)  455 ((f?etal or f?etus* or intrauterin*) adj grow* adj3 (restrict* or retard*)).tw,kw. (47666)  456 (FGR or IUGR).tw,kw. (21918)  457 Premature Birth/ (84374)  458 (preterm or prematur*).tw,kw. (567271)  459 exp Cesarean Section/ (159130)  460 (cesarean* or caesarean*).tw,kw. (175345)  461 (C-section* or Csection*).tw,kw. (6087)  462 (abdom* adj3 deliver*).tw,kw. (2515)  463 (postdate* or post-date* or postmatur* or post-matur* or postterm* or post-term*).tw,kw. (9501)  464 (42 week? or 43 week?).tw,kw. (11739)  465 ("42 0/7" or "42 1/7" or "42 2/7" or "42 3/7" or "42 4/7" or "42 5/7" or "42 6/7" or "42 7/7" or "43 0/7" or "43 1/7" or "43 2/7" or "43 3/7" or "43 4/7" or "43 5/7" or "43 6/7" or "43 7/7").tw,kw. (733)  466 Fetal Macrosomia/ (7562)  467 ("large-for-gestational age" or LGA).tw,kw. (10242)  468 Congenital Abnormalities/ (46193)  469 ((birth or congenital) adj3 (abnormal* or anomal* or defect* or deform*)).tw,kw. (169007)  470 exp Neural Tube Defects/ (63956)  471 (NTD or NTDs).tw,kw. (11657)  472 (neural tube? adj2 (defect* or deform* or malform*)).tw,kw. (18950)  473 (acrani* or craniorachischis* or diastematomyeli* or exencephal* or iniencephal* or neurenteric cyst* or neuroenteric cyst* or occult spinal dysraphism* or spinal cord myelodysplasi* or (tethered adj2 cord syndrome*)).tw,kw. (7575)  474 (anencephal* or aprosencephal*).tw,kw. (7717)  475 (congenital*.tw,kw. or cn.fs.) and ((absen* or lack* or missing) adj2 brain*).tw,kw. (129) | 476 (congenital*.tw,kw. or cn.fs.) and ((absen* or lack* or missing) adj2 crani$2 vault*).tw,kw. (10)  477 (meroanencephal* or holoanencephal*).tw,kw. (18)  478 (encephalocele or bifid cranium or cephalocele or cerebellar hernia* or cerebral hernia* or cranial meningoencephalocele or craniocele or cranium bifidum or notoencephalocelecranial or tonsillar hernia*).tw,kw. (8674)  479 (rachischisis or schistorrhachis or status dysraphicus).tw,kw. (493)  480 ((cleft or open) adj1 (spine? or spinal)).tw,kw. (636)  481 (spina? adj (bifida? or bifidum? or dysraphia* or dysraphism*)).tw,kw. (22724)  482 dermal sinus*.tw,kw. (1307)  483 (Arnold-Chiari adj1 (syndrome* or malform* or deform*)).tw,kw. (2304)  484 Chiari malformation?.tw,kw. (6605)  485 (Cantrell* adj1 pentalog*).tw,kw. (173)  486 ((Cantrell Haller Ravitch or thoracoabdominal) adj syndrome*).tw,kw. (21)  487 exp Cardiovascular Abnormalities/ (436109)  488 ((heart or cardiac* or cardio* or aort* or arter* or ventric* or vascular*) adj3 (abnormal* or anomal* or defect* or deform* or malform*)).tw,kw. (291857)  489 ((septa? or septum) adj3 (abnormal* or anomal* or defect* or deform* or malform*)).tw,kw. (69689)  490 Cleft Lip/ (33168)  491 Cleft Palate/ (48907)  492 (cleft? adj1 (lip or lips or palat*)).tw,kw. (53688)  493 (congenital fissur* adj1 (lip or lips or palat*)).tw,kw. (0)  494 ((orofacial or oro facial) adj1 cleft?).tw,kw. (2768)  495 (labioschis* or cheiloschis* or abioschiz*).tw,kw. (73)  496 (harelip? or hare lip?).tw,kw. (1683)  497 (palatischis* or palatoschis* or palatoschiz* or palatum fissu*).tw,kw. (330)  498 Anorectal Malformations/ (3480)  499 ((anorectal* or ano-rectal*) adj3 (abnormal* or anomal* or atresia* or defect* or deform* or malform* or stenos*)).tw,kw. (6931)  500 ((anus$2 or anal$2) adj3 (abnormal* or anomal* or atresia* or defect* or deform* or malform* or stenos*)).tw,kw. (6453)  501 ((rectum? or rectal$2) adj3 (abnormal* or anomal* or atresia* or defect* or deform* or malform* or stenos*)).tw,kw. (4788)  502 Hydrocephalus/ (66479)  503 hydrocephal*.tw,kw. (72493)  504 (aqueductal stenos* or cerebral ventriculomegal* or Dandy-Walker or Luschka-Magendie foramina atresia* or Hakim$2).tw,kw. (6685)  505 exp Limb Deformities, Congenital/ (76728) |
| --- | --- | --- |

| 506 ((arm or arms or carpal or carpus or femur* or finger? or foot or feet or fibula? or forearm? or fore arm? or hand or hands or hip or hips or humeral or humerus or knee? or kneecap? or leg or legs or metacarp* or metatars* or patell* or radius or radial or talipedes or talipes or tarsal or tibia? or toe or toes or ulna? or wrist?) adj3 (abnormal* or anomal* or defect* or deform* or malform* or reduc*)).tw,kw. (114261)  507 ((limb or limbs) adj3 (abnormal* or anomal* or defect* or deform* or malform* or reduc*)).tw,kw. (22691)  508 (extremit* adj3 (abnormal* or anomal* or defect* or deform* or malform* or reduc*)).tw,kw. (6470)  509 (clubfoot or club foot or clubfeet or club feet).tw,kw. (8237)  510 (flatfoot or flat foot or flatfeet or flat feet).tw,kw. (5764)  511 (arachnodactyl* or brachydactyl* or ectromelia* or amelia* or hemimelia* or phocomelia* or sirenomelia* or hyperdactyl* or polydactyl* or polysyndactyl* or syndactyl*).tw,kw. (23965)  512 Gastroschisis/ (5218)  513 gastroschis*.tw,kw. (5903)  514 or/424-513 [OUTCOMES] (7495804)  515 423 and 514 (26984)  516 limit 515 to english [Limit not valid in JBI EBP Database -; records were retained] (24886)  517 516 use jbi [J BRIGGS RECORDS] (111)  518 ("2019" or "2020" or "2021").yr. (7386168)  519 517 and 518 [J BRIGGS UPDATE] (43)  520 Pregnancy/ (1686342)  521 exp Pregnancy Complications/ (597252)  522 Pregnant Women/ (89442)  523 exp Pregnancy Trimesters/ (871175)  524 pregnan*.tw,kw. (1401245)  525 Prenatal Care/ (76060)  526 (prenatal* or antenatal* or ante natal* or antepartum or ante partum).tw,kw. (380372)  527 or/520-526 [PREGNANCY] (2423805)  528 exp Overweight/ (830856)  529 (obesity* or obese or overweight or over weight or adiposit*).tw,kw. (974435)  530 (body adj2 (size* or shape* or weight* or fat* or silhouette*)).tw,kw. (760649)  531 exp Adipose Tissue/ (288765)  532 exp Body Fat Distribution/ (23683)  533 ((abdominal* or central* or subcutaneous*) adj2 (obes* or fat* or adipos*)).tw,kw. (108042)  534 Anthropometry/ (102243)  535 Waist Circumference/ or Waist-Hip Ratio/ or Waist-Height Ratio/ (81581)  536 Skinfold Thickness/ (20977)  537 (anthropometr* or BMI or BMIs or skinfold* or SFT or SFTs).tw,kw. (676080) | 538 (waist* adj2 (circumference* or hip* or height or thigh*)).tw,kw. (114064)  539 (waisthip or waistheight or waistthigh or WHR or WHtR or WTR).tw,kw. (17640)  540 or/528-539 [ADIPOSITY] (2322966)  541 527 and 540 [ADIPOSITY IN PREGNANCY] (137699)  542 exp Animals/ not (exp Animals/ and Humans/) (19390249)  543 541 not 542 [ANIMAL-ONLY REMOVED] (79735)  544 (comment or editorial or interview or news or newspaper article).pt. (2205148)  545 (letter not (letter and randomized controlled trial)).pt. (2303945)  546 543 not (544 or 545) [OPINION PIECES REMOVED] (77962)  547 exp Risk/ (3923005)  548 (risk or risked or risks or risky or risking or risk-related).tw,kw. (6506677)  549 predict*.tw,kw. (4534400)  550 logistic*.tw,kw. (961926)  551 (logit* adj1 model*).tw,kw. (7361)  552 Prevalence/ (1081509)  553 prevalen*.tw,kw. (2163016)  554 Pregnancy Complications/ep [epidemiology] (13667)  555 or/547-554 [RISK/PREDICTION] (12669657)  556 546 and 555 (43537)  557 Pregnancy Outcome/ (119502)  558 ((pregnan* or prenatal* or antenatal* or ante natal* or antepartum or ante partum or perinatal* or peripartum) adj3 outcome*).tw,kw. (129900)  559 ((maternal* or mother* or baby or babies or f?etal* or f?etus* or neonat* or newborn*) adj3 outcome*).tw,kw. (98302)  560 exp Pregnancy Complications/mo [mortality] (6953)  561 Maternal Mortality/ (35809)  562 Fetal Mortality/ (5203)  563 Perinatal Mortality/ (17290)  564 (mortalit* or death* or fatal*).tw,kw. (4292002)  565 exp Fetal Death/ (78083)  566 ((baby or babies or f?etal or f?etus* or neonat* or newborn*) adj3 (dead or demise? or died or dying)).tw,kw. (19072)  567 (stillbirth* or stillborn*).tw,kw. (42521)  568 exp Abortion, Spontaneous/ (84338)  569 ((abort* adj2 spontaneous*) or miscarriage? or (recur* adj2 loss*) or (habit* adj2 abort*)).tw,kw. (81076)  570 exp Hospitalization/ (700041)  571 hospitali*.tw,kw. (824437)  572 ((admit* or admission* or readmit* or readmission*) adj3 (hospital? or critical care or intensive care or ICU or ICUs or NICU or NICUs or SICU or SICUs)).tw,kw. (431725)  573 exp Hypertension, Pregnancy-Induced/ (58650)  574 ((eclamp* or hypertensi* or preeclamp* or pre-eclamp* or toxemi* or toxaemi* or EPH or hemolys#s) adj3 (gestational* or maternal* or "new onset" or pregnancy-induced)).tw,kw. (34625) | 575 PIH.tw,kw. (5606)  576 HELLP.tw,kw. (7319)  577 exp Diabetes, Gestational/ (54256)  578 ((diabet* or DM or T2DM) adj3 (gestational* or maternal* or "new onset" or pregnancy-induced)).tw,kw. (64220)  579 PID.tw,kw. (14217)  580 macrosomi*.tw,kw. (12013)  581 Venous Thromboembolism/ (52193)  582 (DVT or thromboemboli* or VTE).tw,kw. (204826)  583 exp Placenta Diseases/ (48637)  584 Placental Circulation/ (7393)  585 (placenta* adj3 (abnormal* or disease* or disorder* or dysfunction*)).tw,kw. (14408)  586 (placenta* adj2 mediate*).tw,kw. (1274)  587 Fetal Growth Retardation/ (34241)  588 ((f?etal or f?etus* or intrauterin*) adj grow* adj3 (restrict* or retard*)).tw,kw. (47666)  589 (FGR or IUGR).tw,kw. (21918)  590 Premature Birth/ (84374)  591 (preterm or prematur*).tw,kw. (567271)  592 exp Cesarean Section/ (159130)  593 (cesarean* or caesarean*).tw,kw. (175345)  594 (C-section* or Csection*).tw,kw. (6087)  595 (abdom* adj3 deliver*).tw,kw. (2515)  596 (postdate* or post-date* or postmatur* or post-matur* or postterm* or post-term*).tw,kw. (9501)  597 (42 week? or 43 week?).tw,kw. (11739)  598 ("42 0/7" or "42 1/7" or "42 2/7" or "42 3/7" or "42 4/7" or "42 5/7" or "42 6/7" or "42 7/7" or "43 0/7" or "43 1/7" or "43 2/7" or "43 3/7" or "43 4/7" or "43 5/7" or "43 6/7" or "43 7/7").tw,kw. (733)  599 Fetal Macrosomia/ (7562)  600 ("large-for-gestational age" or LGA).tw,kw. (10242)  601 Congenital Abnormalities/ (46193)  602 ((birth or congenital) adj3 (abnormal* or anomal* or defect* or deform*)).tw,kw. (169007)  603 exp Neural Tube Defects/ (63956)  604 (NTD or NTDs).tw,kw. (11657)  605 (neural tube? adj2 (defect* or deform* or malform*)).tw,kw. (18950)  606 (acrani* or craniorachischis* or diastematomyeli* or exencephal* or iniencephal* or neurenteric cyst* or neuroenteric cyst* or occult spinal dysraphism* or spinal cord myelodysplasi* or (tethered adj2 cord syndrome*)).tw,kw. (7575)  607 (anencephal* or aprosencephal*).tw,kw. (7717)  608 (congenital*.tw,kw. or cn.fs.) and ((absen* or lack* or missing) adj2 brain*).tw,kw. (129)  609 (congenital*.tw,kw. or cn.fs.) and ((absen* or lack* or missing) adj2 crani$2 vault*).tw,kw. (10)  610 (meroanencephal* or holoanencephal*).tw,kw. (18) |
| --- | --- | --- |

| 611 (encephalocele or bifid cranium or cephalocele or cerebellar hernia* or cerebral hernia* or cranial meningoencephalocele or craniocele or cranium bifidum or notoencephalocelecranial or tonsillar hernia*).tw,kw. (8674)  612 (rachischisis or schistorrhachis or status dysraphicus).tw,kw. (493)  613 ((cleft or open) adj1 (spine? or spinal)).tw,kw. (636)  614 (spina? adj (bifida? or bifidum? or dysraphia* or dysraphism*)).tw,kw. (22724)  615 dermal sinus*.tw,kw. (1307)  616 (Arnold-Chiari adj1 (syndrome* or malform* or deform*)).tw,kw. (2304)  617 Chiari malformation?.tw,kw. (6605)  618 (Cantrell* adj1 pentalog*).tw,kw. (173)  619 ((Cantrell Haller Ravitch or thoracoabdominal) adj syndrome*).tw,kw. (21)  620 exp Cardiovascular Abnormalities/ (436109)  621 ((heart or cardiac* or cardio* or aort* or arter* or ventric* or vascular*) adj3 (abnormal* or anomal* or defect* or deform* or malform*)).tw,kw. (291857)  622 ((septa? or septum) adj3 (abnormal* or anomal* or defect* or deform* or malform*)).tw,kw. (69689)  623 Cleft Lip/ (33168)  624 Cleft Palate/ (48907)  625 (cleft? adj1 (lip or lips or palat*)).tw,kw. (53688)  626 (congenital fissur* adj1 (lip or lips or palat*)).tw,kw. (0)  627 ((orofacial or oro facial) adj1 cleft?).tw,kw. (2768)  628 (labioschis* or cheiloschis* or abioschiz*).tw,kw. (73)  629 (harelip? or hare lip?).tw,kw. (1683)  630 (palatischis* or palatoschis* or palatoschiz* or palatum fissu*).tw,kw. (330) | 631 Anorectal Malformations/ (3480)  632 ((anorectal* or ano-rectal*) adj3 (abnormal* or anomal* or atresia* or defect* or deform* or malform* or stenos*)).tw,kw. (6931)  633 ((anus$2 or anal$2) adj3 (abnormal* or anomal* or atresia* or defect* or deform* or malform* or stenos*)).tw,kw. (6453)  634 ((rectum? or rectal$2) adj3 (abnormal* or anomal* or atresia* or defect* or deform* or malform* or stenos*)).tw,kw. (4788)  635 Hydrocephalus/ (66479)  636 hydrocephal*.tw,kw. (72493)  637 (aqueductal stenos* or cerebral ventriculomegal* or Dandy-Walker or Luschka-Magendie foramina atresia* or Hakim$2).tw,kw. (6685)  638 exp Limb Deformities, Congenital/ (76728)  639 ((arm or arms or carpal or carpus or femur* or finger? or foot or feet or fibula? or forearm? or fore arm? or hand or hands or hip or hips or humeral or humerus or knee? or kneecap? or leg or legs or metacarp* or metatars* or patell* or radius or radial or talipedes or talipes or tarsal or tibia? or toe or toes or ulna? or wrist?) adj3 (abnormal* or anomal* or defect* or deform* or malform* or reduc*)).tw,kw. (114261)  640 ((limb or limbs) adj3 (abnormal* or anomal* or defect* or deform* or malform* or reduc*)).tw,kw. (22691)  641 (extremit* adj3 (abnormal* or anomal* or defect* or deform* or malform* or reduc*)).tw,kw. (6470)  642 (clubfoot or club foot or clubfeet or club feet).tw,kw. (8237)  643 (flatfoot or flat foot or flatfeet or flat feet).tw,kw. (5764)  644 (arachnodactyl* or brachydactyl* or ectromelia* or amelia* or hemimelia* or phocomelia* or sirenomelia* or hyperdactyl* or polydactyl* or polysyndactyl* or syndactyl*).tw,kw. (23965)  645 Gastroschisis/ (5218)  646 gastroschis*.tw,kw. (5903) | 647 or/557-646 [OUTCOMES] (7495804)  648 556 and 647 (26984)  649 limit 648 to english [Limit not valid in JBI EBP Database -; records were retained] (24886)  650 (conference abstract or journal conference abstract).pt. (4215749)  651 649 not 650 [CONFERENCE ABSTRACTS REMOVED] (20583)  652 649 use coch [COCHRANE DSR RECORDS] (587)  653 (2019* or 2020* or 2021*).up. (40122041)  654 652 and 653 [COCHRANE DSR UPDATE] (302)  655 651 use cctr [CENTRAL RECORDS] (950)  656 653 and 655 [CENTRAL UPDATE] (778)  657 133 or 274 or 386 or 519 or 654 or 656 [ALL DATABASES - UPDATE PERIOD] (6674)  658 limit 657 to yr="2020-current" (3473)  659 remove duplicates from 658 (2194)  660 657 not 658 (3201)  661 remove duplicates from 660 (2504)  662 659 or 661 [TOTAL UNIQUE RECORDS - UPDATE PERIOD] (4698)  663 662 use medall [MEDLINE UNIQUE RECORDS - UPDATE PERIOD] (2193)  664 662 use emczd [EMBASE UNIQUE RECORDS - UPDATE PERIOD] (1439)  665 662 use jbi [JBI UNIQUE RECORDS - UPDATE PERIOD] (43)  666 662 use coch [COCHRANE DSR UNIQUE RECORDS - UPDATE PERIOD] (302)  667 662 use cctr [CENTRAL UNIQUE RECORDS - UPDATE PERIOD] (683)  668 662 not (663 or 664 or 665 or 666 or 667) [PSYCINFO UNIQUE RECORDS - UPDATE PERIOD] (38) |
| --- | --- | --- |

**Table S1B: Search strategies-CINAHL Translation** Search run on CINAHL (EBSCO) 1982: 19^th^ September 2017; repeated 5^th^ Feb 2019, 2017 to date, 1,036 results; repeated 6^th^ April 2021, 2019 to date, 1,177 results. Interface - EBSCOhost Research Databases, Search Screen - Advanced Search, Database - CINAHL

| **#** | **Query** |
| --- | --- |
| S130 | S37 AND S128 |
| S129 | S37 AND S128 |
| S128 | S38 OR S39 OR S40 OR S41 OR S42 OR S43 OR S44 OR S45 OR S46 OR S47 OR S48 OR S49 OR S50 OR S51 OR S52 OR S53 OR S54 OR S55 OR S56 OR S57 OR S58 OR S59 OR S60 OR S61 OR S62 OR S63 OR S64 OR S65 OR S66 OR S67 OR S68 OR S69 OR S70 OR S71 OR S72 OR S73 OR S74 OR S75 OR S76 OR S77 OR S78 OR S79 OR S80 OR S81 OR S82 OR S83 OR S84 OR S85 OR S86 OR S87 OR S88 OR S89 OR S90 OR S91 OR S92 OR S93 OR S94 OR S95 OR S96 OR S97 OR S98 OR S99 OR S100 OR S101 OR S102 OR S103 OR S104 OR S105 OR S106 OR S107 OR S108 OR S109 OR S110 OR S111 OR S112 OR S113 OR S114 OR S115 OR S116 OR S117 OR S118 OR S119 OR S120 OR S121 OR S122 OR S123 OR S124 OR S125 OR S126 OR S127 |
| S127 | TI gastroschis* or AB gastroschis* |
| S126 | (MH "Gastroschisis") |
| S125 | TI (arachnodactyl* or brachydactyl* or ectromelia* or amelia* or hemimelia* or phocomelia* or sirenomelia* or hyperdactyl* or polydactyl* or polysyndactyl* or syndactyl*) or AB (arachnodactyl* or brachydactyl* or ectromelia* or amelia* or hemimelia* or phocomelia* or sirenomelia* or hyperdactyl* or polydactyl* or polysyndactyl* or syndactyl*) |
| S124 | TI (flatfoot or flat foot or flatfeet or flat feet) or AB (flatfoot or flat foot or flatfeet or flat feet) |
| S123 | TI (clubfoot or club foot or clubfeet or club feet) or AB (clubfoot or club foot or clubfeet or club feet) |
| S122 | TI (extremit* N3 (abnormal* or anomal* or defect* or deform* or malform* or reduc*)) or AB (extremit* N3 (abnormal* or anomal* or defect* or deform* or malform* or reduc*)) |
| S121 | TI ((limb or limbs) N3 (abnormal* or anomal* or defect* or deform* or malform* or reduc*)) or AB ((limb or limbs) N3 (abnormal* or anomal* or defect* or deform* or malform* or reduc*)) |
| S120 | TI ((arm or arms or carpal or carpus or femur* or finger# or foot or feet or fibula# or forearm# or fore arm# or hand or hands or hip or hips or humeral or humerus or knee# or kneecap# or leg or legs or metacarp* or metatars* or patell* or radius or radial or talipedes or talipes or tarsal or tibia# or toe or toes or ulna# or wrist#) N3 (abnormal* or anomal* or defect* or deform* or malform* or reduc*)) or AB ((arm or arms or carpal or carpus or femur* or finger# or foot or feet or fibula# or forearm# or fore arm# or hand or hands or hip or hips or humeral or humerus or knee# or kneecap# or leg or legs or metacarp* or metatars* or patell* or radius or radial or talipedes or talipes or tarsal or tibia# or toe or toes or ulna# or wrist#) N3 (abnormal* or anomal* or defect* or deform* or malform* or reduc*)) |
| S119 | (MH "Limb Deformities, Congenital+") |
| S118 | TI (aqueductal stenos* or cerebral ventriculomegal* or Dandy-Walker or Luschka-Magendie foramina atresia* or Hakim*) or AB (aqueductal stenos* or cerebral ventriculomegal* or Dandy-Walker or Luschka-Magendie foramina atresia* or Hakim*) |
| S117 | TI hydrocephal* or AB hydrocephal* |
| S116 | (MH "Hydrocephalus+") |
| S115 | TI ((rectum# or rectal) N3 (abnormal* or anomal* or atresia* or defect* or deform* or malform* or stenos*)) or AB ((rectum# or rectal) N3 (abnormal* or anomal* or atresia* or defect* or deform* or malform* or stenos*)) |
| S114 | TI ((anus or anal) N3 (abnormal* or anomal* or atresia* or defect* or deform* or malform* or stenos*)) or AB ((anus or anal) N3 (abnormal* or anomal* or atresia* or defect* or deform* or malform* or stenos*)) |
| S113 | TI ((anorectal* or ano-rectal*) N3 (abnormal* or anomal* or atresia* or defect* or deform* or malform* or stenos*)) or AB ((anorectal* or ano-rectal*) N3 (abnormal* or anomal* or atresia* or defect* or deform* or malform* or stenos*)) |
| S112 | (MH "Anorectal Malformations") |
| S111 | TI (palatischis* or palatoschis* or palatoschiz* or palatum fissu*) or AB (palatischis* or palatoschis* or palatoschiz* or palatum fissu*) |
| S110 | TI (harelip# or hare lip#) or AB (harelip# or hare lip#) |
| S109 | TI (labioschis* or cheiloschis* or abioschiz*) or AB (labioschis* or cheiloschis* or abioschiz*) |
| S108 | TI ((orofacial or oro facial) N1 cleft#) or AB ((orofacial or oro facial) N1 cleft#) |
| S107 | TI (congenital fissur* N1 (lip or lips or palat*)) or AB (congenital fissur* N1 (lip or lips or palat*)) |
| S106 | TI (cleft# N1 (lip or lips or palat*)) or AB (cleft# N1 (lip or lips or palat*)) |
| S105 | (MH "Cleft Palate") |
| S104 | (MH "Cleft Lip") |
| S103 | TI ((septa# or septum) N3 (abnormal* or anomal* or defect* or deform* or malform*)) or AB ((septa# or septum) N3 (abnormal* or anomal* or defect* or deform* or malform*)) |
| S102 | TI ((heart or cardiac* or cardio* or aort* or arter* or ventric* or vascular*) N3 (abnormal* or anomal* or defect* or deform* or malform*)) or AB ((heart or cardiac* or cardio* or aort* or arter* or ventric* or vascular*) N3 (abnormal* or anomal* or defect* or deform* or malform*)) |
| S101 | (MH "Cardiovascular Abnormalities+") |
| S100 | TI ((Cantrell Haller Ravitch or thoracoabdominal) N1 syndrome*) or AB ((Cantrell Haller Ravitch or thoracoabdominal) N1 syndrome*) |
| S99 | TI (Cantrell* N1 pentalog*) or AB (Cantrell* N1 pentalog*) |
| S98 | TI Chiari malformation# or AB Chiari malformation# |
| S97 | TI (Arnold-Chiari N1 (syndrome* or malform* or deform*)) or AB (Arnold-Chiari N1 (syndrome* or malform* or deform*)) |
| S96 | TI dermal sinus* or AB dermal sinus* |
| S95 | TI (spina# N1 (bifida# or bifidum# or dysraphia* or dysraphism*)) or AB (spina# N1 (bifida# or bifidum# or dysraphia* or dysraphism*)) |
| S94 | TI ((cleft or open) N1 (spine# or spinal)) or AB ((cleft or open) N1 (spine# or spinal)) |
| S93 | TI (rachischisis or schistorrhachis or status dysraphicus) or AB (rachischisis or schistorrhachis or status dysraphicus) |
| S92 | TI (encephalocele or bifid cranium or cephalocele or cerebellar hernia* or cerebral hernia* or cranial meningoencephalocele or craniocele or cranium bifidum or notoencephalocelecranial or tonsillar hernia*) or AB (encephalocele or bifid cranium or cephalocele or cerebellar hernia* or cerebral hernia* or cranial meningoencephalocele or craniocele or cranium bifidum or notoencephalocelecranial or tonsillar hernia*) |
| S91 | TI (meroanencephal* or holoanencephal*) or AB (meroanencephal* or holoanencephal*) |
| S90 | (TI congenital* or AB congenital* or MW congenital*) and (TI ((absen* or lack* or missing) N2 cranial vault*) or AB ((absen* or lack* or missing) N2 cranial vault*)) |
| S89 | (TI congenital* or AB congenital* or MW congenital*) and (TI ((absen* or lack* or missing) N2 brain*) or AB ((absen* or lack* or missing) N2 brain*)) |
| S88 | TI (anencephal* or aprosencephal*) or AB (anencephal* or aprosencephal*) |
| S87 | TI (acrani* or craniorachischis* or diastematomyeli* or exencephal* or iniencephal* or neurenteric cyst* or neuroenteric cyst* or occult spinal dysraphism* or spinal cord myelodysplasi* or (tethered N2 cord syndrome*)) or AB (acrani* or craniorachischis* or diastematomyeli* or exencephal* or iniencephal* or neurenteric cyst* or neuroenteric cyst* or occult spinal dysraphism* or spinal cord myelodysplasi* or (tethered N2 cord syndrome*)) |
| S86 | TI (neural tube# N2 (defect* or deform* or malform*)) or AB (neural tube# N2 (defect* or deform* or malform*)) |
| S85 | TI (NTD or NTDs) or AB (NTD or NTDs) |
| S84 | (MH "Neural Tube Defects+") |
| S83 | TI ((birth or congenital) N3 (abnormal* or anomal* or defect* or deform*)) or AB ((birth or congenital) N3 (abnormal* or anomal* or defect* or deform*)) |
| S82 | (MH "Abnormalities") OR (MH "Fetal Abnormalities") |
| S81 | TI ("large-for-gestational age" or LGA) or AB ("large-for-gestational age" or LGA) |
| S80 | (MH "Fetal Macrosomia") |
| S79 | TI ("42 0/7" or "42 1/7" or "42 2/7" or "42 3/7" or "42 4/7" or "42 5/7" or "42 6/7" or "42 7/7" or "43 0/7" or "43 1/7" or "43 2/7" or "43 3/7" or "43 4/7" or "43 5/7" or "43 6/7" or "43 7/7") or AB ("42 0/7" or "42 1/7" or "42 2/7" or "42 3/7" or "42 4/7" or "42 5/7" or "42 6/7" or "42 7/7" or "43 0/7" or "43 1/7" or "43 2/7" or "43 3/7" or "43 4/7" or "43 5/7" or "43 6/7" or "43 7/7") |
| S78 | TI (42 week# or 43 week#) or AB (42 week# or 43 week#) |
| S77 | TI (postdate* or post-date* or postmatur* or post-matur* or postterm* or post-term*) or AB (postdate* or post-date* or postmatur* or post-matur* or postterm* or post-term*) |
| S76 | TI (abdom* N3 deliver*) or AB (abdom* N3 deliver*) |
| S75 | TI (C-section* or Csection*) or AB (C-section* or Csection*) |
| S74 | TI (cesarean* or caesarean*) or AB (cesarean* or caesarean*) |
| S73 | (MH "Cesarean Section+") |
| S72 | TI (preterm or prematur*) or AB (preterm or prematur*) |
| S71 | (MH "Childbirth, Premature") |
| S70 | TI (FGR or IUGR) or AB (FGR or IUGR) |
| S69 | TI ((f#etal or f#etus* or intrauterin*) N1 grow* N3 (restrict* or retard*)) or AB ((f#etal or f#etus* or intrauterin*) N1 grow* N3 (restrict* or retard*)) |
| S68 | (MH "Fetal Growth Retardation") |
| S67 | TI (placenta* N2 mediate*) or AB (placenta* N2 mediate*) |
| S66 | TI (placenta* N3 (abnormal* or disease* or disorder* or dysfunction*)) or AB (placenta* N3 (abnormal* or disease* or disorder* or dysfunction*)) |
| S65 | (MH "Placental Insufficiency") |
| S64 | (MH "Placenta Diseases+") |
| S63 | TI (DVT or thromboemboli* or VTE) or AB (DVT or thromboemboli* or VTE) |
| S62 | (MH "Venous Thromboembolism") |
| S61 | TI macrosomi* or AB macrosomi* |
| S60 | TI PID or AB PID |
| S59 | TI ((diabet* or DM or T2DM) N3 (gestational* or maternal* or "new onset" or pregnancy-induced)) or AB ((diabet* or DM or T2DM) N3 (gestational* or maternal* or "new onset" or pregnancy-induced)) |
| S58 | (MH "Diabetes Mellitus, Gestational") |
| S57 | TI HELLP or AB HELLP |
| S56 | TI PIH or AB PIH |
| S55 | TI ((eclamp* or hypertensi* or preeclamp* or pre-eclamp* or toxemi* or toxaemi* or EPH or hemolys#s) N3 (gestational* or maternal* or "new onset" or pregnancy-induced)) or AB ((eclamp* or hypertensi* or preeclamp* or pre-eclamp* or toxemi* or toxaemi* or EPH or hemolys#s) N3 (gestational* or maternal* or "new onset" or pregnancy-induced)) |
| S54 | (MH "Pregnancy-Induced Hypertension+") |
| S53 | TI ((admit* or admission* or readmit* or readmission*) N3 (hospital# or critical care or intensive care or ICU or ICUs or NICU or NICUs or SICU or SICUs)) or AB ((admit* or admission* or readmit* or readmission*) N3 (hospital# or critical care or intensive care or ICU or ICUs or NICU or NICUs or SICU or SICUs)) |
| S52 | TI hospitali* or AB hospitali* |
| S51 | (MH "Hospitalization+") |
| S50 | TI ((abort* N2 spontaneous*) or miscarriage# or (recur* N2 loss*) or (habit* N2 abort*)) OR AB ((abort* N2 spontaneous*) or miscarriage# or (recur* N2 loss*) or (habit* N2 abort*)) |
| S49 | (MH "Abortion, Spontaneous+") |
| S48 | TI (stillbirth* or stillborn*) OR AB (stillbirth* or stillborn*) |
| S47 | TI ((baby or babies or f#etal or f#etus* or neonat* or newborn*) N3 (dead or demise# or died or dying)) OR AB ((baby or babies or f#etal or f#etus* or neonat* or newborn*) N3 (dead or demise# or died or dying)) |
| S46 | (MH "Perinatal Death") |
| S45 | TI (mortalit* or death* or fatal*) OR AB (mortalit* or death* or fatal*) |
| S44 | (MH "Infant Mortality") |
| S43 | (MH "Infant Death+") |
| S42 | (MH "Maternal Mortality") |
| S41 | (MH "Pregnancy Complications/MO") |
| S40 | TI ((maternal* or mother* or baby or babies or f#etal* or f#etus* or neonat* or newborn*) N3 outcome*) OR AB ((maternal* or mother* or baby or babies or f#etal* or f#etus* or neonat* or newborn*) N3 outcome*) |
| S39 | TI ((pregnan* or prenatal* or antenatal* or ante natal* or antepartum or ante partum or perinatal* or peripartum) N3 outcome*) OR AB ((pregnan* or prenatal* or antenatal* or ante natal* or antepartum or ante partum or perinatal* or peripartum) N3 outcome*) |
| S38 | (MH "Pregnancy Outcomes") |
| S37 | S27 AND S36 |
| S36 | S28 OR S29 OR S30 OR S31 OR S32 OR S33 OR S34 OR S35 |
| S35 | (MH "Pregnancy Complications/EP") |
| S34 | TI prevalen* OR AB prevalen* |
| S33 | (MH "Prevalence") |
| S32 | TI (logit* N1 model*) OR AB (logit* N1 model*) |
| S31 | TI logistic* OR AB logistic* |
| S30 | TI predict* OR AB predict* |
| S29 | TI (risk or risked or risks or risky or risking or risk-related) OR AB (risk or risked or risks or risky or risking or risk-related) |
| S28 | (MH "Perinatal Risk (Saba CCC)") OR (MH "Infant, High Risk") |
| S27 | S24 not (S25 or S26) |
| S26 | (PT "letter") or (PT "letter to the editor") |
| S25 | (PT "book review") or (PT "commentary") or (PT "editorial") or (PT "newspaper") |
| S24 | S22 not S23 |
| S23 | (MH "Animals+") not ((MH "Animals+)" and (MH "Humans")) |
| S22 | S8 AND S21 |
| S21 | S9 OR S10 OR S11 OR S12 OR S13 OR S14 OR S15 OR S16 OR S17 OR S18 OR S19 OR S20 |
| S20 | TI (waisthip or waistheight or waistthigh or WHR or WHtR or WTR) OR AB (waisthip or waistheight or waistthigh or WHR or WHtR or WTR) |
| S19 | TI (waist* N2 (circumference* or hip* or height or thigh*)) OR AB (waist* N2 (circumference* or hip* or height or thigh*)) |
| S18 | TI (anthropometr* or BMI or BMIs or skinfold* or SFT or SFTs) OR AB (anthropometr* or BMI or BMIs or skinfold* or SFT or SFTs) |
| S17 | (MH "Skinfold Thickness") |
| S16 | (MH "Waist Circumference") or (MH "Waist-Hip Ratio") or (MH "Abdominal Fat") |
| S15 | (MH "Anthropometry") |
| S14 | TI ((abdominal* or central* or subcutaneous*) N2 (obes* or fat* or adipos*)) OR AB ((abdominal* or central* or subcutaneous*) N2 (obes* or fat* or adipos*)) |
| S13 | (MH "Adipose Tissue Distribution") |
| S12 | (MH "Adipose Tissue+") |
| S11 | TI (body N2 (size* or shape* or weight* or fat* or silhouette*)) OR AB (body N2 (size* or shape* or weight* or fat* or silhouette*)) |
| S10 | TI (obesity* or obese or overweight or over weight or adiposit*) OR AB (obesity* or obese or overweight or over weight or adiposit*) |
| S9 | (MH "Obesity+") |
| S8 | S1 OR S2 OR S3 OR S4 OR S5 OR S6 OR S7 |
| S7 | TI (prenatal* or antenatal* or ante natal* or antepartum or ante partum) OR AB (prenatal* or antenatal* or ante natal* or antepartum or ante partum) |
| S6 | (MH "Prenatal Care") |
| S5 | TI pregnan* OR AB pregnan* |
| S4 | (MH "Pregnancy Trimesters+") |
| S3 | (MH "Expectant Mothers") |
| S2 | (MH "Pregnancy Complications+") |
| S1 | (MH "Pregnancy") |

**Table S2: Contacting authors for additional information**

| **Citation** | **Reason for contact** | **Author response** | **Data provided** |
| --- | --- | --- | --- |
| Oriji et al. 2017 ^29^ | Not clear when anthropometry measurements were taken | Data provided  (Dr John Dimkpa Ojule) | 15 - 18 weeks |
| Balani et al. 2018 ^8^ | The accessible paper was a submitted for review. I sought clarification of any changes after publication. | No response | We reviewed the abstract that shows some different figures to what is reported in the paper so it has been updated accordingly. |
| Ebrahimi-Mameghani et al. 2013 ^30^ | One of the odds ratios reported is very different from all the others (OR for WC <80cm vs 80-88cm = 0.39 (0.04, 3.38)) – authors contacted to check that figures are accurate | Data provided  (Professor Mehrangiz Ebrahimi-Mameghani) | Figures are accurate |
| Zhu et al. 2019 ^2^ | The number of women in each quartile for waist circumference, waist-hip-ratio, and the number of women who had gestational diabetes in each of these quartiles to calculate odds ratios | Data provided  (Dr Yeyi Zhu) | \| N \| All \| GDM (Y) \| \| --- \| --- \| --- \| \| Waist circumference \| \| \| \| Q1 \| 434 \| 16 \| \| Q2 \| 440 \| 33 \| \| Q3 \| 436 \| 47 \| \| Q4 \| 440 \| 90 \| \| Waist to hip ratio \| \| \| \| Q1 \| 433 \| 11 \| \| Q2 \| 433 \| 30 \| \| Q3 \| 444 \| 51 \| \| Q4 \| 440 \| 94 \| |
| Han et al. 2018 ^31^ | The number of women in each comparison group for waist circumference (<78.5cm, 78.5 to <85cm and ≥85cm). The number of women who had gestational diabetes in each of these groups. | No response |  |
| White et al. 2016 ^11^ | Unit of increase for the odds ratios reported not defined in paper. To clarify what variables were included in models 5 & 6. | Information provided  (Professor Dharmintra Pasupathy, Dr Sara Louise White) | Per 1cm increase |
| He et al. 2017 ^22^ | Study reports on associations between GDM and waist circumference as a dichotomous variable, but the paper does not define the waist circumference measurements used. Same for neck circumference | No response | No information |
| Mendoza et al. 2018 ^32^ | Unit of increase for the odds ratios reported not defined in paper | Information provided  (Dr Rosa Corcoy) | Per 1cm increase |
| Egan et al. 2017 ^20^ | Unit of increase for the odds ratios reported not defined in paper | Information provided  (Dr. Aoife Egan) | Per 1cm increase |
| Gagliardi et al. 2016 ^33^ | Unit of increase for the OR reported not defined in the table | No response | No information |

**Table S3. Characteristics of the included studies**

| **Author, year and country** | **Study design and sample size** | **Study period** | **Inclusion criteria** | **Exclusion criteria** | **Maternal adiposity exposures reported** | **Gestation at adiposity measurement** | **Maternal outcomes reported** |
| --- | --- | --- | --- | --- | --- | --- | --- |
| Alptekin et al. 2016; Turkey ^7^ | Cohort; n=227 | December 2014–May 2015 | Pregnant women, at 7-12 weeks’ gestation | T1/2DM, fasting plasma glucose >95mg/dL, multiple pregnancy, untreated endocrine disturbances, chronic hypertension, PE, medication affecting fasting glucose / insulin | Waist circumference; Hip circumference; Waist hip ratio | 7–12 weeks | GDM |
| Alvarado et al; 2021; USA ^15^ | Cohort; n=46 | Not reported | Healthy, non-smokers, no pre-existing cardio metabolic disorders (hypertension, diabetes, thyroid disorders) | Not reported | Fat mass; Body fat %; Fat-free mass | 12-14 weeks | GDM |
| Alves et al. 2020; Brazil ^34^ | Cohort; n=518 | 2016-2018 | Singleton at <20 weeks’ gestation | History of GDM or pre-GDM | Visceral adipose tissue depth | 14 weeks | GDM |
| Bai et al. 2020; Australia ^35^ | Cohort; n=118 | Not reported | Singleton, ≤13 weeks’ gestation, ≥18 years, capable of giving informed consent. | Interpreter services, planning home birth, history of hypertension/GDM, chronic hypertension, existing DM and/or BMI≥30. | Body fat percentage | ≤13 weeks | GDM |
| Balani et al. 2014; UK ^8^ | Case control; n=302 | Not reported | Pregnant women, obese, no DM | T1/2DM, fasting plasma glucose >95mg/dL, multiple pregnancy, untreated endocrine disturbances, chronic hypertension, PE, medication affecting fasting glucose / insulin | Waist hip ratio; Total body fat percentage; Visceral fat mass | 14–17 weeks | GDM |
| Bartha et al. 2007; Spain ^36^ | Cohort; n=30 | Not reported | Singleton, 11-14 weeks’ gestation | Multiple pregnancy, maternal conditions (e.g., ovarian cysts) | Visceral fat thickness; Subcutaneous fat thickness; Ratio of visceral to subcutaneous fat | 11-14 weeks | Glycemia; Insulinemia; HOMA; Systolic/diastolic BP; Total cholesterol; HDL-C; LDL-C; triglycerides; cholesterol/HDL-C; Free fatty acids |
| Basraon et al. 2016; USA ^1^ | Trial cohort; n=2300 | 2003-2008 | Singleton, nulliparous, no history hypertension, substance abuse, fetal abnormalities, uterine bleeding, IVF, proteinuria, diabetes, other medical problems | Not reported | Waist hip ratio | 9–16 weeks | GDM |
| Bourdages et al 2018; Canada ^37^ | Cohort; n=1048 | March 2011 –December 2014 | Viable singleton, 11-14 weeks’ gestation, healthy nulliparous, aged ≥18 years | Chronic hypertension, history of DM, chromosomal anomaly, lethal malformation | Subcutaneous; Total; Visceral adipose tissue thickness | 11 -14 weeks | GDM |
| Brisson et al. 2013; Canada ^38^ | Cohort; n=180 | October 2006 – December 2010 | Singleton, white women of French-Canadian origin, at the beginning of routine prenatal care at the Chicoutimi Hospital | >40 years, T1/2 DM, disorders affect glucose metabolism, alcohol /drug abuse | Waist circumference | 11-14 weeks | HOMA-IR |
| Campbell et al. 2012; Australia ^39^ | Cohort; n=956 | 1998–2000 | Child-bearing age, pregnant after health check, gave birth, complete record | Pregnant at health check, ectopic pregnancy, miscarriage, unknown outcome, no perinatal record | Waist circumference | Not reported | GDM |
| Catov et al. 2020; USA ^40^ | Cohort; n=1302 | 2015–2016 | No DM at enrolment and before pregnancy, gave birth at least once after baseline | Multiple gestation, incomplete delivery date information | Waist circumference | Not reported | GDM |
| Dakshnamurthy et al. 2017; India ^41^ | Case control; n=60 | Not reported | Pregnant women, (cases) newly diagnosed with GDM in their 2^nd^ trimester | Multiple gestations, controls with risk factors for GDM (e.g. obesity, family history DM) | Waist circumference; Visceral Adiposity Index | Mean wks:  19.70 ±3.13  20.27±2.84 | GDM |
| De Souza et al. 2014; Canada ^42^ | Cohort; n=79 | Not reported | Viable singleton, aged ≥18, healthy | Pre-pregnancy T2 DM or prior GDM | Subcutaneous; Visceral; Total adipose tissue depth | 11-14 weeks | HOMA-IR; Insulin sensitivity index |
| De Souza et al. 2016; Canada ^43^ | Cohort; n=485 | 2012–2014 | Viable singleton, aged ≥18, healthy | Pre-pregnancy or prior GDM | Subcutaneous; Visceral; Total adipose tissue depth | 11-14 weeks | GDM |
| De Souza et al. 2016; Canada ^44^ | Cohort; n=476 | Not reported | Viable singleton, aged ≥18, healthy | DM, prior GDM, PCOS, Metformin, infertility treatment, corticosteroids/ other medications, chronic or pregnancy disorder that may affect liver function (e.g. viral hepatitis) | Hepatic fat+visceral fat; Hepatic fat+total adipose tissue | 11–14 weeks | GDM composite outcome (impaired fasting glucose, gestational impaired glucose tolerance or GDM) |
| Ebrahimi-Mameghani et al. 2013; Iran ^30^ | Cohort; n=948 | 2009–2010 | Nulliparous | Disproportionate GWG for BMI without PE/GDM, incomplete delivery file, history of hyperemesis, recurrent spontaneous abortion, uterine surgery, molar pregnancies, any chronic diseases or special diet | Waist circumference | 1^st^ trimester | GDM; Gestational hypertension; Preeclampsia |
| Egan et al. 2017; 11 European centers, 9 countries ^20^ | Trial cohort; n=1023 | January 2012 – February 2014 | Singleton, ≤19+6 weeks gestation, aged ≥18 years, pre-pregnancy BMI≥29 kg/m^2^ , ability to give informed consent | Pre-existing DM, need for complex diet, inability to walk ≥100m safely, any chronic medical condition or psychiatric disease | Waist circumference; Neck circumference | ≤19+6 weeks | GDM |
| Endeshaw et al. 2016; Ethiopia ^45^ | Case control; n=453 | June – September 2014 | Attending antenatal Care/ seeking skilled delivery service during the study period | Not reported | Mid-upper-arm circumference | 1^st^ trimester | Preeclampsia/ eclampsia |
| Gagliardi et al. 2016; Italy ^33^ | Case control; n=150 | January 2014 – February 2016 | Singleton, nulliparous, normal BP at enrolment, certain date of pregnancy, normal fetal parameters, absence of maternal disease, BMI<30 kg/m^2^ | Undetermined gestational age, tobacco use, multiple pregnancy, chronic maternal disease, medication use, reproductive techniques | Bio-electrical impedance fat mass | 11 –13+6 weeks | Complicated pregnancy (pre-eclampsia and/or fetal growth restriction or combined) |
| Gao et al. 2017; China ^46^ | Cohort; n=919 | January – December 2015 | Live-birth, no mental illnesses/brain diseases, permanent residents in the Kaifu District, health care records registered in CHMIS | Multiple births, antenatal care initiated later than 12 weeks’ gestation, incomplete data | Waist circumference; combined BMI and waist circumference | <12 weeks | GDM; Caesarean;  Composite outcome (one or more adverse pregnancy outcomes) |
| Gur et al. 2014; Turkey ^47^ | Cohort; n=94 | January 2012 – January 2013 | Singleton, 18–40 years | T1/2 DM, hypertension, metabolic disease, chronic drug therapy | Waist circumference; Visceral fat; Subcutaneous fat | 4–14 weeks | GDM; BP; Triglycerides; HDL-C, LDL-C; Metabolic syndrome; Total cholesterol |
| Han et al. 2017; China ^31^ | Cohort; n=17803 | October 2010 –August 2012 | Had GCT at 24–28 weeks’ gestation | Positive GCT but did not undergo OGTT, pre-existing DM | Waist circumference | <12 weeks | GDM |
| Hancerliogullari et al. 2020; Turkey ^21^ | Cohort; n=525 | 2019 | Healthy pregnant women 11-14 weeks’ gestation, admitted to the antenatal outpatient clinic | <18 or >45 years, multiple pregnancy, hypertension, kidney, liver, thyroid, other endocrine diseases, pre-diabetes. | Waist circumference; Neck circumference | 11-14 weeks | GDM |
| Harville et al. 2014; Finland ^48^ | Cohort; n=349 | 1983-2007 | Singleton, Finnish National Birth registry, in Cardiovascular Risk in Young Finns Study | T1DM | Waist circumference | Pre-pregnancy | GDM |
| He et al. 2017; China ^22^ | Nested case control; n=255 | October –December 2014 | 18–35 years, 16 weeks' gestation | Not reported | Waist circumference; Neck circumference | 16 weeks | GDM |
| Ianniello et al. 2013; Italy ^49^ | Cohort; n=32 | Not reported | BMI>25 kg/m^2^, high risk for GDM (according to the American Diabetes Association), | Hypertension, T2DM, medications affecting glycaemic/lipid metabolism, smoke during pregnancy. | Fat mass;  Lean mass | 8-11 weeks | GDM |
| Iqbal et al. 2007; Pakistan ^16^ | Cohort; n=750 | October 2002 – May 2004 | ≤18 weeks' gestation, South Asian origin | Existing DM | Body fat %; Fat-free mass; Fat mass | <18 weeks | GDM |
| Kausar et al. 2013; India ^27^ | Cohort; n=150 | Aug 2010 -July 2012 | Singleton, 6-16 weeks’ gestation, systolic BP<140 mm Hg, diastolic BP <90 mm Hg, follow up and deliver in Bharati Hospital | Smokers, multiple/molar pregnancy, chronic diseases (hypertension, DM, severe anaemia, heart disease, renal disease), anti-Phospholipid syndrome, any congenital anomaly | Waist circumference; Waist hip ratio;  Waist height ratio | 6-16 weeks | Gestational hypertension; Preeclampsia |
| Kennedy et al. 2016; Australia ^13^ | Cohort; n=1461 | 2012-2014 | Pregnant women | Multiple pregnancy, insulin-dependent DM, miscarriage, termination, major anomalies | Abdominal subcutaneous fat thickness | 11–14 weeks | GDM; Assisted delivery  Caesarean; Hypertensive disease; Induction of labour |
| Khare et al. 2017; India ^3^ | Cohort; n=120 | January – December 2014 | Pregnant women <16 weeks’ gestation | DM, hypertension, cardiac disease, familial dyslipidaemias, or hypothyroidism | Waist hip ratio | <16 weeks | GDM; hypertension/ preeclampsia; Serum lipids; Total Antenatal Complications |
| KhushBakht et al. 2018; Pakistan ^50^ | Cohort; n=90 | July 2017–March 2018 | Women who consented to participate in the study | Not reported | Neck circumference | 16 weeks | GDM; Triglycerides; Total cholesterol |
| Lacroix et al. 2013; Canada ^17^ | Cohort; n=445 | Not stated | Singleton, 18-40 years; planning to deliver at the Centre Hospitalier Universitaire de Sherbrooke | Prior GDM, drug/alcohol abuse, uncontrolled endocrine disease, renal failure, major medical condition affecting glucose regulation | Waist circumference; Body fat percentage | 1^st^ trimester | GDM |
| Lacroix et al. 2016; Canada ^51^ | Cohort; n=675 | January 2010 – July 2013 | 1st trimester blood sample visit, planning to deliver at the Centre Hospitalier Universitaire de Sherbrooke | <18 years, multiple pregnancy, pre-GDM; drugs/ alcohol abuse, uncontrolled endocrine disease, other major medical conditions | Body fat percentage | 5–16 weeks | Gestational weight gain |
| Li et al. 2018; China ^52^ | Cohort; n=371 | April 2016–April 2017 | Singleton, have prenatal care services and delivery at the study site. | Thyroid disease or operations | Neck circumference | 11-13+6 weeks | GDM |
| Lima et al. 2019; Brazil ^53^ | Cohort; n=200 | 2014-2015 | ≤16 weeks’ gestation | Multiple pregnancy, pre-GDM, psychiatric disorders, chronic maternal diseases (hypertension, heart disease, kidney disease, epilepsy, kidney failure), congenital malformations | Waist circumference;  Subcutaneous fat; Visceral fat; Arm circumference; Leg circumference; Skinfold (tricep, suprailiac) | 16 weeks | Metabolic syndrome |
| Liu et al. 2020; China ^18^ | Cohort; n=1318 | 2018 | Healthy, low-risk pregnancies delivered in Guiyang Maternal and Child Health Care Hospital | non-singleton pregnancy, OGTT records not available | Fat mass index; Fat mass %; Skeletal muscle mass % | 13 weeks | GDM |
| Madhavan et al. 2008; India ^4^ | Cohort; n=106 | April 2005–April 2006 | 18-35 years, ≤12 weeks’ gestation at first antenatal visit, singleton, live intrauterine pregnancies | History of diabetes, thyroid, pituitary disorders, comorbid conditions/ severe systemic illness; metabolic/ inherited disorders, drugs known to cause insulin resistance. | Waist circumference;  Waist Hip ratio | ≤12 weeks | GDM; Pregnancy-induced hypertension; Abnormal deliveries; Induction |
| Maitland, et al. 2014; UK ^23^ | Trial cohort; n=117 | March 2009–May 2011 | Singleton, 15+0- 18+6 weeks’ gestation, BMI≥30 kg/m^2^ at 1^st^ antenatal visit | Hypertension, renal disease, lupus, antiphospholipid syndrome, sickle cell disease, thalassemia, coeliac disease, thyroid disease, current psychosis, metformin, unable or unwilling to give informed consent | Waist circumference;  Hip circumference | 16-18+6 weeks gestation | GDM |
| Martin et al. 2009; Canada ^54^ | Cohort; n=58 | January–May 2008 | Singleton eligible for the study at 11–14 weeks’ gestation | Women with T1/2 DM pre-pregnancy, history of GDM | Subcutaneous; Visceral adipose tissue | 11–14 weeks | Abnormal glucose challenge test |
| McDonnold et al. 2016; USA ^55^ | Cohort; n=2276 | July 2003 –February 2008 | Low risk nulliparous women, data to determine waist-hip ratio and BMI at enrolment | Congenital anomalies and intrauterine fetal demise | Waist Hip ratio;  Combined waist hip ratio and BMI | 9-16 weeks | Caesarean |
| Mendoza et al. 2018; 11 sites in 9 European countries ^32^ | Trial cohort; n=971 | Not reported | Singleton, ≤19 weeks +6 days’ gestation, >18 years, with OGTT, pre-pregnancy BMI ≥29kg/m^2^ | Cannot walk 100m safely, complex diet requirements, chronic medical/ psychiatric conditions. | Neck circumference | ≤ 19 weeks+6 days | GDM |
| Migda et al. 2016; Poland ^56^ | Cohort; n=157 | 2011-2013 | Singleton, Caucasian, early symptoms of metabolic syndrome | Not reported | Waist circumference; Waist Hip ratio | 11-13+6 weeks | GDM; Caesarean |
| Minooee et al. 2017; Iran ^9^ | Cohort; n=2943 | 1998, follow-up phases at 3-year intervals ^a^ | 20–50 years, with at least one term pregnancy at the beginning of the study | Overt diabetes, impaired fasting glucose (IFG), Impaired glucose tolerance (IGT), simultaneous IGT and IFG, missing data | Waist circumference;  Hip circumference;  Waist Hip ratio | Pre-pregnancy | GDM |
| Mostafavi et al. 2015; Iran ^57^ | Cohort; n=80 | Not reported | Pregnant women at 12 weeks’ gestation, 18-40 years, non-smokers | Known cardiac/renal/liver disease, PCOS, hypo/hyperthyroidism, autoimmune disease, hospital admission last 6 months, previous abortion, fasting glucose>1.1g/L, systolic BP>130mmHg, diastolic BP >85mmHg, triglycerides>1.5g/L, HDL-C<0.5g/L, | Waist circumference | 12 weeks | HOMA-IR; insulin resistance;  Triglycerides |
| Oriji et al. 2017; Nigeria ^29^ | Cohort; n=250 | April–May 2015 | Singleton, 15-18 weeks’ gestation at booking, giving consent | HIV, pre-GDM, acute or chronic illness at booking, uncertain date | Waist circumference; Abdominal skinfold thickness | 15–18 weeks | GDM |
| Piuri, et al. 2016; Italy ^58^ | Cohort; n=126 | June 2012–June 2014 | 1^st^ trimester, attending the outpatient clinic for nuchal translucency and combined test | Twin pregnancy, pre-existing maternal disease (e.g. diabetes, hypertensive or renal disease) | Waist circumference; Skinfold thickness (subscapular, bicep, triceps); Fat mass; Fat-free mass; Total body water | 12–14 weeks | Hypertensive appropriate gest age (HDP-AGA) |
| Pontual et al. 2016; Brazil ^59^ | Cohort; n=334 | Not reported | Viable singleton at 15–20 weeks’ gestation, aged 15–49 years | Previous diagnosis of DM (type I or II) or dyslipidaemia | Visceral adipose tissue depth | 15–20 weeks | Fasting HOMA-IR, insulin and glucose; 1hr/2hr glucose; VLDL-cholesterol; Triglycerides |
| Popova et al. 2015; Russia ^60^ | Case control; n=548 | May 2011 -December 2012 | OGTT at 24-28 weeks’ gestation, available medical records, pregnancy <13 weeks. | T1/2DM, other diseases/drugs affect carbohydrate metabolism within 6 months, alcohol/drugs during pregnancy, refuse to participate | Abdominal circumference | <13 weeks | GDM |
| Ray et al. 2017; Canada ^61^ | Cohort; n=463 | August 2012–May 2015 | Viable singleton, no DM within or outside pregnancy | Not reported | Visceral adipose tissue depth | 11-14 weeks | Preeclampsia; Preeclampsia with preterm birth >37 weeks’ |
| Salem et al. 2012; UK (ALSPAC) ^62^ | Cohort; n=3083 | April 1991- December 1992 | Nulliparous, singleton live born infant, labour at term, documented waist-hip ratio and infant birthweight | Not reported | Waist Hip ratio | Pre-pregnancy | Non-spontaneous delivery |
| Sattar et al. 2001; UK ^63^ | Cohort; n=1142 | Not specified | Singleton, 25–32 years, attended 1^st^ antenatal visits. | Pre-existing hypertension, diastolic BPs >90 mmHg | Waist circumference | 6-16 weeks | Preeclampsia; Pregnancy-induced hypertension |
| Sharadha et al. 2016; India ^64^ | Cohort; n=239 | March 2013–February 2014 | Singleton, antenatal visit within 10 weeks of pregnancy, delivery at study hospital | BMI<18 kg/m^2^, hyperemesis, diabetes, hypertension, thyroid disorders, epilepsy, bronchial asthma, congenital heart disease, chronic kidney disease, connective tissue disorders | Waist circumference | ± 10 weeks | GDM; Preeclampsia; Gestational hypertension |
| Sina et al. 2014; Australia ^28^ | Nested case control; n=168 | 1992-2012 | Not reported | Pregnant before the baseline measurements | Waist circumference;  Hip circumference;  Waist Hip ratio;  Waist Height ratio | Pre-pregnancy | Gestational hypertensive disorders |
| Sina et al. 2015; Australia ^10^ | Nested case control; n=131 | 1992-2012 | Members of a remote Aboriginal community in the Northern Territory, aged 5+ years who participated in one or two community-wide health screenings performed in 1992—1996 and in2004—2006 | Menstruating females, and people who were hospitalised or on dialysis | Waist circumference; Hip circumference; Waist Hip ratio; Waist Height ratio | pre-pregnancy | GDM |
| Suresh et al. 2012; Australia ^14^ | Cohort; n=1200 | 2006-2010 | Nulliparous, ultrasound images sufficient quality for SFT measurement at 18–22 weeks’ | Missing/incomplete BMI or outcome data, smoker, drug user | Subcutaneous fat tissue | 18-22 weeks | GDM; Caesarean; Cumulative adverse pregnancy outcomes ^b^ |
| Sween et al. 2015; USA ^26^ | Cohort; n=373 | Not reported | Singleton, complete early pregnancy (1^st^ trimester) data | Pre-existing hypertension, DM, renal disease, other medical complications | Waist circumference; Waist Hip ratio; Body fat | ±10 weeks | Preeclampsia |
| Taebi et al. 2015; Iran ^25^ | Cohort; n=1000 | April 2008 to May 2010 | ≤12 weeks’ gestation at 1^st^ antenatal visit, no history of systemic disease, hypertension, DM, previous PE | Termination of pregnancy before 24 weeks’ gestation | Waist circumference;  Hip circumference;  Waist Hip ratio | ≤12 weeks | Preeclampsia |
| Thaware et al. 2019; UK ^12^ | Cohort; n=80 | Not reported | Singleton, aged 18–40 years | Pre-GDM or a history of GDM | Depth subcutaneous; Visceral adipose tissue | 9-18 weeks | GDM |
| Tomedi et al. 2014; USA ^65^ | Cohort; n=413 | Not reported | Pregnant women at ≤13 weeks’ gestation | Abortion, implausible or missing weight measurements, no weight within 30 days of GDM screening, missing data, glucose screening performed ≤24 weeks’ gestation | Waist circumference;  Skinfold (biceps and triceps) | ≤ 13 weeks | Maternal post load glucose concentration |
| Vieira et al. 2017; Australia, New Zealand, UK and Ireland ^66^ | Cohort; n=5592 | November 2004- February 2011 | Nulliparous with singleton, healthy | High risk of preeclampsia/SGA/ preterm birth, anomaly/abnormal karyotype fetus, interventions that may modify pregnancy outcome | Waist circumference;  Arm circumference;  Hip circumference | 14–16 weeks | Preeclampsia |
| Wang et al. 2019; China ^5^ | Case control; n=2698 | 2016-2018 | Singleton, ≥18 years | Previous GDM/DM, diseases affecting glucose metabolism (e.g. chronic hypertension, thyroid), abortion or induced labour, family history of DM, incomplete data | Waist Hip ratio;  Arm circumference;  Body fat %; Fat-free mass; Fat mass index;  FM/FFM ratio | 13-20 weeks | GDM |
| Wang, et al. 2015; China ^67^ | Nested case control; n=210 | July 2007 to August 2011 | Women who met all the strict definitions described in the exclusion criteria | Multiple gestation, birth defects, pre- hypertension, kidney disease, DM, other chronic medical diseases | Body fat %; Fat-free mass; Fat-free mass index | 12–16 weeks | Preeclampsia |
| Wen et al. 2017; China ^68^ | Cohort; n=1685 | February 2009 and December 2012 | Women who planned to have baby in the next 6 months | Pregnant at baseline assessment | Waist circumference | Pre-pregnancy | New onset of hypertension |
| White et al. 2016; UK ^11^ | Trial cohort; n=1303 | 2009–2014 | Singleton, >16 years, BMI ≥30kg/m^2^, available OGTT data. | Not reported | Waist circumference; Neck Circumference; Mid arm circumference; Hip circumference; Thigh circumference; Wrist circumference; Waist Hip ratio; Neck Thigh ratio; Waist Thigh ratio; Waist Height ratio; Skinfolds (triceps, biceps, subscapular, suprailiac, sum of skinfolds) | 15+0 to 18+6 weeks | GDM |
| Yamamoto et al. 2001; Japan ^24^ | Case control; n=224 | January –December 1999 | Not reported | Initially visited at >9 weeks’ gestation, cardiovascular diseases, hypertension, DM before pregnancy | Waist Hip ratio | < 9 weeks | Preeclampsia |
| Yang et al. 2017; South Korea ^69^ | Cohort; n=333 | February 2015 - June 2016 | Singleton, 1^st^ trimester, 19- 41 years | T1/2DM, history of smoking, and/or other medical conditions (e.g. high BP, metabolic syndrome) | Abdominal subcutaneous fat thickness | 10+6 to 13+6 weeks | GDM |
| Yeboah et al. 2017; Ghana ^70^ | Cohort; n=314 | Not reported | Attending 1^st^ routine antenatal visit at Volta regional hospital | Not reported | Body fat %; Muscle mass %; Water mass % | 11.7 +/- 0.8 weeks | Preeclampsia |
| Zhang et al. 1995; USA ^6^ | Cohort; n=720 | 1986-1993 | Not reported | Multiple pregnancy, history of DM/GDM at baseline, a pregnancy of <25 weeks, missing GDM status. | Waist: Hip ratio | Pre-pregnancy | GDM |
| Zhang et al. 2020; China ^19^ | Cohort; n=22223 | 2014-2015 | Singleton, underwent bioelectrical impedance analysis between 1 January 2014 and 30 December 2015 | Pre-existing DM | Fat mass; Fat-free mass; Body fat %; Visceral fat; Lean trunk mass; Lean right/left arm mass; Lean right/left leg mass | <17 weeks | GDM |
| Zhu et al. 2019; USA ^2^ | Cohort; n=1750 | Not reported | 18-45 years, <11 weeks’ gestation, all races and ethnicities | Multiple pregnancy, cancer, Hepatitis C, liver cirrhosis, termination of pregnancy, DM or GDM, use of DM medication before clinic examination | Waist circumference;  Waist Hip ratio | 10-13 weeks | GDM |

Footnote:

Abbreviations: DM- Diabetes Mellitus; GDM – gestational DM; LGA – large for gestational age; SGA – small for gestational age; IADPSG – The International Association of Diabetes and Pregnancy Study Groups; WHO – World Health Organisation; HOMA-IR- Homeostatic Model Assessment of Insulin Resistance; HDL-C- high-density lipoprotein cholesterol, LDL-C: low-density lipoprotein cholesterol; VLDL-C: very low-density lipoprotein cholesterol; GWG-gestational weight gain; OGTT- oral glucose tolerance test; GA- gestational Age; SFT- subcutaneous fat thickness, IFG- impaired fasting glucose; IGT- impaired glucose tolerance; PCOS-Polycystic ovary syndrome.

a – phase 1: 1999–2001, phase 2: 2002–2005, phase 3: 2005–2008, phase 4: 2008–2011 and phase 5: 2011–201

b- Cumulative adverse pregnancy outcomes includes gestational diabetes, pre-eclampsia, gestational hypertension, instrumental deliveries, caesarean deliveries, preterm birth, macrosomia >4000 g, LGA (>90%), SGA (<10%), 5-min Apgar score (<7), low birthweight (<2500 g), intra-uterine growth restriction, antepartum haemorrhage after week 20, premature preterm rupture of membranes.

**Table S4. Summary of maternal adiposity exposures and maternal outcomes reported**

| **Paper** | **Exposure** | | | | | | | | | | | | | | | | **Outcome** | | | | | | | |
| --- | --- | --- | --- | --- | --- | --- | --- | --- | --- | --- | --- | --- | --- | --- | --- | --- | --- | --- | --- | --- | --- | --- | --- | --- |
|  | WC | HC | WHR | NC | AC | LC | FM | FFM | VF | SF | AT | SFT | Comp. | Other circ. | Other mass | Other ratio | GDM | I/G | HD | Comp. | MoD | Lipids | MS | GWG |
| Alptekin *et al. ^7^* | X | X | X |  |  |  |  |  |  |  |  |  |  |  |  |  | X |  |  |  |  |  |  |  |
| Alvarado *et al. ^15^* |  |  |  |  |  |  | X | X |  |  |  |  |  |  |  |  | X |  |  |  |  |  |  |  |
| Alves *et al. ^34^* |  |  |  |  |  |  |  |  | X |  |  |  |  |  |  |  | X |  |  |  |  |  |  |  |
| Bai *et al. ^35^* |  |  |  |  |  |  | X |  |  |  |  |  |  |  |  |  | X |  |  |  |  |  |  |  |
| Balani *et al. ^8^* |  |  | X |  |  |  | X |  | X |  |  |  |  |  |  |  | X |  |  |  |  |  |  |  |
| Bartha *et al. ^36^* |  |  |  |  |  |  |  |  | X | X |  |  |  |  |  | X |  | X | X |  |  | X |  |  |
| Basraon *et al. ^1^* |  |  | X |  |  |  |  |  |  |  |  |  |  |  |  |  | X |  |  |  |  |  |  |  |
| Bourdages *et al. ^37^* |  |  |  |  |  |  |  |  | X | X | X |  |  |  |  |  | X |  |  |  |  |  |  |  |
| Brisson *et al. ^38^* | X |  |  |  |  |  |  |  |  |  |  |  |  |  |  |  |  | X |  |  |  |  |  |  |
| Campbell *et al. ^39^* | X |  |  |  |  |  |  |  |  |  |  |  |  |  |  |  | X |  |  |  |  |  |  |  |
| Catov *et al. ^40^* | X |  |  |  |  |  |  |  |  |  |  |  |  |  |  |  | X |  |  |  |  |  |  |  |
| Dakshnamurthy *et al. ^41^* | X |  |  |  |  |  |  |  | X |  |  |  |  |  |  |  | X |  |  |  |  |  |  |  |
| De Souza *et al. ^42^* |  |  |  |  |  |  |  |  |  | X | X |  |  |  |  |  |  | X |  |  |  |  |  |  |
| De Souza *et al. ^43^* |  |  |  |  |  |  |  |  |  | X | X |  |  |  |  |  | X |  |  |  |  |  |  |  |
| De Souza *et al. ^44^* |  |  |  |  |  |  |  |  |  |  |  |  | X |  |  |  | X |  |  |  |  |  |  |  |
| Ebrahimi-Mameghani *et al. ^30^* | X |  |  |  |  |  |  |  |  |  |  |  |  |  |  |  | X |  | X |  |  |  |  |  |
| Egan *et al. ^20^* | X |  |  | X |  |  |  |  |  |  |  |  |  |  |  |  | X |  |  |  |  |  |  |  |
| Endeshaw *et al. ^45^* |  |  |  |  | X |  |  |  |  |  |  |  |  |  |  |  |  |  | X |  |  |  |  |  |
| Gagliardi *et al. ^33^* |  |  |  |  |  |  | X |  |  |  |  |  |  |  |  |  |  |  |  | X |  |  |  |  |
| Gao *et al. ^46^* | X |  |  |  |  |  |  |  |  |  |  |  | X |  |  |  | X |  |  |  |  |  |  |  |
| Gur *et al. ^47^* | X |  |  |  |  |  |  |  | X | X |  |  |  |  |  |  | X |  | X |  |  | X | X |  |
| Han *et al. ^31^* | X |  |  |  |  |  |  |  |  |  |  |  |  |  |  |  | X |  |  |  |  |  |  |  |
| Hancerliogullari *et al. ^21^* | X |  |  | X |  |  |  |  |  |  |  |  |  |  |  |  | X |  |  |  |  |  |  |  |
| Harville *et al. ^48^* | X |  |  |  |  |  |  |  |  |  |  |  |  |  |  |  | X |  |  |  |  |  |  |  |
| He *et al. ^22^* | X |  |  | X |  |  |  |  |  |  |  |  |  |  |  |  | X |  |  |  |  |  |  |  |
| Ianniello *et al. ^49^* |  |  |  |  |  |  | X | X |  |  |  |  |  |  |  |  | X |  |  |  |  |  |  |  |
| Iqbal *et al. ^16^* |  |  |  |  |  |  | X | X | X |  |  |  |  |  |  |  | X |  |  |  |  |  |  |  |
| Kausar *et al. ^27^* | X |  | X |  |  |  |  |  |  |  |  |  |  |  |  | X |  |  | X |  |  |  |  |  |
| Kennedy *et al. ^13^* |  |  |  |  |  |  |  |  |  | X |  |  |  |  |  |  | X |  | X |  | X |  |  |  |
| Khare *et al. ^3^* |  |  | X |  |  |  |  |  |  |  |  |  |  |  |  |  | X |  | X | X |  | X |  |  |
| KhushBakht *et al. ^50^* |  |  |  | X |  |  |  |  |  |  |  |  |  |  |  |  | X |  |  |  |  | X |  |  |
| Lacroix *et al. ^17^* | X |  |  |  |  |  | X |  |  |  |  |  |  |  |  |  | X |  |  |  |  |  |  |  |
| Lacroix *et al. ^51^* |  |  |  |  |  |  | X |  |  |  |  |  |  |  |  |  |  |  |  |  |  |  |  | X |
| Li *et al. ^71^* |  |  |  | X |  |  |  |  |  |  |  |  |  |  |  |  | X |  |  |  |  |  |  |  |
| Lima *et al. ^53^* | X |  |  |  | X | X |  |  | X | X |  | X |  |  |  |  |  |  |  |  |  |  | X |  |
| Liu *et al. ^18^* |  |  |  |  |  |  | X | X |  |  |  |  |  |  |  |  | X |  |  |  |  |  |  |  |
| Madhavan *et al. ^4^* | X |  | X |  |  |  |  |  |  |  |  |  |  |  |  |  | X |  | X |  | X |  |  |  |
| Maitland *et al. ^23^* | X | X |  |  |  |  |  |  |  |  |  |  |  |  |  |  | X |  |  |  |  |  |  |  |
| Martin *et al. ^54^* |  |  |  |  |  |  |  |  | X | X |  |  |  |  |  |  |  | X |  |  |  |  |  |  |
| McDonnold *et al. ^55^* |  |  | X |  |  |  |  |  |  |  |  |  | X |  |  |  |  |  |  |  |  |  |  |  |
| Mendoza *et al. ^32^* |  |  |  | X |  |  |  |  |  |  |  |  |  |  |  |  | X |  |  |  |  |  |  |  |
| Migda *et al. ^56^* | X |  | X |  |  |  |  |  |  |  |  |  |  |  |  |  | X |  |  |  | X |  |  |  |
| Minooee *et al. ^9^* | X | X | X |  |  |  |  |  |  |  |  |  |  |  |  |  | X |  |  |  |  |  |  |  |
| Mostafavi *et al. ^57^* | X |  |  |  |  |  |  |  |  |  |  |  |  |  |  |  |  | X |  |  |  | X |  |  |
| Oriji *et al. ^29^* | X |  |  |  |  |  |  |  |  |  |  | X |  |  |  |  | X |  |  |  |  |  |  |  |
| Piuri *et al. ^58^* | X |  |  |  |  |  | X | X |  |  |  | X |  |  | X |  |  |  | X |  |  |  |  |  |
| Pontual *et al. ^59^* |  |  |  |  |  |  |  |  | X |  |  |  |  |  |  |  |  | X |  |  |  | X |  |  |
| Popova *et al. ^60^* | X |  |  |  |  |  |  |  |  |  |  |  |  |  |  |  | X |  |  |  |  |  |  |  |
| Ray *et al. ^61^* |  |  |  |  |  |  |  |  | X |  |  |  |  |  |  |  |  |  | X |  |  |  |  |  |
| Salem *et al. ^62^* |  |  | X |  |  |  |  |  |  |  |  |  |  |  |  |  |  |  |  |  | X |  |  |  |
| Sattar *et al. ^63^* | X |  |  |  |  |  |  |  |  |  |  |  |  |  |  |  |  |  | X |  |  |  |  |  |
| Sharadha *et al. ^64^* | X |  |  |  |  |  |  |  |  |  |  |  |  |  |  |  | X |  | X |  |  |  |  |  |
| Sina *et al. ^28^* | X | X | X |  |  |  |  |  |  |  |  |  |  |  |  | X |  |  | X |  |  |  |  |  |
| Sina *et al. ^10^* | X | X | X |  |  |  |  |  |  |  |  |  |  |  |  | X | X |  |  |  |  |  |  |  |
| Suresh *et al. ^14^* |  |  |  |  |  |  |  |  |  | X |  |  |  |  |  |  | X |  |  | X | X |  |  |  |
| Sween *et al. ^26^* | X |  | X |  |  |  | X |  |  |  |  |  |  |  |  |  |  |  | X |  |  |  |  |  |
| Taebi *et al. ^25^* | X | X | X |  |  |  |  |  |  |  |  |  |  |  |  |  |  |  | X |  |  |  |  |  |
| Thaware *et al. ^12^* |  |  |  |  |  |  |  |  | X | X |  |  |  |  |  |  | X |  |  |  |  |  |  |  |
| Tomedi *et al. ^65^* | X |  |  |  |  |  |  |  |  |  |  | X |  |  |  |  |  | X |  |  |  |  |  |  |
| Vieira *et al. ^66^* | X |  |  |  | X |  |  |  |  |  |  |  |  |  |  |  |  |  | X |  |  |  |  |  |
| Wang *et al. ^5^* |  |  | X |  | X |  | X | X |  |  |  |  |  |  |  | X | X |  |  |  |  |  |  |  |
| Wang *et al. ^67^* |  |  |  |  |  |  | X | X |  |  |  |  |  |  |  |  |  |  | X |  |  |  |  |  |
| Wen *et al. ^68^* | X |  |  |  |  |  |  |  |  |  |  |  |  |  |  |  |  |  | X |  |  |  |  |  |
| White *et al. ^11^* | X | X | X | X | X | X |  |  |  |  |  | X |  | X |  | X | X |  |  |  |  |  |  |  |
| Yamamoto *et al. ^24^* |  |  | X |  |  |  |  |  |  |  |  |  |  |  |  |  |  |  | X |  |  |  |  |  |
| Yang *et al. ^69^* |  |  |  |  |  |  |  |  |  | X |  |  |  |  |  |  | X |  |  |  |  |  |  |  |
| Yeboah *et al. ^70^* |  |  |  |  |  |  | X | X |  |  |  |  |  |  | X |  |  |  | X |  |  |  |  |  |
| Zhang *et al. ^6^* |  |  | X |  |  |  |  |  |  |  |  |  |  |  |  |  | X |  |  |  |  |  |  |  |
| Zhang *et al. ^19^* |  |  |  |  |  |  | X | X | X |  |  |  |  |  |  |  | X |  |  |  |  |  |  |  |
| Zhu *et al. ^2^* | X |  | X |  |  |  |  |  |  |  |  |  |  |  |  |  | X |  |  |  |  |  |  |  |

WC - Waist circumference, HC - Hip circumference, WHR - Waist: hip ratio, NC - Neck circumference, AC - Arm circumference, LC - Leg circumference, FM - Fat mass, FFM - Fat-free mass, VF - Visceral fat, SF - Subcutaneous fat, AT - Adipose tissue, SFT – Skinfold thickness, Comp. - composite adiposity measurement, Other circ. – other circumference measurement (including wrist circumference), Other mass (including water mass, skeletal mass), Other ratio (including ratio of visceral fat thickness:subcutaneous fat thickness; neck:thigh; fat mass: fat free mass), GDM – gestational diabetes, I/G – insulin or glucose related outcomes, HD - Hypertensive disorders, Comp.- composite outcome, MoD – Mode of delivery related outcomes, Lipids - Maternal lipids, MS - Metabolic syndrome, GWG - Gestational weight gain**Table S5A. Quality scores for cohort studies (59 studies)**

| **Paper** | **Newcastle Ottawa Scale question number and score allocated ^a^** | | | | | | | | | |
| --- | --- | --- | --- | --- | --- | --- | --- | --- | --- | --- |
|  | **1** | **2** | **3** | **4** | **5** | **6** | **7** | **Total stars^b^** | **Rating^c^** | **Reviewer initials^d^** |
| Alptekin et al. 2016 ^7^ | b* | a* | d | c | b* | a* | b* | 5 | Medium | NH and AO |
| Alvarado et al. 2021 ^15^ | a* | a* | a* | b* | a* | a* | b* | 7 | High | AF and AO |
| Alves et al. 2020 ^34^ | a* | a* | a* | b* | a* | a* | b* | 7 | High | AF and AO |
| Bai et al. 2020 ^35^ | a* | a* | a* | b* | a* | a* | b* | 7 | High | LN and AO |
| Bartha et al. 2007 ^36^ | b* | a* | a* | b* | a* | a* | a* | 7 | High | LC and AO |
| Basraon et al. 2016 ^1^ | a* | a* | a* | b* | a* | a* | a* | 7 | High | LN and AO |
| Bourdages et al 2018 ^37^ | b* | a* | a* | b* | b* | a* | a* | 7 | High | NH and AO |
| Brisson et al. 2013 ^38^ | b* | c | a* | b* | a* | a* | a* | 6 | High | AO and LC |
| Campbell et al. 2012 ^39^ | a* | a* | a* | b* | b* | a* | a* | 7 | High | LN and LC |
| Catov et al. 2020 ^40^ | a* | a* | a* | b* | a* | a* | a* | 7 | High | LN and AO |
| De Souza et al. 2014 ^42^ | b* | a* | a* | b* | b* | a* | d | 6 | High | NH and AO |
| De Souza et al. 2016 ^43^ | b* | a* | a* | a* b* | b* | a* | b* | 8 | High | AF and AO |
| De Souza et al. 2016 ^44^ | a* | a* | a* | a* b* | a* | a* | a* | 8 | High | LN and LC |
| Ebrahimi-Mameghani et al. 2013 ^30^ | c | a* | a* | b* | d | a* | a* | 5 | Medium | AO and LC |
| Egan et al. 2017 ^20^ | c | a* | a* | b* | a* | a* | b* | 6 | High | LH and AO |
| Gao et al. 2017 ^46^ | a* | a* | a* | b* | b* | a* | b* | 7 | High | NH and AO |
| Gur et al. 2014 ^47^ | b* | a* | a* | b* | b* | a* | b* | 7 | High | AF and AO |
| Han et al. 2017 ^31^ | d | a* | a* | a* b* | b* | a* | b* | 7 | High | AF and AO |
| Hancerliogullari et al. 2020 ^21^ | a* | a* | a* | c | a* | a* | b* | 6 | High | LH and AO |
| Harville et al. 2014 ^48^ | b* | a* | a* | b* | b* | a* | b* | 6 | High | AF and AO |
| Ianniello et al. 2013 ^49^ | c | a* | a* | b* | b* | a* | a* | 6 | High | AF and AO |
| Iqbal et al. 2007 ^16^ | c | a* | a* | a* b* | a* | a* | b* | 7 | High | NH and AO |
| Kausar et al. 2013 ^27^ | d | a* | a* | b* | b* | a* | b* | 6 | High | NH and AO |
| Kennedy et al. 2016 ^13^ | b* | a* | a* | b* | b* | a* | b* | 7 | High | AS and AO |
| Khare et al. 2017 ^3^ | d | c | a* | b* | a* | a* | a* | 5 | Medium | NH and AO |
| KhushBakht et al. 2018 ^50^ | b* | a* | a* | c | a* | a* | a* | 6 | High | LC and AO |
| Lacroix et al. 2013 ^17^ | b* | a* | a* | c | a* | a* | d | 5 | Medium | LH and AO |
| Lacroix et al. 2016 ^51^ | a* | a* | a* | b* | b* | a* | d | 6 | High | NH and AO |
| Li et al. 2018 ^52^ | a* | a* | a* | c | a* | a* | a* | 6 | High | LH and AO |
| Lima et al. 2019 ^53^ | a* | a* | a* | b* | b* | a* | c | 6 | High | LH and AO |
| Liu et al. 2020 ^18^ | b* | a* | a* | c | b* | a* | a* | 6 | High | AF and AO |
| Madhava et al. 2008 ^4^ | a* | a* | a* | b* | a* | a* | b* | 7 | High | AS and AO |
| Maitland et al. 2014 ^23^ | d | a* | a* | b* | b* | a* | d | 5 | Medium | LH and AO |
| Martin et al. 2009 ^54^ | a* | a* | a* | b* | a* | a* | a* | 7 | High | AS and AO |
| McDonnold et al. 2016 ^55^ | c | a* | d | b* | b* | a* | a* | 5 | Medium | LN and AO |
| Mendozaet al. 2018 ^32^ | c | c | a* | c | a* | a* | a* | 4 | Medium | LC and AO |
| Migda et al. 2016 ^56^ | c | c | a* | c | a* | a* | a* | 4 | Medium | LC and AO |
| Minooee et al. 2017 ^9^ | b* | a* | a* | b* | a* | a* | c | 5 | Medium | AO and LC |
| Mostafavi et al. 2015 ^57^ | d | a* | a* | c | b* | a* | d | 4 | Medium | AS and AO |
| Oriji et al. 2017 ^29^ | b* | a* | a* | c | b* | a* | b* | 6 | High | AO and LC |
| Piuri, et al. 2016 ^58^ | a* | a* | a* | c | b* | a* | d | 5 | Medium | NH and AO |
| Pontual et al. 2016 ^59^ | b* | a* | a* | b* | a* | a* | d | 6 | High | NH and AO |
| Ray et al. 2017 ^61^ | b* | a* | a* | b* | d | a* | b* | 6 | High | AO and LC |
| Salem et al. 2012 ^62^ | d | a* | c | b* | b* | a* | d | 4 | Medium | AS and AO |
| Sattar et al. 2001 ^63^ | d | a* | a* | b* | b* | a* | d | 5 | Medium | AS and AO |
| Sharadha et al. 2016 ^64^ | b* | a* | a* | c | b* | a* | a* | 6 | High | LN and LC |
| Suresh et al. 2012 ^14^ | a* | a* | a* | b* | a* | a* | a* | 7 | High | LN and AO |
| Sween et al. 2015 ^26^ | b* | a* | a* | b* | a* | a* | b* | 7 | High | AF and AO |
| Taebi et al. 2015 ^25^ | a* | a* | a* | c | a* | a* | b* | 6 | High | LN and LC |
| Thaware et al. 2019 ^12^ | b* | a* | a* | b* | a* | a* | b* | 7 | High | AF and AO |
| Tomedi et al. 2014 ^65^ | a* | a* | a* | a* b* | a* | B | a* | 7 | High | LN and LC |
| Vieira et al. 2017 ^66^ | a* | a* | a* | b* | d | a* | b* | 6 | High | AO and LC |
| White et al. 2016 ^11^ | d | a* | a* | b* | a* | a* | b* | 6 | High | AS and AO |
| Wen et al. 2017 ^68^ | b* | a* | a* | b* | b* | a* | b* | 7 | High | AS and AO |
| Yang et al. 2017 ^69^ | b* | a* | a* | c | a* | a* | a* | 6 | High | AS and AO |
| Yeboah et al. 2017 ^70^ | b* | a* | a* | c | a* | B | b* | 5 | Medium | LH and AO |
| Zhang et al. 1995 ^6^ | a* | a* | a* | b* | b* | a* | c | 6 | High | LC and AO |
| Zhang et al. 2020 ^6^ | b* | a* | a* | b* | c | a* | a* | 6 | High | LH and AO |
| Zhu et al. 2019 ^2^ | a* | a* | a* | a* b* | a* | a* | b* | 8 | High | LH and AO |
| **Total** | **n*=44**  **74.6%** | **n*=55**  **93.2%** | **n*=56**  **94.9%** | **n*=50**  **42.4%** | **n*=55**  **93.2%** | **n*=57**  **96.6%** | **n*=47**  **79.6%** | **Range 5-8** | **Medium n=14 (23.7%)**  **High n=45 (76.3%** |  |

^a^ Newcastle-Ottawa question numbers 1-7, answers and associated number of stars (*) are detailed in Table S4C. ^b^ Minimum number of possible stars to be awarded = 0, maximum number of possible stars to be awarded = 8. ^c^ Categories were allocated as: Low = 0-2 stars, Medium = 3-5 stars, High = 6-8 stars. ^d^ Reviewers initials relate to manuscript authors: NH: Nicola Heslehurst, AF: Angela Flynn, LN: Lem Ngongalah, LC: Lisa Crowe, AS: Alexandre Simon, AO: Adefisayo Odeniyi

**Table S5B. Quality scores for case-control studies (11 studies)**

| **Paper** | **Newcastle Ottawa Scale question number and score allocated^a^** | | | | | | | | | | |
| --- | --- | --- | --- | --- | --- | --- | --- | --- | --- | --- | --- |
|  | **1** | **2** | **3** | **4** | **5** | **6** | **7** | **8** | **Total stars^b^** | **Rating^c^** | **Reviewer initials^d^** |
| Balani et al. 2014 ^8^ | a* | a* | a* | a* | b* | a* | a* | a* | 8 | High | LN and LH |
| Dakshnamurphy et al. 2017 ^41^ | a* | b | a* | a* | c | a* | a* | a* | 6 | Medium | LN and LH |
| Endeshaw et al. 2016 ^45^ | a* | b | a*` | a* | b* | a* | a* | a* | 7 | High | LN and LH |
| Gagliardi et al. 2016 ^33^ | a* | b | a* | a* | c | a* | a* | b* | 6 | Medium | LN and LH |
| He et al. 2017 ^22^ | a* | b | a* | a* | a* b* | a* | a* | c | 7 | High | LN and LH |
| Popova et al. 2015 ^60^ | a* | b | a* | a* | c | a* | a* | c | 5 | Medium | LN and LH |
| Sina et al. 2014 ^28^ | a* | b | a* | a* | b* | a* | a* | b* | 7 | High | LN and LH |
| Sina et al. 2015 ^10^ | a* | a* | a* | a* | b* | a* | a* | a* | 8 | High | LN and LH |
| Wang et al. 2019 ^5^ | a* | a* | a* | a* | b* | a* | a* | c | 7 | High | LN and LH |
| Wang, et al. 2015 ^67^ | a* | b | a* | a* | b* | a* | a* | a* | 7 | High | LN and LH |
| Yamamoto et al. 2001 ^24^ | a* | b | a* | a* | a* b* | a* | a* | c | 7 | High | LN and LH |
| **Total** | **n*=11**  **100%** | **n*=3**  **27.3%** | **n*=11**  **100%** | **n*=11**  **100%** | **n*=10**  **45.5%** | **n*=11**  **100%** | **n*=11**  **100%** | **n*=7**  **63.6%** | **Range**  **5-8** | **Medium n=3 (27.3%)**  **High n=8 (72.7%)** |  |

^a^ Newcastle-Ottawa question numbers 1-8, answers and associated number of stars (*) are detailed in Table S4D. ^b^ Minimum number of possible stars to be awarded = 0, maximum number of possible stars to be awarded = 9. ^c^ Categories were allocated as: Low = 0-3 stars, Medium = 4-6 stars, High = 7-9 stars. ^d^ Reviewers’ initials relate to manuscript authors: LH: Louise Hayes, LN: Lem Ngongalah

**Table S5C. Adapted Newcastle-Ottawa Scale for Cohort Studies**

| **Selection ^a, b^**  1) Representativeness of the exposed cohort (i.e. those with high adiposity measures) **– select one only**  a) truly representative of the average maternal population in the community (e.g. they recruited all women in a set time period) *  b) somewhat representative of the average maternal population in the community (e.g. they compared the population they recruited with the local population and they have similar characteristics) *****  c) selected group of users eg those with diabetes, over 35, restricted to one ethnic group, PCOS etc  d) no description of the derivation of the cohort  2) Selection of the non-exposed cohort (i.e. those with low adiposity measures) **– select one only**  a) drawn from the same community as the exposed cohort (it will usually be this answer if they have recruited women from the same unit at the same time) *****  b) drawn from a different source (e.g. recruited women from the same unit but at a different time, or from a different unit)  c) no description of the derivation of the non-exposed cohort  3) Ascertainment of exposure **– select one only**  a) secure record (eg explicitly measured adiposity) *****  b) structured interview (e.g. validated self-report such as asking to self-report the pre-pregnancy exposure and then validating this with an in-pregnancy measure – but used the self-report measure in the analysis as the exposure variable) *****  c) any self-report (invalidated)  d) no description  **Comparability**  4) Comparability of cohorts on the basis of the design or analysis - **a and b can both be selected**  a) study controls for change in adiposity in pregnancy (e.g. gestational weight gain) *****  b) study controls for any additional factor *****  c) no factors controlled for  **Outcome**  5) Assessment of outcome **– select one only**  a) independent blind assessment (e.g. specifically measured for the research) *****  b) record linkage (e.g. outcomes from routine medical records) *****  c) self-report  d) no description  6) Was follow-up long enough for outcomes to occur **– select one only**  a) yes (select an adequate follow up period for outcome of interest: followed up for long enough for the outcome to develop, e.g. GDM assessed late in pregnancy, all women followed up until delivery for preterm birth outcome etc) *****  b) no  7) Adequacy of follow up of cohorts **– select one only**  a) complete follow up - all subjects accounted for (i.e. no loss to follow up for prospective cohorts) *****  b) subjects lost to follow up unlikely to introduce bias - small number lost (>80% follow up for prospective cohorts, or for retrospective cohorts >80% with the data required for analysis) or description provided of those lost) *****  c) follow up rate < 80% or >20% excluded due to missing data for the analysis and no description of those lost  d) no statement  Total number of stars (out of a possible 8): |
| --- |

Abbreviation: BMI- Body mass index

a- The primary exposure is pre-pregnancy or early pregnancy adiposity measures (excluding BMI)

b- Original question 4 “Demonstration that outcome of interest was not present at start of study” in the original scale is not applicable to pregnancy outcomes as women are identified in early pregnancy using their pre/early pregnancy adiposity and the outcomes cannot exist at the start of the study. Therefore, this item has been removed from the scale. The denominator value for the maximum number of stars a study can be awarded has been reduced from 9 to 8 due to the removal of the original question 4.

**Table S5D. Adapted Newcastle-Ottawa Scale for Case-Control Studies**

| Selection ^a^  1) Is the case definition adequate?  a) yes, with independent validation*  b) yes, record linkage or based on self-reports with no reference to primary record  c) no description  2) Representativeness of the cases  a) consecutive or obviously representative series of cases*  b) potential for selection biases or not stated  3) Selection of Controls  a) community controls* (e.g., the same antenatal community (including same hospital/clinics) as cases)  b) Hospital controls (e.g., a different hospital or different clinics within hospitals, etc.)  c) no description  4) Definition of Controls  a) no history of disease (endpoint)*  b) no description of source  Comparability  5) Comparability of cases and controls on the basis of the design or analysis (*both a and b can be selected*)  a) study controls for change in adiposity in pregnancy (e.g. gestational weight gain) *  b) study controls for any additional factor*  c) no factors controlled for  Exposure  6) Ascertainment of exposure  a) secure record (e.g., explicitly measured adiposity)*  b) structured interview where blind to case/control status  c) interview not blinded to case/control status (e.g., adiposity data is collected retrospectively after the outcome case/control status is known)  d) written self-report or medical record only  e) no description  7) Same method of ascertainment for cases and controls  a) yes*  b) no  8) Non-Response rate  a) same rate for both groups*  b) non respondents described  c) rate different and no designation  Total number of stars (out of a possible 9): |
| --- |

Abbreviation: BMI- Body mass index.

a- The primary exposure is pre-pregnancy or early pregnancy adiposity measures (excluding BMI)

**Table S6A. Gestational diabetes mellitus (association data reported)**

| Paper | Outcome definition | Sample size | Reference group | Comparison groups | Result * | Adjustments |
| --- | --- | --- | --- | --- | --- | --- |
| Waist circumference (WC) |  |  |  |  |  |  |
| Popova et al. 2015 ^60^ | GDM, IADPSG criteria | 548 | Not reported | >80cm | \| **OR 2.7 (95% CI 1.7, 4.2)** \| \| --- \| | None |
| Gao et al. 2017 ^46^ | GDM, IADPSG criteria | 919 | <80cm | ≥80cm | **AOR 2.26 (95% CI 1.11, 4.60)** | Street, maternal and paternal age/ education/ BMI, active or passive smoking, alcohol consumption, family income, parity, infant gender, gestational age |
| Ebrahimi-Mameghani et al. 2013 ^30^ | GDM, glucose intolerance of variable severity with onset/first detection during pregnancy | 948 | <80cm | 1: 80-88cm  2: >88cm | 1: AOR 0.39 (95% CI 0.04, 3.38)  **2: AOR 3.77 (95% CI 2.91, 10.41)** | Preeclampsia, gestational hypertension, pregnancy delivery |
| Zhu et al. 2019 ^2^ | GDM, Carpenter & Coustan criteria | 1750 | 61-80cm | 1: 81-88cm  2: 89-99cm  3: 100-166 | 1: AOR 1.85 (95% CI 0.48, 7.16)  2: AOR 2.98 (95% CI 0.71, 12.6)  **3: AOR 6.35 (95% CI 1.07, 37.7)** | Pre-pregnancy BMI, family history of diabetes, previous gestational diabetes, and pre-existing hypertension. |
| Han et al. 2017 ^31^ | GDM, IADPSG criteria | 17803 | <78.5cm | 1: ≥78.5 to <85cm  2: ≥85cm | **1: AOR 1.18 (95% CI 1.00, 1.40)**  **2: AOR 1.60 (95% CI 1.34, 1.91)** | Age, height, first-degree family history of DM, gestation at registration, parity, education, nationality, non-singleton, systolic BP, weight gain/week, pre-pregnancy smoking/drinking, BMI |
| He et al. 2017 ^22^ | GDM, ADA criteria | 255 | Categories not defined | Categories not defined | **AOR 1.22 (95% CI 1.01, 1.46)** | HbA1c, FPG, 2-hour glucose, 1-h glucose, neck circumference |
| Campbell et al. 2012 ^39^ | GDM, confirmed by doctors on a medical chart audit or fasting glucose ≥7 mmol/l or 2-h glucose tolerance test result of 11.1 mmol | 177 | <80cm | 1: 80-87.9cm  2: ≥88cm | 1: OR 0.82 (95% CI 0.14, 4.71)  2: OR 3.12 (95% CI 1.00, 9.69) | None |
| Hancerliogullari et al. 2020 ^21^ | GDM, Carpenter & Coustan critieria | 525 | <84.5cm | >84.5cm | **OR 3.58 (95% CI 1.77– 7.22)** | Gravidity, parity, BMI, and neck circumference |
| Sina et al. 2015 ^10^ | GDM, ICD codes | 131 | Continuous | per 1 SD increase in WC | **AOR 1.82 (95% CI** **1.12, 2.96)** | Age, total number of pregnancies during study period, smoking status, drinking consumption |
| Han et al. 2017 ^31^ | GDM, IADPSG criteria | 17803 | Continuous | per 1 SD increase in WC | **AOR 1.26 (95% CI 1.17, 1.34)** | Age, height, first-degree family history of DM, gestation at registration, parity, education, nationality, non-singleton, systolic BP, weight gain/week, pre-pregnancy smoking/drinking, BMI |
| Harville et al. 2014 ^48^ | GDM, defined as failing a GTT, or ICD-9 code 6488/ICD-10 code O24. | 349 | Continuous | per 1 SD increase in WC | **AOR 1.66 (95% CI 1.16, 2.38)** | Age, parity |
| White et al. 2016 ^11^ | GDM, IADPSG criteria (OB only) | 805 | Continuous | per 1cm increase in WC | **AOR 1.02 (95% CI 1.01, 1.04)** | age, systolic BP, waist circumference, HbA1c, random glucose, adiponectin |
| Migda et al. 2016 ^56^ | GDM, criteria not defined | 157 | Continuous | Unit of increase not defined | AUROC 0.74, p not reported | None |
| Madhavan et al. 2008 ^4^ | GDM, abnormal carbohydrate intolerance with onset/first detected during pregnancy | 106 | Continuous | Unit of increase not defined | **Correlation (r) 0.4, p<0.01** | None |
| Sharadha et al. 2016 ^64^ | GDM, DIPSI criteria (blood sugars >140 mg/dl after 2hr 75g GCT) | 239 | 1: <80cm (Asian-specific criteria)  2: <88cm (General population criteria) | 1: >80cm  2: >88cm | 1: Sensitivity 74.1%, Specificity 62%, PPV 22.5%, NPV 94.9%  2: Sensitivity 16.1%, Specificity 86%, PPV 14.7%, NPV 87.3% | None |
| Waist: hip ratio (WHR) |  |  |  |  |  |  |
| Madhavan et al. 2008 ^4^ | GDM, abnormal carbohydrate intolerance with onset/first detected during pregnancy | 212 | ≤0.85 | >0.85 | **OR 12.06 (1.43, 101.97)** | None |
| Basraon et al. 2016 ^1^ | GDM, defined using guidelines of each clinical centre | 1: 1235  2: 1709 | <0.80 | 1: 0.80-0.84  2: ≥0.85 | 1: AOR 1.64 (95% CI 0.76, 3.54)  **2: AOR 2.65 (95% CI 1.34**, **5.25)** | Age, education, race, weeks of gestation at enrolment, alcohol and smoking status |
| Zhang et al. 1995 ^6^ | GDM, IADPSG criteria | 720 | 0.629-0.705 | 1: 0.706 - 0.742  2: 0.743 - 1.020 | **1:** ARR 2.28 (95% CI 0.83, 6.25)  **2: ARR 3.00 (95% CI 1.08**, **8.35)** | BMI, age, race, family history of diabetes in first degree relatives, baseline parity and fasting insulin |
| Zhu et al. 2019 ^2^ | GDM, Carpenter & Coustan criteria | 1750 | Q1 (0.68-0.80) | 1: Q2 (0.81-0.85)  2: Q3 (0.86-0.91)  3: Q4 (0.92-1.18) | 1: AOR 1.72 (95% CI 0.43, 6.91)  2: AOR 3.27 (95% CI 0.83, 12.9)  **3: AOR 6.59 (95% CI 1.74, 24.4)** | Pre-pregnancy BMI, family history of diabetes, previous gestational diabetes, and pre-existing hypertension. |
| Wang et al. 2019 ^5^ | GDM, IADPSG criteria | 2698 | ≤0.85cm | >0.85 | **OR 1.81 (95% CI 1.48**, **2.21)** | None |
| Khare et al. 2017 ^3^ | GDM, criteria not defined | 120 | ≤0.8 cm | >0.8 | OR 0.85 (95% CI 0.03, 21.49) | None |
| Sina et al. 2015 ^10^ | GDM, ICD 9 and 10-AM codes | 131 | Continuous | 1 SD increase in WHR | AOR 1.65 (95% CI 0.94, 2.91) | Age, number of pregnancies, smoking status, drinking consumption |
| Visceral fat (measured by ultrasound unless stated) | | | | | | |
| De Souza et al. 2016 ^43^ | GDM, IADPSG criteria | 485 | ≤3.0 cm | 1: 3.1-3.8 cm  2: 3.9-4.8 cm  3: >4.8 cm | 1: AOR 1.5 (95% CI 0.67, 3.3)  2: AOR 1.3 (95% CI 0.57, 2.9)  **3: AOR 3.4 (95% CI 1.5, 8.0)** | Age, ethnicity, family history of diabetes, BMI, BMI change |
| Thaware et al. 2019 ^12^ | GDM, IADPSG/WHO 2013 criteria | 80 | Continuous | Per 1 SD increase in VAD | **AOR 2.09 (95% CI 1.06, 4.12)** | Age, gravidity, years in education, BMI |
| Alves et al. 2020 ^34^ | GDM, IADPSG criteria | 518 | Continuous | Per 1 cm increase in VAD | **AOR 2.0 (95% CI 1.61, 2.50)** | Maternal age, pre-pregnancy BMI |
| Zhang et al 2020 ^19^ | GDM, IADPSG criteria | 22223 | Continuous (bio-impedance) | Per 1 SD increase in visceral fat levels (no units) | **OR 2.60 (95% CI 2.46, 2.76)** | None |
| Bourdages et al. 2018 ^37^ | GDM, CDA criteria | 1048 | Continuous | n/a | AUROC 0.69 (95% CI 0.62, 0.77) | Maternal age, BMI |
| Subcutaneous fat (measured by ultrasound) | | | | | | |
| De Souza et al. 2016 ^43^ | GDM, IADPSG criteria | 485 | ≤1.3cm | 1: 1.4-1.7 cm  2: 1.8-2.3 cm  3: >2.3cm | 1: AOR 1.2 (95% CI 0.56, 2.7)  2: AOR 1.5 (95% CI 0.73, 3.3)  3: AOR 2.0 (95% CI 0.95, 4.5) | Maternal age, ethnicity, family history of diabetes, BMI, BMI change |
| Yang et al. 2017 ^69^ | GDM, National diabetes data group criteria | 72 | <2.4cm | ≥2.4cm | AOR 2.96 (95% CI 0.95, 9.25) | Pre-pregnancy body mass index |
| Kennedy et al. 2016 ^13^ | GDM, criteria not defined | 1461 | Continuous | Per 5mm increase in SF | **AOR 1.22 (95% CI 1.06**, **1.14)** | BMI, maternal age, parity, smoking status |
| Thaware et al. 2019 ^12^ | GDM, IADPSG/WHO criteria | 80 | Continuous | Per SD increase in SF | **AOR 0.62 (95% CI 0.27**, **1.44)** | Age, gravidity, education, pre-pregnancy BMI |
| Suresh et al. 2012 ^14^ | GDM, criteria not defined | 1200 | Continuous | Per 5mm increase in SF | **AOR 1.08 (95% CI 1.04, 1.12)** | Maternal age |
| Bourdages et al. 2018 ^37^ | GDM, CDA criteria | 1048 | Continuous | n/a | AUROC 0.69 (95% CI 0.62–0.76) | Combining maternal age and BMI |
| Fat mass |  |  |  |  |  |  |
| Wang et al. 2019 ^5^ | GDM, IADPSG Criteria | 2698 | ≤0.44 fat mass /fat free mass ratio | >0.44 | **OR 1.819 (95% CI 1.48**, **2.23)** | None |
| Wang et al. 2019 ^5^ | GDM, IADPSG Criteria | 2698 | ≤6.3 fat mass index (FMI) kg/m^2^ | >6.3 | **OR 2.014 (95% CI 1.64**, **2.48)** | None |
| Wang et al. 2019 ^5^ | GDM, IADPSG Criteria | 2698 | ≤25 fat mass % | >25 fat mass % | **AOR 1.79 (95% CI 1.11**, **2.87)** | Age, pre-pregnancy BMI |
| Liu et al. 2020 ^18^ | GDM, IADPSG criteria | 1318 | 18-27% | ≥28% | **AOR 1.57 (1.10, 2.24)** | Age, pre-pregnancy BMI |
| Zhang et al 2020 ^19^ | IADPSG criteria | 22223 | Continuous | Per 1SD increase FM, kg | **OR 1.085 (95% CI 1.079**, **1.091)** | None |
| Iqbal et al. 2007 ^16^ | GDM, ADA criteria | 612 | Continuous | Per 1 % increase in body fat | **AOR 1.07 (95% CI 1.03**, **1.13)** | None |
| Bai et al. 2020 ^35^ | GDM, IADPSG criteria | 117 | Continuous | Per 1% increase | **AOR 1.117 (95 % CI 1.02**, **1.22)** | Maternal age, trimester 1 Body Fat % (BFP), change in BFP, and initial BMI are for one unit increase |
| Ianniello et al. 2013 ^49^ | GDM, Carpenter & Coustan criteria (OW/OB) | 32 | Continuous FM % | n/a | R^2^, regression coefficient 0.038±0.01 | None |
| Fat-free mass (FFM) |  |  |  |  |  |  |
| Zhang et al. 2020 ^19^ | GDM, IADPSG criteria | 22223 | Continuous | Per 1 SD increase in FFM, Kg | **OR 1.08 (95% CI 1.10**, **1.12)** | None |
| Zhang et al. 2020 ^19^ | GDM, IADPSG criteria | 22223 | Continuous | Per 1 SD increase in lean right arm mass, Kg | **OR 3.40 (95 % CI 3.15**, **3.68)** | None |
| Zhang et al. 2020 ^19^ | GDM, IADPSG criteria | 22223 | Continuous | Per 1 SD increase in lean left arm mass, Kg | **OR 3.33 (95% CI 3.08**, **3.596)** | None |
| Zhang et al. 2020 ^19^ | GDM, IADPSG criteria | 22223 | Continuous | Per 1 SD increase in lean right leg mass, Kg | **OR 1.57 (95% CI 1.52, 1.62)** | None |
| Zhang et al. 2020 ^19^ | GDM, IADPSG criteria | 22223 | Continuous | Per 1 SD increase in lean left leg mass, Kg | **OR 1.57 (95% CI 1.52, 1.62)** | None |
| Zhang et al. 2020 ^19^ | GDM, IADPSG criteria | 22223 | Continuous | Per 1 SD increase in lean trunk mass, Kg | **OR 1.23 (95% CI 1.21, 1.24)** | None |
| Wang et al. 2019 ^5^ | GDM, IADPSG criteria | 2698 | ≤69.58 FFM % | >69.58 FFM% | **OR 0.55 (95% CI 0.451, 0.680)** | None |
| Arm circumference |  |  |  |  |  |  |
| White et al. 2016 ^11^ | GDM, IADPSG criteria (OB only) | 805 | Continuous | Per 1cm increase in mid-arm circumference | OR 1.03 (95% CI 0.99, 1.08) | None |
| Wang et al. 2019 ^5^ | GDM, IADPSG criteria | 2698 | ≤26.8cm | >26.8cm | **OR 1.688 (1.377, 2.069)** | None |
| Neck circumference (NC) |  |  |  |  |  |  |
| He et al. 2017 ^22^ | GDM, ADA criteria | 255 | Categories not defined | Categories not defined | **AOR 1.84 (95% CI 1.04, 3.25)** | HbA1c, FPG, 2-h glucose, 1-h glucose, waist circumference |
| Hancerliogullari et al. 2020 ^21^ | GDM, Carpenter and Coustan criteria | 525 | <38.5cm | >38.5cm | AOR 0.83 (95% CI 0.36, 1.91) | Age, gravidity, parity, BMI, waist circumference |
| Li et al. 2018 ^52^ | GDM, IADPSG criteria | 371 | Continuous | Per 1cm increase in NC | **AOR 1.29 (95% CI 1.11, 1.50)** | Gravidity, parity |
| Mendoza et al. 2018 ^32^ | GDM, IADPSG/WHO 2013 criteria (OB only) | 971 | Continuous | Per 1 cm increase in NC | **AOR 1.15 (95% CI 1.06, 1.24)** | First-degree relative with DM, ethnicity, education, marital/working status, PCOS, hypertension, smoking, age, height, weight, BMI, recruitment site, new-born sex, season/ gestation of OGTT, DALI intervention, previous: abnormal GT, pregnancies, GDM, stillbirth, congenital malformations, macrosomia |
| KhusBakht et al. 2018 ^50^ | GDM, not defined | 90 | Continuous | n/a | AUROC cut-off value for NC 35.70cm, Sensitivity s 0.51, specificity 0.812 | None |
| Hip circumference (HC) |  |  |  |  |  |  |
| Sina et al. 2015 ^10^ | GDM, ICD 9 and 10-AM codes | 131 | Continuous | Per 1 SD increase in HC | AOR 1.57 (95% CI 0.99, 2.48) | Age, total number of pregnancies during study period, smoking and drinking |
| Waist: height ratio (WHtR) |  |  |  |  |  |  |
| Sina et al. 2015 ^10^ | GDM, ICD 9 and 10-AM codes | 131 | Continuous | Per 1 SD increase in WHtR | **AOR 2.29 (95% CI 1.35, 3.88)** | Age, total number of pregnancies during study period, smoking and drinking |
| White et al. 2016 ^11^ | GDM, IADPSG criteria (OB only) | 1267 | Continuous | Per 0.1 increase in WHtR | **OR 1.57 (95% CI 1.25, 1.98)** | None |
| Total adipose tissue |  |  |  |  |  |  |
| De Souza et al. 2016 ^43^ | GDM, IADPSG criteria | 485 | ≤4.5 cm | 1: 4.6-5.5cm  2: 5.6-7.0cm  3: >7cm | 1: AOR 1.0 (95% CI 0.26, 1.5)  2: AOR 1.3 (95% CI 0.60, 2.7)  **3: AOR 3.4 (95% CI 1.6, 7.7)** | Maternal age, ethnicity, family history of diabetes, BMI, and BMI change |
| Bourdages et al. 2018 ^37^ | GDM, CDA criteria | 61 | Continuous | n/a | AUROC 0.70 (95% CI 0.62, 0.77) | Maternal age, BMI |
| Composite adiposity measures | | | | | | |
| De Souza et al. 2016 ^44^ | GDM composite outcome (defined as IFG, GIGT, or GDM) | 476 | Hepatic fat absent + VAT Q1-Q3 (≤4.8 cm) | 1: Hepatic fat present +VAT Q1-Q3 (≤4.8 cm)  2: Hepatic fat absent + VAT Q4 (>4.8 cm)  3: Hepatic fat present + VAT Q4 (>4.8 cm) | 1: AOR 1.4 (95% CI 0.45, 4.5)  2: AOR 2.3 (95% CI 1.0, 5.4)  **3: AOR 6.5 (95% CI 2.3, 18.5)** | Maternal age at delivery, ethnicity, family history of T2DM, body mass index (BMI) at 11- 14 weeks and change in BMI between 11-14 weeks and 24, 28 weeks |
| De Souza et al. 2016 ^44^ | GDM composite outcome (defined as IFG, GIGT, or GDM) | 476 | Hepatic fat absent + TAT Q1-Q3 (≤7.0 cm) | 1: Hepatic fat present + TAT Q1-Q3 (≤7.0 cm)  **2:** Hepatic fat absent + TAT Q4 (>7.0 cm)  3: Hepatic fat present + TAT Q4 (>7.0 cm) | 1: AOR 0.98 (95% CI 0.27, 3.5)  2: AOR 2.2 (95% CI 0.92, 5.2)  **3: AOR 7.8 (95% CI 2.8, 21.7)** | Maternal age at delivery, ethnicity, family history of T2DM, body mass index (BMI) at 11- 14 weeks and change in BMI between 11-14 weeks and 24, 28 weeks |
| Skinfold thickness (SFT) |  |  |  |  |  |  |
| White et al. 2016 ^11^ | GDM, IADPSG criteria (OB only) | 770 | Continuous | Per 1mm increase in sum of SFT (triceps, biceps, subscapular, suprailiac) | **AOR 1.01 (95% CI 1.01, 1.02)** | Biomarkers and metabolome model |
| Oriji et al. 2017 ^29^ | GDM, WHO criteria | 235 | Abdominal SFT ≤20mm | Abdominal SFT >20mm | **AOR: 21.71 (95% CI 8.33, 56.63)** | Previous history of GDM, BMI, maternal weight, SBP, DBP |
| Neck: thigh ratio (NTR) |  |  |  |  |  |  |
| White et al. 2016 ^11^ | GDM, IADPSG criteria (OB only | 770 | continuous | Per 0.1 increase in NTR | **AOR 1.52 (95% CI 1.11, 2.08)** | biomarkers and metabolome model |

Abbreviation: ADA- American Diabetes Association; IADPSG- International Association of Diabetes and Pregnancy Study Groups; ICD- International Classification of Disease; CDA- Canadian Diabetes Association; DIPSI- Diabetes in Pregnancy Study Group of India; GTT- Glucose tolerance test; VAD- Visceral adipose tissue depth; VAT-visceral adipose tissue; BMI-body mass index; SD- Standard deviation; SE- Standard error; IQR- Inter-quartile range; NR- Not reported; HbA1c- Hemoglobin A1C; FPG-Fasting plasma glucose; NTR- Neck: thigh ratio; SFT-skinfold thickness; WHtR- Waist: height ratio; HC-hip circumference; NC-neck circumference; FFM-fat-free mass; WHR-waist: hip ratio; WC-waist circumference

* Green highlighted results included in categorical meta-analysis, yellow highlighted results included in continuous meta-analysis, bold data signify statistically significant results.

**Table S6B. Gestational diabetes mellitus (case control data reported)**

| Paper | Outcome definition | Sample size | Data reported | Controls (no GDM) | Cases (GDM) | Significance, p value |
| --- | --- | --- | --- | --- | --- | --- |
| Waist circumference (WC) | | | | | | |
| Minooee et al. 2017 ^9^ | GDM, WHO criteria | 2458 | Mean WC (SD), cm | **84.7 (10.7)** | **89.0 (11.5)** | **<0.001** |
| Sina et al. 2015 ^10^ | GDM, ICD 9 and 10-AM codes | 131 | Mean WC (SD), cm | **81.3 (12.8)** | **90.3 (16.4)** | **0.003** |
| Lacroix et al. 2013 ^17^ | GDM, IADPSG criteria (OW+OB) | 445 | Mean WC (SD), cm | 90.0 (12.9) | 94.8 (18.6) | 0.12 |
| Alptekin et al. 2016 ^7^ | GDM, Carpenter & Coustan criteria | 227 | Mean WC (SD), cm | **80.8 (10.5)** | **89.7 (11.9)** | **<0.001** |
| Dakshnamurthy et al. 2017 ^41^ | GDM, not defined | 60 | Mean WC (SD), cm | **88.5 (7.1)** | **102.1 (6.96)** | **<0.0001** |
| Han et al. 2017 ^31^ | GDM, IADPSG criteria | 17803 | Mean WC (SD), cm | **78.5 (8.6)** | **82.8 (9.7)** | **<0.05** |
| Harville et al. 2014 ^48^ | GDM, not defined | 349 | Mean WC (SD), cm | **77.2 (9.6)** | **84.2 (12.4)** | **0.01** |
| He et al. 2017 ^22^ | GDM, American Diabetes Association | 255 | Mean WC (SD), cm | **97.95 (6.25)** | **103.16 (8.00)** | **<0.001** |
| Popova et al. 2015 ^60^ | GDM, IADPSG criteria | 548 | Mean WC (SD), cm | **82.2 (11.4)** | **92.6 (15.4)** | **<0.001** |
| Campbell et al. 2012 ^39^ | GDM, onfirmed by doctors on medical chart audit or fasting glucose C7 mmol/l or 2-h GTT result of 11.1 mmol | 220 | Mean WC (SD), cm | 87.9 (15.2) | 102.1 (18.3) | NR |
| Zhu et al. 2019 ^2^ | GDM, Carpenter & Coustan criteria | 1750 | Mean WC (SD), cm | **90.3 (14.2)** | **102.4(18.5)** | **<0.001** |
| Oriji et al. 2017 ^29^ | GDM, WHO 2013 criteria | 235 | Mean WC (SE), cm | 107.80 (1.40) | 108.00 (1.30) | 0.32 |
| Egan et al. 2017 ^20^ | GDM, IADPSG/ WHO 2013 criteria in mid/late pregnancy (>20 weeks, OB only) | 1023 | Mean WC (SD), cm | 107.3 (10.1) | 107.1 (10.1) | NS (value not reported) |
| Maitland et al. 2014 ^23^ | GDM, IADPSG criteria (OB only) | 106 | Mean WC (SD), cm | 107.6 (10.8) | 107.8 (7.4) | 0.88 |
| Catov et al. 2020 ^40^ | GDM, self-reported and validated from prenatal records | 1302 | Mean WC (SD), cm | **72.0 (9.6)** | **75.3(13.3)** | **<0.01** |
| Hancerliogullari et al. 2020 ^21^ | GDM, Carpenter & Coustan criteria | 525 | Mean WC (SD), cm | **84.17 (10)** | **91.78 (11.4)** | **0.01** |
| Waist: hip ratio (WHR) |  |  |  |  |  |  |
| Minooee et al. 2017 ^9^ | GDM, WHO definition | 2458 | Mean WHR (SD) | **0.81 (0.07)** | **0.83 (0.07)** | **<0.001** |
| Sina et al. 2015 ^10^ | GDM, ICD 9 and 10-AM codes | 131 | Mean WHR (SD) | 0.89 (0.08) | 0.92 (0.05) | 0.09 |
| Balani et al. 2014 ^8^ | GDM, WHO criteria (OB only) | 302 | Mean WHR (SD) | **0.99 (0.05)** | **1.02 (0.07)** | **<0.01** |
| Alptekin et al. 2016 ^7^ | GDM, Carpenter & Coustan criteria | 277 | Mean WHR (SD) | **0.81 (0.05)** | **0.84 (0.04)** | **0.004** |
| Basraon et al. 2016 ^1^ | GDM, defined using guidelines of each clinical centre | 2300 | Mean WHR (SD) | **0.84 (0.08)** | **0.88 (0.07)** | **p<0.0001** |
| White et al. 2016 ^11^ | GDM, IADPSG criteria (OB only) | 1303 | Mean WHR (SD) | **0.87 (0.08)** | **0.89 (0.07)** | **p <0.001** |
| Zhu et al. 2019 ^2^ | GDM, Carpenter & Coustan criteria | 1750 | Mean WHR (SD) | **0.86 (0.07)** | **0.91 (0.06)** | **<0.001** |
| Wang et al. 2019 ^5^ | GDM, IADPSG Criteria | 2698 | Mean WHR (SD) | **0.85 (0.04)** | **0.86 (0.04)** | **<0.001** |
| Visceral fat (VF) |  |  |  |  |  |  |
| Balani et al. 2014 ^8^ | GDM, WHO criteria (OB only) | 302 | Mean VF mass (SD), mm | **183.8 (31.5)** | **199.2 (40.5)** | **≤0.01** |
| Gur et al. 2014 ^47^ | GDM, diagnosed as patients with two of: FBG ≥105 mg/dL, 1h glucose ≥190 mg/dL, 2h glucose ≥165 mg/dL, and 3h glucose ≥145 mg/dL | 88 | Mean VF thickness (SE), mm | **10 (0.50)** | **15.2 (1.4)** | **P<0.0001** |
| Zhang et al 2020 ^19^ | GDM, IADPSG criteria | 22223 | Mean VF levels (SD), no units | **8.21 (0.51)** | **8.48 (0.56)** | **<0.001** |
| Thaware et al. 2019 ^12^ | GDM, IADPSG/WHO criteria | 80 | Mean VAT depth (SD), cm | **4.19 (1.24)** | **5.09 (1.43)** | **0.02** |
| Alves et al. 2020 ^34^ | GDM, IADPSG criteria | 518 | Mean VAT depth (SD), cm | **5.2 (1.1)** | **6.3 (1.3)** | **0.001** |
| Dakshnamurthy et al. 2017 ^41^ | GDM, not defined | 60 | Mean visceral adiposity index (SD) | **5.54 (2.41)** | **9.91 (5.73)** | **0.0003** |
| Fat mass (FM) |  |  |  |  |  |  |
| Liu et al. 2020 ^18^ | GDM, IADPSG criteria | **1318** | Mean FM (SD), % | **27.5 (5.6)** | **30.1 (5.8)** | **<0.001** |
| Balani et al. 2014 ^8^ | GDM, WHO criteria (OB only) | 302 | Mean body fat (SD), % | 49.2 (3.6) | 49.8 (3.5) | NS (p not reported) |
| Lacroix et al. 2013 ^17^ | GDM, IADPSG criteria (OW+OB) | 445 | Mean body fat (SD), % | 31.3 (8.3) | 33.6 (11.4) | 0.24 |
| Iqbal et al. 2007 ^16^ | GDM, ADA criteria | 612 | Mean body fat (SD), % | **27.4 (7.8)** | **32.2 (6.4)** | **<0.001** |
| Wang et al. 2019 ^5^ | IADPSG criteria | 2698 | Mean body fat (SD), % | **29.84 (5.03)** | **31.55 (-4.81)** | **<0.001** |
| Zhang et al. 2020 ^19^ | IADPSG criteria | 22223 | Mean body fat (SD), % | **28.19 (5.93)** | **30.09 (5.69)** | **<0.001** |
| Alvarado et al. 2021 ^15^ | GDM, Carpenter & Coustan criteria | 46 | Mean body fat (SD), % | 28 (8) | 30 (9) | 0.54 |
| Ianniello et al. 2013 ^49^ | GDM, Carpenter & Coustan criteria (OW/OB only) | 32 | Mean FM (SE), % | **38.13 (0.69)** | **42.67 (1.56)** | **<0.05** |
| Liu et al. 2020 ^18^ | GDM, IADPSG criteria | **1318** | Mean FM index (SD), kg/m^2^ | **6.00 (1.91)** | **7.14 (2.26)** | **<0.001** |
| Wang et al. 2019 ^5^ | IADPSG criteria | 2698 | Mean FM index (SD), kg/m^2^ | **6.33 (1.69)** | **7.0 (1.81)** | **<0.001** |
| Iqbal et al. 2007 ^16^ | GDM, ADA criteria | 612 | Mean FM (SD), kg | **16.8 (7.5)** | **20.7 (6.5)** | **<0.001** |
| Zhang et al. 2020 ^19^ | IADPSG criteria | 22223 | Mean FM (SD), kg | **15.51 (5.18)** | **17.95 (5.65)** | **<0.001** |
| Alvarado et al. 2021 ^15^ | GDM, Carpenter & Coustan criteria | 46 | Mean FM (SD), kg | 19 (10) | 21 (12) | 0.44 |
| Wang et al. 2019 ^5^ | IADPSG criteria | 2698 | Median FM:FFM (IQR), ratio | **0.43 (0.14)** | **0.47 (0.14)** | **<0.001** |
| Fat-free mass (FFM) |  |  |  |  |  |  |
| Zhang et al. 2020 ^19^ | GDM, IADPSG criteria | 22223 | Mean FFM (SD), kg | **38.42 (4.29)** | **40.56 (4.92)** | **<0.001** |
| Iqbal et al. 2007 ^16^ | GDM, ADA criteria | 612 | Mean FFM (SD), Kg | 41.4 SD 3.8 | 42.0 SD 3.0 | p=0.29 |
| Alvarado et al. 2021 ^15^ | GDM, Carpenter& Coustan criteria | 46 | Mean FFM (SD), kg | 44 (5) | 46 (4) | 0.09 |
| Zhang et al. 2020 ^19^ | GDM, IADPSG criteria | 22223 | Mean lean right leg mass (SD), Kg | **5.93 (0.94)** | **6.36 (1.12)** | **<0.001** |
| Zhang et al. 2020 ^19^ | GDM, IADPSG criteria | 22223 | Mean lean left leg mass (SD), Kg | **5.93 (0.94)** | **6.35 (1.12)** | **<0.001** |
| Zhang et al. 2020 ^19^ | GDM, IADPSG criteria | 22223 | Mean lean right arm mass (SD), Kg | **1.65 (0.39)** | **1.86 (0.46)** | **<0.001** |
| Zhang et al. 2020 ^19^ | GDM, IADPSG criteria | 22223 | Mean lean left arm mass (SD), Kg | **1.60 (0.40)** | **1.81 (0.47)** | **<0.001** |
| Zhang et al. 2020 ^19^ | GDM, IADPSG criteria | 22223 | Mean lean trunk mass (SD), Kg | **17.28 (2.13)** | **18.32 (2.47)** | **<0.001** |
| Ianniello et al. 2013 ^49^ | GDM, Carpenter & Coustan criteria (OW/OB only) | 32 | Mean lean mass (SE), % | **61.46 ± 0.69** | **57.32 ± 1.56** | **p <0.05** |
| Liu et al. 2020 ^18^ | GDM, IADPSG criteria | 1318 | Mean skeletal muscle mass (SD), % | **43.0 (10.8)** | **40.0 (8.3)** | **<0.001** |
| Wang et al. 2019 ^5^ | GDM, IADPSG criteria | 2698 | Mean FFM (SD), % | **70.16 (5.03)** | **68.45 (4.81)** | **<0.001** |
| Subcutaneous fat (SF) | | | | | | |
| Yang et al. 2017 ^69^ | GDM, national diabetes data group criteria | 302 | Mean (SD) abdominal SF, cm | **1.7 (0.3)** | **2.7 (0.6)** | **<0.001** |
| Gur et al. 2014 ^47^ | GDM, patients with two of: FBG ≥105 mg/dL, 1h glucose ≥190 mg/dL, 2h glucose ≥165 mg/dL, and 3h glucose ≥145 mg/dL | 88 | Mean (SE) SF, mm | 12.3 (0.4) | 16.6 (1.3) | NR |
| Thaware et al. 2019 ^12^ | IADPSG/WHO criteria | 525 | Mean SFTD (SD), cm | 2.24 (1.08) | 22.7 (9.7) | 0.92 |
| Neck circumference (NC) |  |  |  |  |  |  |
| Egan et al. 2017 ^20^ | GDM in mid/late pregnancy (>20 weeks), IADPSG/WHO 2013 criteria (OB only) | 1023 | Mean (SD) NC, cm | 36.3 (2.1) | 36.3 (2.2) | NS (p not reported) |
| He et al. 2017 ^22^ | GDM, ADA criteria | 255 | Mean (SD) NC, cm | **33.89 (2.04)** | **35.20 (2.56)** | **0.003** |
| White et al. 2016 ^11^ | GDM, IADPSG criteria (OB only) | 1303 | Mean (SD) NC, cm | **36.3 (2.4)** | **37.4 (2.5)** | **<0.001** |
| Li et al., 2018 ^52^ | GDM, IADPSG criteria | 371 | Mean (SD) NC, cm | **33.5 (1.7)** | **34.3 (1.5)** | **<0.001** |
| Hancerliogullari et al. 2020 ^21^ | GDM, Carpenter & Coustan criteria | 525 | Mean NC (SD), cm | **36.21 (3.17)** | **37.14 (3.34)** | **0.042** |
| Arm circumference |  |  |  |  |  |  |
| White et al. 2016 ^11^ | GDM, IADPSG criteria (OB only) | 1303 | Median (IQR) mid-arm circumference, cm | **36 (34-38)** | **37 (35**, **40)** | **<0.001** |
| Wang et al. 2019 ^5^ | GDM, IADPSG Criteria | 2698 | Mean arm circumference (SD), cm | **26.81 (2.07)** | **27.64 (2.30)** | **<0.001** |
| Hip circumference (HC) | | | | | | |
| Minooee et al. 2017 ^9^ | GDM, WHO definition | 2458 | Mean HC (SD), cm | **103.5 (8.7)** | **106.34 (8.6)** | **<0.001** |
| Sina et al. 2015 ^10^ | GDM, ICD codes | 131 | Mean HC (SD), cm | **91.3 (11.9)** | **98.3 (16.3)** | **0.01** |
| Maitland et al. 2014 ^23^ | GDM, IADPSG criteria (OB only) | 106 | Mean HC (SD), cm | 122.9 (11.8) | 120.5 (9.2) | 0.27 |
| Alptekin et al. 2016 ^7^ | GDM, Carpenter & Coustan criteria | 227 | Mean HC (SD), cm | **99.4 (10.3)** | **105.8 (14.2)** | **0.01** |
| Thigh circumference | | | | | | |
| White et al. 2016 ^11^ | GDM, IADPSG criteria (OB only) | 1303 | Mean thigh circumference (SD), cm | 68.4 (6.3) | 68.9 (7.6) | 0.24 |
| Waist: height ratio (WHtR) |  |  |  |  |  |  |
| Sina et al. 2015 ^10^ | GDM, ICD 9 and 10-AM codes | 131 | Mean WHtR (SD) | **0.51 (0.08)** | **0.58 (0.12)** | **<0.001** |
| White et al. 2016 ^11^ | GDM, IADPSG criteria (OB only) | 1303 | Median WHtR (IQR) | **0.64 (0.60-0.68)** | **0.66 (0.63-0.72)** | **<0.001** |
| Skinfold thickness (SFT) |  |  |  |  |  |  |
| White et al. 2016 ^11^ | GDM, IADPSG criteria (OB only) | 1303 | Mean triceps SFT (SD), mm | **32.5 (8.7)** | **34.7 (9.6)** | **<0.001** |
| White et al. 2016 ^11^ | GDM, IADPSG criteria (OB only | 1303 | Median biceps SFT (IQR), mm | **20.3 (16-25)** | **21.8 (17-28)** | **<0.001** |
| White et al. 2016 ^11^ | GDM, IADPSG criteria (OB only | 1303 | Mean subscapular SFT (SD), mm | **34.4 (9.5)** | **38.3 (10.8)** | **<0.001** |
| White et al. 2016 ^11^ | GDM, IADPSG criteria (OB only | 1303 | Mean suprailiac SFT (SD), mm | **31.3 (10.9)** | **34.7 (11.1)** | **<0.001** |
| White et al. 2016 ^11^ | GDM, IADPSG criteria (OB only | 1303 | Mean sum of SFT (SD), mm | **119.5 (25.7)** | **131.2 (29.3)** | **<0.001** |
| Oriji et al. 2017 ^29^ | GDM, WHO criteria | 235 | Mean abdominal SFT (SE), mm | **32.30 (0.91)** | **38.20 (1.10)** | **0.001** |
| Neck: thigh ratio (NTR) |  |  |  |  |  |  |
| White et al. 2016 ^11^ | GDM, IADPSG criteria (OB only | 1303 | Mean NTR (SD) | **0.53 (0.05)** | **0.55 (0.07)** | **<0.001** |
| Waist: thigh ratio (WTH) |  |  |  |  |  |  |
| White et al. 2016 ^11^ | GDM, IADPSG criteria (OB only | 1303 | Median WTH (IQR) | **1.54 (1.43-1.66)** | **1.61 (1.5-1.71)** | **0.001** |
| Wrist circumference (WrC) |  |  |  |  |  |  |
| White et al. 2016 ^11^ | GDM, IADPSG criteria (OB only | 1303 | Median WrC (IQR) | **170 (161-180)** | **172 (165-180)** | **0.02** |

Abbreviation: ADA- American Diabetes Association; IADPSG- International Association of Diabetes and Pregnancy Study Groups; ICD- International Classification of Disease; CDA- Canadian Diabetes Association; DIPSI- Diabetes in Pregnancy Study Group of India; GTT- Glucose tolerance test; VF-visceral fat; VAT-visceral adipose tissue; BMI-body mass index; SD- Standard deviation; SE- Standard error; IQR- Inter-quartile range; NTR- Neck: thigh ratio; SFT-skinfold thickness; WTR-Waist: thigh ratio; WHtR- Waist: height ratio; HC-hip circumference; NC-neck circumference; FM-fat mass; FFM-fat-free mass; WHR-waist: hip ratio; WC-waist circumference; WrC- Wrist circumference; OW-overweight; OB-obese

* Blue highlighted results included in mean difference meta-analysis, bold data signify statistically significant results.

**Table S7. Insulin- and glucose-related outcomes (association data) for studies not reporting a GDM diagnosis outcome**

| **Paper** | **Outcome definition** | **Sample size** | | **Reference group** | **Comparison groups** | **Result *** | **Adjustments** |
| --- | --- | --- | --- | --- | --- | --- | --- |
| **Waist circumference** | | | | | | | |
| Brisson et al. 2013 ^38^ | HOMA-IR | 180 | | Continuous | 1: nulliparous  2: parous | 1: Adjusted Correlation (r) 0.20, p=0.06  2: Adjusted Correlation (r) 0.15, p=0.17 | Age, fasting glucose, first trimester BMI |
| Mostafavi et al. 2015 ^57^ | HOMA-IR | 80 | | <75cm | >90cm | **Mean HOMA-IR (SE): 3.8 (0.4); 11.2 (1.7), p<0.001** | None |
| Mostafavi et al. 2015 ^57^ | Insulin (U/L) | 80 | | <75cm | >90cm | **Mean insulin (SE): 17.8 (2.0); 48.0 (6.4), p<0.001** | None |
| Mostafavi et al. 2015 ^57^ | Glucose level | 80 | | <75cm | >90cm | **Mean glucose (SE) 1hr:1.25 (0.24); 1.69 (0.04), p<0.001**  **Mean glucose (SE) 2hr: 1.05 (0.03); 1.34 (0.04), p<0.001**  Mean glucose (SE) 3hr: 0.81(0.02); 0.90 (0.03), p=0.06 |  |
| Tomedi et al. 2014 ^65^ | Blood glucose (mg/dL) | 214 | | Continuous | Per SD (26.8cm) | Adjusted β coefficient 3.0 (95% CI -0.7, 6.8) | BMI, age, parity, race/ethnicity, maternal education, and family history of diabetes |
| **Subcutaneous fat (measured by ultrasound)** | | | | | | | |
| De Souza et al. 2014 ^42^ | HOMA-IR | 79 | | Continuous | Per 1 cm increase in SF | **Adjusted r^2^ 0.45 (95% CI 0.24, 0.55)** | Age, ethnicity, pre-pregnancy BMI, parity |
| De Souza et al. 2014 ^42^ | Insulin sensitivity index | 76 | | Continuous | Per 1 cm increase in SF | **Adjusted r^2^ 0.35 (95% CI 0.13, 0.46)** | Age, ethnicity, pre-pregnancy BMI, parity |
| Bartha et al. 2007 ^36^ | HOMA-IR | 30 | | Continuous | n/a | Correlation 0.17, p=0.36 | None |
| Bartha et al. 2007 ^36^ | Insulinaemia (uIU/mL) | 30 | | Continuous | n/a | Correlation 0.16, p=0.39 | None |
| Bartha et al. 2007 ^36^ | Glycemia (mg/dL) | 30 | | Continuous | n/a | Correlation 0.09, p=0.62 | None |
| Martin et al. 2009 ^54^ | Abnormal GCT: ≥7.8 mmol/L | 58 | | 2.18cm | 2.18cm | AOR 1.7 (95% CI 0.18, 16.5) | Maternal age, pre-pregnancy BMI |
| **Visceral fat (measured by ultrasound)** | | | | | | | |
| Bartha et al. 2007 ^36^ | Insulinaemia (uIU/mL) | 30 | | Continuous | n/a | **Correlation (r) 0.59, p=0.001** | None |
| Bartha et al. 2007 ^36^ | HOMA-IR | 30 | | Continuous | n/a | **Correlation (r) 0.59, p=0.001** | None |
| De Souza et al. 2014 ^42^ | Insulin sensitivity index | 76 | | Continuous | Per 1cm increase in visceral fat | Adjusted Beta co-efficient -0.09 (95% CI -0.18, 0.0) | Age, ethnicity, pre-pregnancy BMI, parity |
| De Souza et al. 2014 ^42^ | HOMA-IR | 79 | | Continuous | Per 1cm increase in visceral fat | Adjusted Beta co-efficient 0.09 (95% CI -0.01, 0.19) | Age, ethnicity, pre-pregnancy BMI, parity |
| Pontual et al. 2016 ^59^ | Fasting HOMA-IR | 334 | | Continuous | Per 1cm increase in visceral fat | **Adjusted Beta co-efficient 0.10 (95% CI 0.02, 0.18)** | Pre-pregnancy BMI, age and parity |
| Pontual et al. 2016 ^59^ | Fasting insulin | 334 | | Continuous | Per 1cm increase in visceral fat | Adjusted Beta co-efficient 0.28 (95% CI -0.04, 0.60) | Pre-pregnancy BMI, age and parity |
| Pontual, et al. 2016 ^59^ | Fasting glucose | 334 | | Continuous | Per 1cm increase in visceral fat | Adjusted Beta co-efficient 0.89 (95% CI 0.21, 1.58) | Pre-pregnancy BMI, age and parity |
| Pontual, et al. 2016 ^59^ | 1 hour glucose (OGTT) | 334 | | Continuous | Per 1cm increase in visceral fat | Adjusted Beta co-efficient 0.98 (95% CI -1.74, 3.71) | Pre-pregnancy BMI, age and parity |
| Pontual, et al. 2016 ^59^ | 2 hour glucose (OGTT) | 334 | | Continuous | Per 1cm increase in visceral fat | Adjusted Beta co-efficient 1.84 (95% CI -0.30, 3.98) | Pre-pregnancy BMI, age and parity |
| Bartha et al. 2007 ^36^ | Glycemia (mg/dL) | 30 | | Continuous | n/a | **Correlation (r) 0.37, p=0.04** | None |
| Martin et al. 2009 ^54^ | Abnormal GCT: ≥7.8 mmol/l | 58 | | <4.74 cm | ≥4.74 cm | **AOR 16.9 (95% CI 1.5, 194.6)** | Age, BMI |
| **Total adipose tissue** |  |  | |  |  |  |  |
| De Souza et al. 2014 ^42^ | Insulin sensitivity index | 76 | | Continuous | per 1cm increase | Adjusted r^2^ 0.41 (95%CI 0.19, 0.51) | Age, ethnicity, pre-pregnancy BMI, parity |
| De Souza et al. 2014 ^42^ | HOMA-IR | 79 | | Continuous | per 1cm increase | Adjusted r^2^ 0.46 (95%CI 0.25, 0.56) | Age, ethnicity, pre-pregnancy BMI, parity |
| **Visceral fat: subcutaneous fat ratio** | | | | | | | |
| Bartha et al. 2007 ^36^ | Insulinaemia (uIU/mL) | | 30 | Continuous | n/a | **Correlation (r) 0.47, p=0.008** | None |
| Bartha et al. 2007 ^36^ | HOMA-IR | | 30 | Continuous | n/a | **Correlation (r) 0.45, p=0.01** | None |
| **Skinfold Thickness** |  | |  |  |  |  |  |
| Tomedi et al. 2014 ^65^ | Blood glucose (mg/dL) | | 214 | Continuous | Per SD (8.6mm) increase bicep | **Adjusted β 4.3 (95% CI 0.2, 8.5)** | BMI, age, parity, race, education, family history of DM |
| Tomedi et al. 2014 ^65^ | Blood glucose (mg/dL) | | 214 | Continuous | Per SD (11.7mm) increase tricep | **Adjusted β 4.3 (95% CI 0.2, 8.5)** | BMI, age, parity, race, education, family history of DM |

Abbreviation: HOMA-IR- Homeostasis Model Assessment of insulin resistance; OGTT- Oral Glucose Tolerance Test; SE- standard error; WC-waist circumference; BMI-body mass index; IGT-Impaired glucose tolerance; GTT- Glucose tolerance test; FBS- Fasting blood sugar; GCT- Glucose challenge test.

* Bold data signify statistically significant results.

**Table S8A. Hypertensive disorders of pregnancy (association data)**

| **Paper** | **Outcome definition** | **Sample size** | **Reference group** | **Comparison groups** | **Result *** | **Adjustments** |
| --- | --- | --- | --- | --- | --- | --- |
| **Waist circumference (WC)** | | | | | | |
| Ebrahimi-Mameghani et al. 2013 ^30^ | Preeclampsia | 948 | <80 cm | 1: 80-88 cm  2: >88 cm | 1: AOR 2.29 (95% CI 0.87, 6.03)  **2: AOR 3.93 (95% CI 1.75, 8.80)** | Gestational hypertension, GDM, pregnancy delivery |
| Sattar et al. 2001 ^63^ | Preeclampsia | 1124 | <80 cm | ≥80 cm | **OR 2.7 (95% CI 1.1, 6.8)** | None |
| Wen et al. 2017 ^68^ | New onset hypertension | 1685 | ≤64 cm | 1: 65–69cm  2: 70–74cm  3: ≥75cm | **1: AOR 0.93 (95% CI 0.34, 2.52)**  **2: AOR 1.06 (95% CI 0.38, 2.91)**  **3: AOR 1.65 (95% CI 0.59, 4.59)** | Age, education, parity, BMI, serum folate, glucose, triglycerides, LDL cholesterol, HDL cholesterol |
| Ebrahimi-Mameghanib et al. 2013 ^30^ | Gestational hypertension (BP >140/90 after 20 weeks’ gestation without proteinurea) | 948 | <80 cm | 1: 80-88 cm  2: >88 cm | 1: AOR 2.02 (95% CI 0.74, 5.48)  **2: AOR 6.32 (95% CI 2.91, 13.71)** | Preeclampsia, gestational diabetes, pregnancy delivery |
| Sattar et al. 2001 ^63^ | Pregnancy-induced hypertension | 1124 | <80 cm | ≥80cm | **OR 1.8 (95% CI 1.1, 2.9)** | None |
| Gur et al 2014 ^47^ | Systolic BP (mm Hg) | 94 | Continuous | n/a | Pearson correlation (r) 0.192, p=0.6 | None |
| Gur et al. 2014 ^47^ | Diastolic BP (mmHg) | 94 | Continuous | n/a | P**earson correlation (r) 0.206, p=0.04** | None |
| Madhavan et al. 2008 ^4^ | Pregnancy-induced hypertension (140/90 no proteinuria) | 106 | Continuous | n/a | **Pearson correlation (r) 0.395, p<0.01** | None |
| Sina et al. 2014 ^28^ | Gestational hypertensive disorders** | 168 | Continuous | Per 1 SD increase in WC | **AOR 1.78 (95% CI 1.10, 2.89)** | Age, alcohol, smoking |
| Sharadha et al. 2016 ^64^ | Gestational hypertension and complications | 239 | 1: <80 cm (Asian-specific criteria)  2: <88 cm (WHO criteria) | 1: >80 cm  2: >88 cm | 1: Sensitivity 68.7%, Specificity 59.1%, PPV 10.8%, NPV 96.3%  2: Sensitivity 41.1%, Specificity 87.8%, PPV 20.5%, NPV 95.1% | None |
| **Mid-upper arm circumference (MUAC)** | | | | | | |
| Endeshaw et al. 2016 ^45^ | Preeclampsia (all) | 609 | MUAC <23 cm | 1: 23-24.9 cm  2: >25 cm | 1: AOR 1.63 (95% CI 0.84, 2.85)  **2: AOR 3.33 (95% CI 1.87, 5.79)** | Maternal age |
| Endeshaw et al. 2016 ^45^ | Preeclampsia (early onset <34 weeks) | 609 | MUAC <23 cm | 1: 23-24.9 cm  2: >25 cm | 1: AOR 1.37 (95% CI 0.54, 3.48)  2: AOR 1.98 (95% CI 0.79, 4.94) | Maternal age |
| Endeshaw et al. 2016 ^45^ | Preeclampsia (late onset ≥34 weeks) | 609 | MUAC <23 cm | 1: 23-24.9 cm  2: >25 cm | 1: AOR 1.24 (95% CI 0.69, 2.56)  **2: AOR 3.63 (95% CI 1.89, 6.97)** | Maternal age |
| **Hip circumference (HC)** | | | | | | |
| 316 Sina, et al. 2014 | Gestational hypertensive disorders ** | 168 | Continuous | Per 1 SD increase in HC | AOR 1.53 (95%CI 0.96, 2.52) | Age, alcohol, smoking |
| **Subcutaneous fat (measured by ultrasound)** | | | | | | |
| Kennedy et al. 2016 ^13^ | Pregnancy-induced hypertension (BP 140/90) | 1461 | Continuous | Per 5 mm increase in subcutaneous fat | AOR 1.03 (95% CI 0.89, 1.18) | BMI, maternal age, parity, smoking status |
| Gur et al. 2014 ^47^ | Systolic BP (mm Hg) | 94 | Continuous | n/a | Pearson correlation (r) 0.125, p=0.2 | None |
| Bartha et al. 2007 ^36^ | Systolic BP (mm Hg) | 30 | Continuous | n/a | Correlation (r) 0.27, p=0.14 | None |
| Gur et al. 2014 ^47^ | Diastolic BP (mmHg) | 94 | Continuous | n/a | Pearson correlation (r) 0.103, p=0.3 | None |
| Bartha et al. 2007 ^36^ | Diastolic BP (mmHg) | 30 | Continuous | n/a | **Correlation (r) 0.38, p=0.03** | None |
| **Visceral Fat (measured by ultrasound)** | | | | | | |
| Ray et al. 2017 ^61^ | Preeclampsia | 463 | <5.2 cm | ≥5.2 cm | ARR 3.4 (95% CI 0.9, 13.4) | Age, parity, chronic hypertension, BMI, ASA use |
| Ray et al. 2017 ^61^ | Preeclampsia (with preterm birth <37 weeks) | 463 | <5.2 cm | ≥5.2 cm | **ARR 16.9 (95% CI 1.2, 231.1)** | Age, parity, chronic hypertension, BMI, ASA use |
| Gur et al. 2014 ^47^ | Systolic BP (mm Hg) | 94 | Continuous | n/a | Correlation (r) 0.105, p=0.3 | None |
| Bartha et al. 2007 ^36^ | Systolic BP (mm Hg) | 30 | Continuous | n/a | Correlation (r) 0.36, p=0.05 | None |
| Gur et al. 2014 ^47^ | Diastolic BP (mmHg) | 94 | Continuous | n/a | **Correlation (r) 0.215, p=0.03** | None |
| Bartha et al. 2007 ^36^ | Diastolic BP (mmHg) | 30 | Continuous | n/a | **Correlation (r) 0.37, p=0.04** | None |
| **Waist: Hip ratio (WHR)** | | | | | | |
| Yamamoto et al. 2001 ^24^ | Preeclampsia | 224 | <0.90 | ≥0.90 | **OR 41.6 (95 % CI 11.90, 145.80)** | None |
| Taebi et al. 2015 ^25^ | Preeclampsia | 1000 | ≤0.85 | >0.85 | **OR 2.43 (95% CI 1.27, 4.67)** | None |
| Khare et al. 2017 ^3^ | Gestational hypertension/pre-eclampsia | 120 | ≤0.85 | >0.85 | OR 2.03 (95% CI 0.10, 40.50) | None |
| Madhavan et al. 2008 ^4^ | Pregnancy-induced hypertension (140/90 no proteinuria) | 106 | ≤0.85 | >0.85 | OR 2.29 (95% CI 0.37, 14.30) | None |
| Sina et al. 2014 ^28^ | Gestational hypertensive disorders ** | 107 | Continuous | 1 SD increase in WHR | AOR 1.65 (95% CI 0.80, 3.39) | Age, parity, smoking, drinking |
| **Waist: Height ratio (WHtR)** | | | | | | |
| Sina et al. 2014 ^28^ | Gestational hypertensive disorders ** | 168 | Continuous | Per 1 SD increase in WHtR | AOR 1.44 (95% CI 0.83, 2.51) | Age, alcohol, smoking |
| **Fat mass** | | | | | | |
| Wang et al. 2015 ^67^ | Preeclampsia | 463 | <30 kg | 1: ≥30 kg  2: ≥35 kg  3: ≥40 kg | **1: AOR 1.34 (95% CI 1.01, 2.68)**  **2: AOR 1.98 (95% CI 1.16, 4.47)**  **3: AOR 6.84 (95% CI 4.15, 41.6)** | Age, education, gestational age, employ, stay up late, diet, smoking, alcohol, BMI, PBF, FFM |
| Sween et al. 2015 ^26^ | Preeclampsia (all patients) | 373 | continuous | Per % increase in body fat | AOR 1.01 (95% CI 0.97, 1.05) | Age, race, and smoking status. |
| Sween et al. 2015 ^26^ | Preeclampsia (obese only) | 27 | Continuous | Per % increase in body fat | **AOR 1.13 (95% CI 1.01, 1.26)** | Age, race, and smoking status |
| **Ratio of Visceral fat thickness: Subcutaneous fat thickness** | | | | | | |
| Bartha et al. 2007 ^36^ | Systolic BP (mmHg) | 30 | Continuous | n/a | Correlation (r) 0.15, p=0.4 | None |
| Bartha et al. 2007 ^36^ | Diastolic BP (mmHg) | 30 | Continuous | n/a | Correlation (r) 0.12, p=0.54 | None |
| **Fat-Free Mass Index** | | | | | | |
| Wang et al. 2015 ^67^ | Preeclampsia | 463 | 13-14.9 kg/m^2^ | 1: 15-16.9 kg/m^2^  2: ≥17 kg/m^2^ | 1. OR 1.44 (95% CI 0.79, 2.61)  2. OR 0.48 (95% CI 0.53, 4.32) | None |

Abbreviation: WC-waist circumference; MUAC- Mid-upper arm circumference; BP: Blood pressure, OW- Overweight, OB- Obese, GH- Gestational hypertension; SD- standard deviation; BMI-body mass index; ASA- acetylsalicylic acid; HC-hip circumference; WHR- Waist- Hip ratio; WHtR- Waist: Height ratio; FM-fat mass;

* Green highlighted results included in categorical meta-analysis, bold data signify statistically significant results.

**Definition of gestational hypertensive disorders (GH, PE, eclampsia, pre-existing hypertension with superimposed gestational PE-eclampsia

**Table S8B. Hypertensive disorders of pregnancy (case control data reported)**

| **Paper** | **Outcome definition** | **Sample size** | **Data reported** | **Controls**  **(no hypertensive disorder)** | **Cases**  **(hypertensive disorder)** | **Significance, p value** |
| --- | --- | --- | --- | --- | --- | --- |
| **Waist circumference (WC)** | | | | | | |
| Taebi et al. 2015 ^25^ | Preeclampsia | 1000 | Mean WC (SD), cm | **79.85 (10.32)** | **86.44 (8.47)** | **0.0001** |
| Sween et al. 2015 ^26^ | Preeclampsia | 373 | Mean WC (SD), mm | 1002 (168) | 1011 (175) | 0.78 |
| Kausar et al. 2013 ^27^ | Gestational hypertension/pre-eclampsia | 150 | Mean WC(SD), cm | **69.43 (3.95)** | **82.27 (6.96)** | **<0.001** |
| Sina et al. 2014 ^28^ | Gestational hypertensive disorders ** | 168 | Mean WC (SD), cm | **78.6 (15.7)** | **88.5 (19.1)** | **0.013** |
| Vieira et al. 2017 ^66^ | Preeclampsia (OW only) | 3106 | Median WC (IQR), cm | 77 (74–82) | 78 (74–82) | 0.45 |
| Vieira et al. 2017 ^66^ | Preeclampsia (OB only) | 834 | Median WC (IQR), cm | 101 (95–108) | 102 (96–111) | 0.1 |
| Sattar et al. 2001 ^63^ | Preeclampsia | 1124 | Median WC (IQR), cm | **77 (72-83)** | **80 (78-91)** | **0.02** |
| Sattar et al. 2001 ^63^ | Pregnancy-induced hypertension | 1124 | Median WC (IQR), cm | **77 (72-83)** | **81 (76-90)** | **0.002** |
| Piuri et al. 2016 ^58^ | HDP-AGA | 125 | Median WC (IQR), cm | **88 (84–94)** | **98 (97–106)** | **<0.01** |
| **Arm circumference** | | | | | | |
| Vieira et al. 2017 ^66^ | Preeclampsia (OW only) | 3106 | Median (IQR) arm circumference, cm | **26 (24–27)** | **27 (25–28)** | **0.008** |
| Vieira et al. 2017 ^66^ | Preeclampsia (OB only) | 834 | Median (IQR) arm circumference, cm | 34 (31–36) | 34 (32–38) | 0.16 |
| **Hip circumference (HC)** | | | | | | |
| Taebi et al. 2015 ^25^ | Preeclampsia | 1000 | Mean HC (SD), cm | **100.46 (9.15)** | **105.46 (6.78)** | **0.0001** |
| Vieira et al. 2017 ^66^ | Preeclampsia (Normal BMI) | 3106 | Median HC (IQR), cm | 95 (91–99) | 97 (92–100) | 0.08 |
| Vieira et al. 2017 ^66^ | Preeclampsia (OB only) | 834 | Median HC (IQR), cm | 118 (112–125) | 119 (113–130) | 0.07 |
| Sina et al. 2014 ^28^ | Gestational hypertensive disorders ** | 168 | Mean HC (SD), cm | **89.2 (16.6)** | **98.2 (18.9)** | **0.029** |
| **Waist: Hip ratio (WHR)** | | | | | | |
| Yamamoto et al. 2001 ^24^ | Preeclampsia | 224 | Mean WHR (SD) | **0.76 (0.06)** | **0.85 (0.09)** | **<0.0001** |
| Taebi et al. 2015 ^25^ | Preeclampsia | 1000 | Mean WHR (SD) | **0.79 (0.06)** | **0.82 (0.05)** | **0.016** |
| Sween et al. 2015 ^26^ | Preeclampsia (OW+OB only) | 373 | Mean WHR (SD) | 0.86 (0.07) | 0.87 (0.08) | 0.68 |
| Kausar et al. 2013 ^27^ | Gestational hypertension/pre-eclampsia | 150 | Mean WHR (SD) | **0.79 (0.03)** | **0.85 (0.03)** | **<0.001** |
| Sina et al. 2014 ^28^ | Gestational hypertensive disorders ** | 107 | Mean WHR (SD) | **0.89 (0.06)** | **0.92 (0.05)** | **0.1** |
| **Waist: Height ratio (WHtR)** | | | | | |  |
| Sina et al. 2014 ^28^ | Gestational hypertensive disorders ** | 168 | Mean WHtR (SD) | 0.88 (0.07) | 0.90 (0.06) | 0.257 |
| Kausar et al. 2013 ^27^ | Gestational hypertension/pre-eclampsia | 150 | Mean WHtR (SD) | **0.45 (0.03)** | **0.55 (0.07)** | **<0.001** |
| **Fat mass (FM)** | | | | | | |
| Yeboah et al. 2017 ^70^ | Preeclampsia | 312 | Mean body fat (SD), % | **29.6 (7.0)** | **41.6 (2.7)** | **<0.0001** |
| Sween et al. 2015 ^26^ | Preeclampsia | 373 | Mean body fat (SD), % | **45.8 (10.1)** | **46.9 (11.3)** | **0.57** |
| Piuri et al. 2016 ^58^ | HDP-AGA | 125 | Median FM (IQR), kg | **15.2 (12.1–19.1)** | **26.0 (21.2–29.7)** | **<0.01** |
| **Fat Free Mass (FFM)/other mass** | | | | | | |
| Yeboah et al. 2017 ^70^ | Preeclampsia | 312 | Mean muscle mass (SD), % | **30.9 (3.5)** | **24.8 (1.3)** | **<0.0001** |
| Yeboah et al. 2017 ^70^ | Preeclampsia | 312 | Mean bone density (SD) | 2.1 (0.3) | 2.2 (0.1) | 0.167 |
| Yeboah et al. 2017 ^70^ | Preeclampsia | 312 | Mean water mass (SD), % | **49.6 (6.7)** | **38.2 (2.9)** | **<0.0001** |
| Piuri et al. 2016 ^58^ | HDP-AGA | 125 | Median FFM (IQR), kg | **43.4 (41.4–46.7)** | **47.9 (45.0–56.4)** | **<0.05** |
| Piuri et al. 2016 ^58^ | HDP-AGA | 125 | Median total body water (IQR), kg | **30.3 (28.9–32.8)** | **33.6 (32.3–40.7)** | **<0.05** |
| **Skinfold thickness (SFT)** | | | | | | |
| Piuri et al. 2016 ^58^ | HDP-AGA | 125 | Median total SFT (triceps, biceps, subscapular) (IQR), cm | **37 (32-44)** | **63 (52–76)** | **<0.001** |

Abbreviation: BP - Blood pressure, OW - Overweight, OB - Obese, GH - Gestational hypertension; SD - standard deviation; WC - waist circumference, HC - hip circumference; WHR - Waist hip ratio; WHtR- Waist height ratio; FM - fat mass; FFM - fat free mass; BMI - body mass index; HDP-AGA - Hypertensive appropriate gestational age: defined as (hypertensive mothers BP >140/90 after 20 weeks' gestation with appropriate weight for gestational age new-borns >the 10th centile)

* Blue highlighted results included in mean difference meta-analysis, bold data signify statistically significant results.

**Gestational hypertensive disorders is defined as GH, PE, eclampsia, pre-existing hypertension with superimposed gestational PE-eclampsia

**Table S9: Delivery-related outcomes**

| **Paper** | **Outcome definition** | **Sample size** | **Reference group** | **Comparison groups** | **Result** | **Adjustments** |
| --- | --- | --- | --- | --- | --- | --- |
| **Waist circumference** | | | | | | |
| Gao et al. 2017 ^46^ | Primary caesarean section | 919 | <80 cm | ≥80 cm | **AOR 1.71 (95% CI 1.11, 2.63)** | Street, maternal/paternal age/education/BMI, active/passive smoking, alcohol, family income, parity, infant gender, gestational age |
| Migda et al. 2016 ^56^ | Caesarean delivery | 157 | Continuous | n/a | **AUROC 0.706, p value NR (authors classify >0.7 as predictive)** | None |
| Madhavan et al. 2008 ^4^ | Induction | 106 | Continuous | n/a | **Correlation (r) 0.221, p<0.05** | None |
| Madhavan et al. 2008 ^4^ | Abnormal delivery^a^ | 106 | Continuous | n/a | **Correlation (r) 0.25, p<0.01** | None |
| **Waist to hip ratio (WHR)** | | | | | | |
| McDonnold et al. 2016 ^55^ | Caesarean delivery | 2276 | <0.80 | 1: 0.80 - 0.84  2: ≥ 0.85 | **1: AOR 1.43 (95% CI 1.08, 1.89)**  **2: AOR 1.74 (95% CI 1.35, 2.25)** | Age, gestational age at enrolment, years of schooling, race, alcohol, smoking status |
| Migda et al. 2016 ^56^ | Caesarean delivery | 157 | Continuous | n/a | **AUROC 0.732 (authors define >0.7 as predictive)** | None |
| Madhavan et al. 2008 ^4^ | Abnormal delivery * | 106 | ≤0.85 | >0.85 | **OR 8.35 (95% CI 2.79, 25.0)** | None |
| Madhavan et al. 2008 ^4^ | Induction | 106 | ≤0.85 | >0.85 | **OR 4.06 (95% CI 1.70, 9.66)** | None |
| Salem et al. 2012 ^62^ | Non-spontaneous birth | 3083 | WHR Q1 (0.67-0.69) | 1: WHR Q2 (0.70-0.72)  2: WHR Q3 (0.74-0.76)  3: WHR Q4 (0.78-0.85) | 1: OR 0.94 (95% CI 0.74, 1.19)  2: OR 1.11 (95% CI 0.88, 1.40)  3: OR 1.02 (95% CI 0.80, 1.30) | None |
| **Waist: hip ratio (WHR) and body mass index (BMI)** | | | | | | |
| McDonnold et al. 2016 ^55^ | Caesarean delivery | 2276 | Either WHR <0.85 or BMI <30 | Both WHR ≥ 0.85 and BMI ≥ 30 | **OR 2.48 (95%CI 1.88, 3.28)** | None |
| **Subcutaneous fat (SF) (measured by ultrasound)** | | | | | | |
| Kennedy et al. 2016 ^13^ | Caesarean delivery | 1461 | Continuous | Per 5 mm increase in SF | AOR 1.09 (95% CI 0.99, 1.2) | BMI, maternal age, parity, smoking status |
| Kennedy et al. 2016 ^13^ | Induction of labour | 1461 | Continuous | Per 5 mm increase in SF | AOR 1.07 (95% CI 0.96, 1.16) | BMI, maternal age, parity, smoking status |
| Kennedy et al. 2016 ^13^ | Assisted delivery | 1461 | Continuous | Per 5 mm increase in SF | AOR 0.94 (95% CI 0.78, 1.13) | BMI, maternal age, parity, smoking status |
| Suresh et al. 2012 ^14^ | Caesarean delivery | 1200 | Continuous | Per 5 mm increase in SF | **AOR 1.05 (95%CI 1.03, 1.07)** | Maternal age |

 Abbreviation: Q – Quartile; BMI-body mass index; WHR-waist: hip ratio; SF-subcutaneous fat; AUROC- area under the receiver operating characteristic

* Abnormal delivery including instrumental deliveries and lower segment caesarean sections, bold data signify statistically significant results

**Table S10. Maternal lipids**

| **Paper** | **Outcome definition** | **Sample size** | **Reference group** | **Comparison groups** | **Result** | **Adjustments** |
| --- | --- | --- | --- | --- | --- | --- |
| **Waist circumference** | | | | | | |
| Mostafavi et al. 2015 ^57^ | Triglycerides (g/L): 3h after OGTT | 80 | <75cm | >90cm | **Mean (SE) g/L: 1.61 (0.07); 2 (0.08), p<0.001** | None |
| Mostafavi et al. 2015 ^57^ | Triglycerides (g/L) before OGTT | 80 | <75cm | >90cm | **Mean (SE) g/L: 1.73 (0.08); 1.99 (0.07), p<0.001** | None |
| Gur et al. 2014 ^47^ | Triglycerides (mg/dL) | 94 | Continuous | n/a | **Pearson correlation (r) 0.327, p=0.01** | None |
| Gur et al. 2014 ^47^ | HDL-C (mg/dL) | 94 | Continuous | n/a | Pearson correlation (r) 0.153, p=0.1 | None |
| Gur et al. 2014 ^47^ | LDL-C (mg/dL) | 94 | Continuous | n/a | Pearson correlation (r) 0.122, p=0.2 | None |
| Gur et al. 2014 ^47^ | Total cholesterol (mg/dL) | 94 | Continuous | n/a | Pearson correlation (r) 0.052, p=0.6 | None |
| **Neck circumference** | | | | | | |
| KhushBakht et al. 2018 ^50^ | Triglycerides (mg/dL) | 90 | Continuous | n/a | Adjusted Correlation Coefficient (r) 0.130, p=0.059 | Age |
| KhushBakht et al. 2018 ^50^ | Total cholesterol (mg/dL) | 90 | Continuous | n/a | Adjusted Correlation Coefficient (r) 0.101, p=0.218 | Age |
| **Subcutaneous fat (measured by ultrasound)** | | | | | | |
| Bartha et al. 2007 ^36^ | Triglycerides (mg/dL) | 30 | Continuous | n/a | Correlation (r) 0.10, p=0.58 | None |
| Gur et al. 2014 ^47^ | Triglycerides (mg/dL) | 94 | Continuous | n/a | **Pearson correlation (r) 0.26, p=0.01** | None |
| Bartha et al. 2007 ^36^ | HDL-C (mg/dL) | 30 | Continuous | n/a | Correlation (r) -0.32, p=0.08 | None |
| Gur et al. 2014 ^47^ | HDL-C (mg/dL) | 94 | Continuous | n/a | Pearson correlation (r) –0.02, p=0.9 | None |
| Bartha et al. 2007 ^36^ | LDL-C (mg/dL) | 30 | Continuous | n/a | Correlation (r) -0.07, p=0.70 | None |
| Gur et al. 2014 ^47^ | LDL-C (mg/dL) | 94 | Continuous | n/a | Pearson correlation (r) 0.031, p=0.7 | None |
| Bartha et al. 2007 ^36^ | Total cholesterol/HDL-C | 30 | Continuous | n/a | Correlation 0.09 (r), p=0.62 | None |
| Bartha et al. 2007 ^36^ | Total cholesterol (mg/dL) | 30 | Continuous | n/a | Correlation -0.27 (r), p=0.19 | None |
| Gur et al. 2014 ^47^ | Total cholesterol (mg/dL) | 94 | Continuous | n/a | Pearson correlation (r) 0.071, p=0.4 | None |
| Bartha et al. 2007 ^36^ | Free fatty acids (g/L) | 30 | Continuous | n/a | Correlation (r) -0.08, p=0.65 | None |
| **Visceral fat (measured by ultrasound)** | | | | | | |
| Bartha et al. 2007 ^36^ | Triglycerides (mg/dL) | 30 | Continuous | n/a | **Correlation (r) 0.58, p=0.001** | None |
| Gur et al. 2014 ^47^ | Triglycerides (mg/dL) | 94 | Continuous | n/a | **Pearson correlation (r) 0.266, p=0.01** | None |
| Pontual et al. 2016 ^59^ | Triglycerides (mg/dL) | 334 | Continuous | n/a | Adjusted β co-efficient 2.30 (95% CI -1.42, 6.02) | Pre-pregnancy BMI, age, parity |
| Bartha et al. 2007 ^36^ | HDL-C (mg/dL) | 30 | Continuous | n/a | **Correlation (r) -0.39, p=0.03** | None |
| Gur et al. 2014 ^47^ | HDL-C (mg/dL) | 94 | Continuous | n/a | **Pearson correlation (r) –0.205, p=0.04** | None |
| Bartha et al. 2007 ^36^ | LDL-C (mg/dL) | 30 | Continuous | n/a | Correlation (r) 0.34, p=0.07 | None |
| Gur et al. 2014 ^47^ | LDL-C (mg/dL) | 94 | Continuous | n/a | Pearson correlation (r) 0.051, p=0.6 | None |
| Pontual et al. 2016 ^59^ | VLDL-C (mg/dL) | 334 | Continuous | n/a | Adjusted β co-efficient 0.50 (95% CI -0.22, 1.22) | Pre-pregnancy BMI, age, parity |
| Bartha et al. 2007 ^36^ | Total cholesterol/HDL-C | 30 | Continuous | n/a | **Correlation (r) 0.54, p=0.002** | None |
| Bartha et al. 2007 ^36^ | Total cholesterol (mg/dL) | 30 | Continuous | n/a | Correlation (r) 0.06, p=0.75 | None |
| Gur et al. 2014 ^47^ | Total cholesterol (mg/dL) | 94 | Continuous | n/a | Pearson correlation (r) 0.03, p=0.7 | None |
| Bartha et al. 2007 ^36^ | Free fatty acids (g/L) | 30 | Continuous | n/a | Correlation (r) 0.16, p=0.39 | None |
| **Waist: Hip ratio** | | | | | | |
| Khare et al. 2017 ^3^ | Triglycerides (mg/dL) | 120 | Continuous | n/a | Correlation co-efficient (r) 0.096, p=0.296 | None |
| Khare et al. 2017 ^3^ | HDL-C (mg/dL) | 120 | Continuous | n/a | Correlation co-efficient (r) 0.003, p=0.971 | None |
| Khare et al. 2017 ^3^ | LDL-C (mg/dL) | 120 | Continuous | n/a | Correlation co-efficient (r) 0.073, p=0.43 | None |
| Khare et al. 2017 ^3^ | VLDL-C (mg/dL) | 120 | Continuous | n/a | **Correlation co-efficient (r) 0.198, p=0.031** | None |
| Khare et al. 2017 ^3^ | Total cholesterol (mg/dL) | 120 | Continuous | n/a | Correlation co-efficient (r) 0.043, p=0.642 | None |
| Khare et al. 2017 ^3^ | TG/LDL ratio | 120 | Continuous | n/a | Correlation co-efficient (r) 0.005, p=0.96 | None |
| Khare et al. 2017 ^3^ | Cholesterol/HDL ratio | 120 | Continuous | n/a | Correlation co-efficient (r) 0.015, p=0.875 | None |
| **Ratio of Visceral fat thickness: Subcutaneous fat thickness** | | | | | | |
| Bartha et al. 2007 ^36^ | Triglycerides (mg/dL) | 30 | Continuous | n/a | **Correlation (r) 0.56, p=0.001** | None |
| Bartha et al. 2007 ^36^ | HDL-C (mg/dL) | 30 | Continuous | n/a | Correlation (r) -0.20, p= 0.28 | None |
| Bartha et al. 2007 ^36^ | LDL-C (mg/dL) | 30 | Continuous | n/a | Correlation (r) 0.31, p=0.09 | None |
| Bartha et al. 2007 ^36^ | Total cholesterol/HDL-C | 30 | Continuous | n/a | **Correlation (r) 0.44, p=0.01** | None |
| Bartha et al. 2007 ^36^ | Total cholesterol (mg/dL) | 30 | Continuous | n/a | Correlation (r) 0.2, p=0.29 | None |
| Bartha et al. 2007 ^36^ | Free fatty acids | 30 | Continuous | n/a | Correlation (r) 0.24, p=0.23 | None |

Abbreviation: OGTT- oral glucose tolerance test; HDL-C- high-density lipoprotein cholesterol, LDL-C- low-density lipoprotein cholesterol; VLDL-C- very low-density lipoprotein cholesterol; TG- triglyceride; SE- standard error; BMI-body mass index. Bold data signify statistically significant results.

**Table S11. Metabolic syndrome**

| **Paper** | **Outcome definition** | **Sample size** | **Data reported** | **Controls (no MS)** | **Cases (MS)** | **Significance, p value** |
| --- | --- | --- | --- | --- | --- | --- |
| **Waist circumference (WC)** | | | | | | |
| Lima et al. 2019 ^53^ | NCEP/ATP III criteria) at 16 weeks’ gestation | 200 | Mean WC (SD), cm | **88.0 (9.6)** | **98.9 (12.4)** | **<0.001** |
| Lima et al. 2019 ^53^ | NCEP/ATP III criteria) immediate postpartum | 200 | Mean WC (SD), cm | **91.9 (9.5)** | **98.6 (8.5)** | **<0.001** |
| Gur et al, 2014 ^47^ | Inter-national Diabetes Federation 2005 criteria | 94 | Mean WC, mm | **44.2** | **77.9** | **<0.001** |
| **Arm circumference (AC)** | | | | | | |
| Lima et al. 2019 ^53^ | NCEP/ATP III criteria at 16 weeks’ gestation | 200 | Mean AC (SD), cm | **28.0 (3.4)** | **34.1 (6.3)** | **<0.001** |
| Lima et al. 2019 ^53^ | NCEP/ATP III criteria immediate postpartum | 200 | Mean AC (SD), cm | 28.5 (3.1) | 29.4 (2.3) | 0.13 |
| **Leg circumference** | | | | | | |
| Lima et al. 2019 ^53^ | NCEP/ATP III criteria at 16 weeks’ gestation | 200 | Mean thigh circ. (SD), cm | 29.6 (7.7) | 30.8 (11.1) | 0.72 |
| Lima et al. 2019 ^53^ | NCEP/ATP III criteria immediate postpartum | 200 | Mean thigh circ. (SD), cm | 38.2 (9.2) | 41.7 (10.1) | 0.21 |
| **Subcutaneous fat (SF) (measured by ultrasound)** | | | | | | |
| Lima et al. 2019 ^53^ | NCEP/ATP III criteria at 16 weeks’ gestation | 200 | Mean SF (SD), cm | 2.3 (0.8) | 2.9 (0.8) | 0.07 |
| Lima et al. 2019 ^53^ | NCEP/ATP III criteria immediate postpartum | 200 | Mean SF (SD), cm | **2.4 (0.8)** | **3.0 (1.0)** | **0.01** |
| Gur et al. 2014 ^47^ | Inter-national Diabetes Federation 2005 criteria | 94 | Mean SF, mm | **45.4** | **66.5** | **0.02** |
| **Visceral fat (VF) (measured by ultrasound)** | | | | | | |
| Lima et al. 2019 ^53^ | NCEP/ATP III criteria at 16 weeks' gestation | 200 | Mean VF (SD), cm | 5.2 (1.3) | 5.9 (1.2) | 0.2 |
| Lima et al. 2019 ^53^ | NCEP/ATP III criteria immediate postpartum | 200 | Mean VF (SD), cm | 5.6 (1.5) | 5.5 (1.9) | 0.83 |
| Gur et al. 2014 ^47^ | Inter-national Diabetes Federation 2005 criteria | 94 | Mean VF, mm | **45.4** | **66.8** | **0.02** |
| **Skinfold thickness (SFT)** | | | | | | |
| Lima et al. 2019 ^53^ | NCEP/ATP III criteria at 16 weeks' gestation | 200 | Mean triceps SFT (SD), cm | **21.3 (5.6)** | **28.1 (11.5)** | **0.001** |
| Lima et al. 2019 ^53^ | NCEP/ATP III criteria immediate postpartum | 200 | Mean triceps SFT (SD), cm | **22.6 (6.0)** | **26.2 (7.8)** | **0.03** |
| Lima et al. 2019 ^53^ | NCEP/ATP III criteria at 16 weeks' gestation | 200 | Mean suprailiac SFT (SD), cm | **22.8 (8.9)** | **30.3 (16.4)** | **0.05** |
| Lima et al. 2019 ^53^ | NCEP/ATP III criteria immediate postpartum | 200 | mean suprailiac SFT (SD), cm | **22.6 (6.0)** | **26.2 (7.8)** | **0.03** |

Abbreviation: NCEP/ATP- National Cholesterol Education Program/Adult Treatment Panel; SD- standard deviation; VF-visceral fat; SFT- Skinfold thickness; SF- Subcutaneous fat; WC-waist circumference; AC-arm circumference. Bold data signify statistically significant results.

**Table S12A. Composite adverse pregnancy outcomes (association data reported)**

| **Paper** | **Outcome definition** | **Sample size** | **Reference group** | **Comparison groups** | **Result** | **Adjustments** |
| --- | --- | --- | --- | --- | --- | --- |
| **Waist circumference** | | | | | | |
| Gao et al. 2017 ^46^ | One or more of the adverse pregnancy outcomes (LGA, GDM, P-CS) | 919 | <80cm | ≥80cm | **AOR 1.98 (95% CI 1.30, 3.01)** | Street, maternal/paternal age, education, BMI, active/passive smoking, alcohol, family income, parity, infant gender and gestational age |
| **Combined waist circumference (WC) and body mass index (BMI)** | | | | | | |
| Gao et al. 2017 ^46^ | One or more of the adverse pregnancy outcomes (LGA, GDM, P-CS) | 919 | Pre-pregnancy BMI≤24 kg/m^2^ and WC<80 cm | 1. BMI≤24kg/m^2^ and WC<80 cm  2. BMI≤24kg/m^2^ and WC≥80cm  3. BMI≥24 kg/m^2^ and WC≥80cm | **1. AOR 2.10 (95%CI 1.14, 3.88)**  **2. AOR 2.25 (95%CI 1.36, 3.72)**  **3. AOR 3.96 (95%CI 2.40, 6.54)** | Street, maternal/paternal age, education, BMI, active/passive smoking, alcohol, family income, parity, infant gender and gestational age |
| **Waist: Hip ratio** | | | | | | |
| Khare et al. 2017 ^3^ | Total antenatal complications | 120 | ≤0.80 | >0.80 | OR 1.43 (95% CI 0.29, 6.97) | None |
| **Subcutaneous fat (SF) (measured by ultrasound)** | | | | | | |
| Suresh et al. 2012 ^14^ | Cumulative adverse pregnancy outcomes | 1200 | continuous | Per 5 mm increase in SF | **AOR 1.04 (95%CI 1.01, 1.06)** | Maternal age |
| **Fat mass (FM)** | | | | | | |
| Gagliardi et al.  2016 ^33^ | Complicated pregnancy (Preeclampsia and/or fetal growth restriction) | 150 | FM (%) >20 (presumed) | ≤20 | **AOR 11.58 (95%CI 1.96, 67.85)** | Total vascular resistance, inotropy index, time flow correct, total body water, extracellular body water, intracellular body water, combined screening |
| Gagliardi et al.  2016 ^33^ | Complicated pregnancy (Preeclampsia and/or fetal growth restriction) | 150 | FM (%), continuous | n/a | OR 1.00 (95%CI 0.92, 1.09) | None |

Abbreviation: GDM- gestational DM; LGA: large for gestational age, P-CS- primary caesarean section; FM-fat mass; SF- Subcutaneous fat; WC-waist circumference; BMI-body mass index. Bold data signify statistically significant results.

**Table S12B. Composite adverse pregnancy outcomes (case control data reported)**

| **Study** | **Outcome definition** | **Sample size** | **Data reported** | **Controls (no composite outcome)** | **Cases (composite outcome)** | **Significance, p value** |
| --- | --- | --- | --- | --- | --- | --- |
| **Fat mass (FM)** |  |  |  |  |  |  |
| Gagliardi et al.  2016 ^33^ | Complicated pregnancy (preeclampsia and FGR) | 150 | Mean FM (SD) % | 22.6 (7.10) | 22.6 (5.40) | 0.915 |

Abbreviation: SD- standard deviation; FM-fat mass; FGR – fetal growth restriction. Bold data signify statistically significant results.

**Table S13. Gestational weight gain**

| **Paper** | **Outcome definition** | **Sample size** | **Fat mass reference group** | **Fat mass comparison groups** | **Result** | **Adjustments** |
| --- | --- | --- | --- | --- | --- | --- |
| **Fat mass (FM)** |  |  |  |  |  |  |
| Lacroix et al. 2016 ^51^ | Gestational weight gain | 675 | Continuous | n/a | **Pearson correlations (r) -0.24, p<0.0001** | None |

Abbreviation: FM-fat mass. Bold data signify statistically significant results.

**Supporting information references**

1. Basraon SK, Mele L, Myatt L, et al. Relationship of Early Pregnancy Waist-to-Hip Ratio versus Body Mass Index with Gestational Diabetes Mellitus and Insulin Resistance. *Am J Perinatol*. Jan 2016;33(1):114-21. doi:10.1055/s-0035-1562928

2. Zhu Y, Hedderson MM, Quesenberry CP, Feng J, Ferrara A. Central Obesity Increases the Risk of Gestational Diabetes Partially Through Increasing Insulin Resistance. *Obesity (Silver Spring)*. Jan 2019;27(1):152-160. doi:10.1002/oby.22339

3. Khare D, Modi J. Waist hip ratio in early pregnancy as a clinical indicator of serum lipid levels and predictor of pregnancy complications. *International Journal of Reproduction, Contraception, Obstetrics and Gynecology*. 2016:1709-1713. doi:10.18203/2320-1770.ijrcog20161444

4. Madhavan A, Beena Kumari R, Sanal MG. A pilot study on the usefulness of body mass index and waist hip ratio as a predictive tool for gestational diabetes in Asian Indians. *Gynecol Endocrinol*. Dec 2008;24(12):701-7. doi:10.1080/09513590802444134

5. Wang Y, Luo BR. The association of body composition with the risk of gestational diabetes mellitus in Chinese pregnant women: A case-control study. *Medicine (Baltimore)*. Oct 2019;98(42):e17576. doi:10.1097/MD.0000000000017576

6. Zhang S, Folsom AR, Flack JM, Liu K. Body fat distribution before pregnancy and gestational diabetes: findings from coronary artery risk development in young adults (CARDIA) study. *BMJ*. Oct 28 1995;311(7013):1139-40. doi:10.1136/bmj.311.7013.1139

7. Alptekin H, Cizmecioglu A, Isik H, Cengiz T, Yildiz M, Iyisoy MS. Predicting gestational diabetes mellitus during the first trimester using anthropometric measurements and HOMA-IR. *J Endocrinol Invest*. May 2016;39(5):577-83. doi:10.1007/s40618-015-0427-z

8. Balani J, Hyer S, Johnson A, Shehata H. The importance of visceral fat mass in obese pregnant women and relation with pregnancy outcomes. *Obstet Med*. Mar 2014;7(1):22-5. doi:10.1177/1753495X13495192

9. Minooee S, Ramezani Tehrani F, Rahmati M, Mansournia MA, Azizi F. Diabetes incidence and influencing factors in women with and without gestational diabetes mellitus: A 15year population-based follow-up cohort study. *Diabetes Res Clin Pract*. Jun 2017;128:24-31. doi:10.1016/j.diabres.2017.04.003

10. Sina M, Hoy WE, Callaway L, Wang Z. The associations of anthropometric measurements with subsequent gestational diabetes in Aboriginal women. *Obes Res Clin Pract*. Sep-Oct 2015;9(5):499-506. doi:10.1016/j.orcp.2015.02.005

11. White SL, Lawlor DA, Briley AL, et al. Early Antenatal Prediction of Gestational Diabetes in Obese Women: Development of Prediction Tools for Targeted Intervention. *PLoS One*. 2016;11(12):e0167846. doi:10.1371/journal.pone.0167846

12. Thaware PK, Patterson CC, Young IS, Casey C, McCance DR. Clinical utility of ultrasonography-measured visceral adipose tissue depth as a tool in early pregnancy screening for gestational diabetes: a proof-of-concept study. *Diabet Med*. Jul 2019;36(7):898-901. doi:10.1111/dme.13906

13. Kennedy NJ, Peek MJ, Quinton AE, et al. Maternal abdominal subcutaneous fat thickness as a predictor for adverse pregnancy outcome: a longitudinal cohort study. *BJOG*. Jan 2016;123(2):225-32. doi:10.1111/1471-0528.13758

14. Suresh A, Liu A, Poulton A, et al. Comparison of maternal abdominal subcutaneous fat thickness and body mass index as markers for pregnancy outcomes: A stratified cohort study. *Aust N Z J Obstet Gynaecol*. Oct 2012;52(5):420-6. doi:10.1111/j.1479-828X.2012.01471.x

15. Alvarado FL, O'Tierney-Ginn P, Catalano P. Contribution of Gestational Weight Gain on Maternal Glucose Metabolism in Women with GDM and Normal Glucose Tolerance. *J Endocr Soc*. Feb 1 2021;5(2):bvaa195. doi:10.1210/jendso/bvaa195

16. Iqbal R, Rafique G, Badruddin S, Qureshi R, Cue R, Gray-Donald K. Increased body fat percentage and physical inactivity are independent predictors of gestational diabetes mellitus in South Asian women. *Eur J Clin Nutr*. Jun 2007;61(6):736-42. doi:10.1038/sj.ejcn.1602574

17. Lacroix M, Battista MC, Doyon M, et al. Lower adiponectin levels at first trimester of pregnancy are associated with increased insulin resistance and higher risk of developing gestational diabetes mellitus. *Diabetes Care*. Jun 2013;36(6):1577-83. doi:10.2337/dc12-1731

18. Liu Y, Liu J, Gao Y, et al. The Body Composition in Early Pregnancy is Associated with the Risk of Development of Gestational Diabetes Mellitus Late During the Second Trimester. *Diabetes Metab Syndr Obes*. 2020;13:2367-2374. doi:10.2147/DMSO.S245155

19. Zhang RY, Wang L, Zhou W, et al. Measuring maternal body composition by biomedical impedance can predict risk for gestational diabetes mellitus: a retrospective study among 22,223 women. *J Matern Fetal Neonatal Med*. Jul 28 2020:1-8. doi:10.1080/14767058.2020.1797666

20. Egan AM, Vellinga A, Harreiter J, et al. Epidemiology of gestational diabetes mellitus according to IADPSG/WHO 2013 criteria among obese pregnant women in Europe. *Diabetologia*. Oct 2017;60(10):1913-1921. doi:10.1007/s00125-017-4353-9

21. Hancerliogullari N, Kansu-Celik H, Asli Oskovi-Kaplan Z, Kisa B, Engin-Ustun Y, Ozgu-Erdinc AS. Optimal maternal neck and waist circumference cutoff values for prediction of gestational diabetes mellitus at the first trimester in Turkish population; a prospective cohort study. *Gynecol Endocrinol*. Nov 2020;36(11):1002-1005. doi:10.1080/09513590.2020.1750003

22. He F, He H, Liu W, et al. Neck circumference might predict gestational diabetes mellitus in Han Chinese women: A nested case-control study. *J Diabetes Investig*. Mar 2017;8(2):168-173. doi:10.1111/jdi.12574

23. Maitland RA, Seed PT, Briley AL, et al. Prediction of gestational diabetes in obese pregnant women from the UK Pregnancies Better Eating and Activity (UPBEAT) pilot trial. *Diabet Med*. Aug 2014;31(8):963-70. doi:10.1111/dme.12482

24. Yamamoto S, Douchi T, Yoshimitsu N, Nakae M, Nagata Y. Waist to hip circumference ratio as a significant predictor of preeclampsia, irrespective of overall adiposity. *J Obstet Gynaecol Res*. Feb 2001;27(1):27-31. doi:10.1111/j.1447-0756.2001.tb01211.x

25. Taebi M, Sadat Z, Saberi F, Kalahroudi MA. Early pregnancy waist-to-hip ratio and risk of preeclampsia: a prospective cohort study. *Hypertens Res*. Jan 2015;38(1):80-3. doi:10.1038/hr.2014.133

26. Sween LK, Althouse AD, Roberts JM. Early-pregnancy percent body fat in relation to preeclampsia risk in obese women. *Am J Obstet Gynecol*. Jan 2015;212(1):84 e1-7. doi:10.1016/j.ajog.2014.07.055

27. Kausar HM, Dabhadkar S, Mehendale SS, Kulkarni Y. Waist circumference,BMI,Lipid profile between 6-16 weeks of pregnancy as predictors of Gestational hypertension & Pre-eclampsia. *Indian journal of applied research*. 2011;3:274-276.

28. Sina M, Hoy W, Wang Z. Anthropometric predictors of gestational hypertensive disorders in a remote aboriginal community: a nested case-control study. *BMC Res Notes*. Mar 5 2014;7:122. doi:10.1186/1756-0500-7-122

29. Oriji VK, Ojule JD, Fumudoh BO. Prediction of Gestational Diabetes Mellitus in Early Pregnancy: Is Abdominal Skin Fold Thickness 20 mm or More an Independent Risk Predictor? *Journal of Biosciences and Medicines*. 2017;05(11):13-26. doi:10.4236/jbm.2017.511003

30. Ebrahimi-Mameghani M, Mehrabi E, Kamalifard M, Yavarikia P. Correlation between Body Mass Index and Central Adiposity with Pregnancy Complications in Pregnant Women. *Health Promot Perspect*. 2013;3(1):73-9. doi:10.5681/hpp.2013.009

31. Han Q, Shao P, Leng J, et al. Interactions between general and central obesity in predicting gestational diabetes mellitus in Chinese pregnant women: A prospective population-based study in Tianjin, China. *J Diabetes*. Jan 2018;10(1):59-67. doi:10.1111/1753-0407.12558

32. Mendoza LC, Harreiter J, Simmons D, et al. Risk factors for hyperglycemia in pregnancy in the DALI study differ by period of pregnancy and OGTT time point. *Eur J Endocrinol*. Jul 2018;179(1):39-49. doi:10.1530/EJE-18-0003

33. Gagliardi G, Tiralongo GM, LoPresti D, et al. Screening for pre-eclampsia in the first trimester: role of maternal hemodynamics and bioimpedance in non-obese patients. *Ultrasound Obstet Gynecol*. Nov 2017;50(5):584-588. doi:10.1002/uog.17379

34. Alves JG, Souza ASR, Figueiroa JN, de Araujo CAL, Guimaraes A, Ray JG. Visceral Adipose Tissue Depth in Early Pregnancy and Gestational Diabetes Mellitus - a Cohort Study. *Sci Rep*. Feb 6 2020;10(1):2032. doi:10.1038/s41598-020-59065-5

35. Bai M, Susic D, O'Sullivan AJ, Henry A. Reproducibility of Bioelectrical Impedance Analysis in Pregnancy and the Association of Body Composition with the Risk of Gestational Diabetes: A Substudy of MUMS Cohort. *J Obes*. 2020;2020:3128767. doi:10.1155/2020/3128767

36. Bartha JL, Marín-Segura P, González-González NL, Wagner F, Aguilar-Diosdado M, Hervias-Vivancos B. Ultrasound evaluation of visceral fat and metabolic risk factors during early pregnancy. *Obesity (Silver Spring)*. Sep 2007;15(9):2233-9. doi:10.1038/oby.2007.265

37. Bourdages M, Demers ME, Dube S, et al. First-Trimester Abdominal Adipose Tissue Thickness to Predict Gestational Diabetes. *J Obstet Gynaecol Can*. Jul 2018;40(7):883-887. doi:10.1016/j.jogc.2017.09.026

38. Brisson D, Perron P, Kahn HS, Gaudet D, Bouchard L. The lipid accumulation product for the early prediction of gestational insulin resistance and glucose dysregulation. *J Womens Health (Larchmt)*. Apr 2013;22(4):362-7. doi:10.1089/jwh.2012.3807

39. Campbell SK, Lynch J, Esterman A, McDermott R. Pre-pregnancy predictors of diabetes in pregnancy among Aboriginal and Torres Strait Islander women in North Queensland, Australia. *Matern Child Health J*. Aug 2012;16(6):1284-92. doi:10.1007/s10995-011-0889-3

40. Catov JM, Sun B, Bertolet M, et al. Changes in Cardiometabolic Risk Factors Before and After Gestational Diabetes: A Prospective Life-Course Analysis in CARDIA Women. *Obesity (Silver Spring)*. Aug 2020;28(8):1397-1404. doi:10.1002/oby.22848

41. Dakshnamurthy P, Thangiah Kasirajan SG, Nachiyar I, Geetha M, Rajendran K. A study of visceral adiposity index as a diagnostic index in gestational diabetes mellitus in a tertiary care centre. *International Journal of Reproduction, Contraception, Obstetrics and Gynecology*. 2017;6(4)doi:10.18203/2320-1770.ijrcog20171435

42. De Souza LR, Kogan E, Berger H, et al. Abdominal Adiposity and Insulin Resistance in Early Pregnancy. *Journal of Obstetrics and Gynaecology Canada*. 2014;36(11):969-975. doi:10.1016/s1701-2163(15)30409-6

43. De Souza LR, Berger H, Retnakaran R, et al. First-Trimester Maternal Abdominal Adiposity Predicts Dysglycemia and Gestational Diabetes Mellitus in Midpregnancy. *Diabetes Care*. Jan 2016;39(1):61-4. doi:10.2337/dc15-2027

44. De Souza LR, Berger H, Retnakaran R, et al. Hepatic fat and abdominal adiposity in early pregnancy together predict impaired glucose homeostasis in mid-pregnancy. *Nutr Diabetes*. Sep 19 2016;6(9):e229. doi:10.1038/nutd.2016.39

45. Endeshaw M, Abebe F, Worku S, Menber L, Assress M, Assefa M. Obesity in young age is a risk factor for preeclampsia: a facility based case-control study, northwest Ethiopia. *BMC Pregnancy Childbirth*. Aug 19 2016;16:237. doi:10.1186/s12884-016-1029-2

46. Gao X, Yan Y, Xiang S, et al. The mutual effect of pre-pregnancy body mass index, waist circumference and gestational weight gain on obesity-related adverse pregnancy outcomes: A birth cohort study. *PLoS One*. 2017;12(6):e0177418. doi:10.1371/journal.pone.0177418

47. Gur EB, Ince O, Turan GA, et al. Ultrasonographic visceral fat thickness in the first trimester can predict metabolic syndrome and gestational diabetes mellitus. *Endocrine*. Nov 2014;47(2):478-84. doi:10.1007/s12020-013-0154-1

48. Harville EW, Juonala M, Viikari JS, Raitakari OT. Preconception metabolic indicators predict gestational diabetes and offspring birthweight. *Gynecol Endocrinol*. Nov 2014;30(11):840-4. doi:10.3109/09513590.2014.937336

49. Ianniello F, Quagliozzi L, Caruso A, Paradisi G. Low adiponectin in overweight/obese women: association with diabetes during pregnancy. *Eur Rev Med Pharmacol Sci*. Dec 2013;17(23):3197-205.

50. KhushBakht D, Mazhar S, Bhalli A, Rashid A, Khan K, Jahanzaib U. Correlation Between Neck Circumference and Gestational Diabetes Mellitus and Associated Risk Factors During Pregnancy. *Cureus*. May 28 2018;10(5):e2699. doi:10.7759/cureus.2699

51. Lacroix M, Battista MC, Doyon M, et al. Higher maternal leptin levels at second trimester are associated with subsequent greater gestational weight gain in late pregnancy. *BMC Pregnancy Childbirth*. Mar 22 2016;16:62. doi:10.1186/s12884-016-0842-y

52. Li P, Lin S, Cui J, Li L, Zhou S, Fan J. First Trimester Neck Circumference as a Predictor for the Development of Gestational Diabetes Mellitus. *Am J Med Sci*. Feb 2018;355(2):149-152. doi:10.1016/j.amjms.2017.09.012

53. Lima M, Melo ASO, Sena ASS, Barros VO, Amorim MMR. Metabolic syndrome in pregnancy and postpartum: prevalence and associated factors. *Rev Assoc Med Bras (1992)*. Dec 2019;65(12):1489-1495. doi:10.1590/1806-9282.65.12.1489

54. Martin AM, Berger H, Nisenbaum R, et al. Abdominal visceral adiposity in the first trimester predicts glucose intolerance in later pregnancy. *Diabetes Care*. Jul 2009;32(7):1308-10. doi:10.2337/dc09-0290

55. McDonnold M, Mele LM, Myatt L, et al. Waist-to-Hip Ratio versus Body Mass Index as Predictor of Obesity-Related Pregnancy Outcomes. *Am J Perinatol*. May 2016;33(6):618-24. doi:10.1055/s-0035-1569986

56. Migda M, Migda MS, Migda B, Krzyzanowska P, Wender-Ozegowska E. Components of metabolic syndrome in the first trimester of pregnancy as predictors of adverse perinatal outcome. *Ginekol Pol*. 2016;87(9):644-650. doi:10.5603/GP.2016.0060

57. Mostafavi E, Nargesi AA, Asbagh FA, et al. Abdominal obesity and gestational diabetes: the interactive role of magnesium. *Magnes Res*. Dec 2015;28(4):116-25. doi:10.1684/mrh.2015.0392

58. Piuri G, Ferrazzi E, Bulfoni C, Mastricci L, Di Martino D, Speciani AF. Longitudinal changes and correlations of bioimpedance and anthropometric measurements in pregnancy: Simple possible bed-side tools to assess pregnancy evolution. *J Matern Fetal Neonatal Med*. Dec 2017;30(23):2824-2830. doi:10.1080/14767058.2016.1265929

59. Pontual AC, Figueiroa JN, De Souza LR, Ray JG, Alves JG. Visceral Adiposity in the First Half of Pregnancy in Association with Glucose, Lipid and Insulin Profiles in Later Pregnancy: A Cohort Study. *Matern Child Health J*. Aug 2016;20(8):1720-5. doi:10.1007/s10995-016-1975-3

60. Popova P, Grineva E, Gerasimov A, Kravchuk E, Ryazantseva E, Shelepova E. The new combination of risk factors determining a high risk of gestational diabetes mellitus. *Minerva endocrinologica*. 10/07 2014;40

61. Ray JG, De Souza LR, Park AL, Connelly PW, Bujold E, Berger H. Preeclampsia and Preterm Birth Associated With Visceral Adiposity in Early Pregnancy. *J Obstet Gynaecol Can*. Feb 2017;39(2):78-81. doi:10.1016/j.jogc.2016.10.007

62. Salem W, Adler AI, Lee C, Smith G. Maternal waist to hip ratio is a risk factor for macrosomia. *BJOG : an international journal of obstetrics and gynaecology*. 02/01 2012;119:291-7. doi:10.1111/j.1471-0528.2011.03167.x

63. Sattar N, Clark P, Holmes A, Lean ME, Walker I, Greer IA. Antenatal waist circumference and hypertension risk. *Obstet Gynecol*. Feb 2001;97(2):268-71. doi:10.1016/s0029-7844(00)01136-4

64. Sharadha SO, Punithavathi N, Renuka Devi TK. Better Predictor of Adverse Pregnancy Outcome: Asian or WHO International Cutoff? A Single-Centre Prospective Study. *J Obstet Gynaecol India*. Oct 2016;66(Suppl 1):181-6. doi:10.1007/s13224-015-0824-4

65. Tomedi LE, Simhan HN, Chang CC, McTigue KM, Bodnar LM. Gestational weight gain, early pregnancy maternal adiposity distribution, and maternal hyperglycemia. *Matern Child Health J*. Jul 2014;18(5):1265-70. doi:10.1007/s10995-013-1361-3

66. Vieira MC, Poston L, Fyfe E, et al. Clinical and biochemical factors associated with preeclampsia in women with obesity. *Obesity (Silver Spring)*. Feb 2017;25(2):460-467. doi:10.1002/oby.21715

67. Wang Y, Qiu J, Zhou M, Wang Y, Du Y. Increasing maternal percentage body fat in early second trimester: a risk factor for preeclampsia. *J Matern Fetal Neonatal Med*. Feb 2015;28(3):293-6. doi:10.3109/14767058.2014.916265

68. Wen SW, Tan H, Retnakaran R, et al. Pre-gravid predictors of new onset hypertension in pregnancy - Results from a pre-conception cohort study in China. *Eur J Obstet Gynecol Reprod Biol*. Jul 2017;214:140-144. doi:10.1016/j.ejogrb.2017.05.008

69. Yang SH, Kim C, An HS, An H, Lee JS. Prediction of Gestational Diabetes Mellitus in Pregnant Korean Women Based on Abdominal Subcutaneous Fat Thickness as Measured by Ultrasonography. *Diabetes Metab J*. Dec 2017;41(6):486-491. doi:10.4093/dmj.2017.41.6.486

70. Yeboah FA, Ngala RA, Bawah AT, et al. Adiposity and hyperleptinemia during the first trimester among pregnant women with preeclampsia. *Int J Womens Health*. 2017;9:449-454. doi:10.2147/IJWH.S134088

71. Li S, Rosenberg L, Palmer JR, Phillips GS, Heffner LJ, Wise LA. Central Adiposity and Other Anthropometric Factors in Relation to Risk of Macrosomia in an African American Population. *Obesity*. 2012;doi:10.1038/oby.2012.142
